# Novel zerumbone-secondary amide hybrids: ultrasonic synthesis, cytotoxic evaluation, molecular docking and *in silico* ADMET studies†

**DOI:** 10.1039/d5ra01215e

**Published:** 2025-03-25

**Authors:** Pham The Chinh, Pham Thi Tham, Vu Thi Lien, Dao Thi Nhung, Le Thi Thuy Loan, Vu Thi Thu Le, Vu Tuan Kien, Cao Thanh Hai, Phan Thanh Phuong

**Affiliations:** a Thai Nguyen University of Sciences – TNU Tan Thinh 24000 Thai Nguyen Vietnam chinhpt@tnus.edu.vn vuthilien203@gmail.com; b Hanoi University of Industry Cau Dien, Bac Tu Liem Hanoi Vietnam phamthitham85@haui.edu.vn +84 988113933; c VNU University of Science, Vietnam National University, Hanoi 334 Nguyen Trai Street, Thanh Xuan Ha Noi Vietnam; d Tay Nguyen University Le Duan Buon Ma Thuat Dak Lak Vietnam; e Thai Nguyen University of Agriculture and Forestry – TNU Quyet Thang 24000 Thai Nguyen Vietnam

## Abstract

Zerumbone, along with its various derivatives and structurally related compounds, has attracted significant scientific interest due to its broad-spectrum pharmacological properties, particularly its anticancer potential. In this study, novel zerumbone-secondary amide hybrids were successfully designed and synthesized with high yields using both conventional and ultrasonic methods. Reactions performed under ultrasonic conditions required significantly shorter reaction times than those conducted without ultrasound while maintaining comparable product yields. The cytotoxicity of the synthesized derivatives was evaluated against four human cancer cell lines: hepatocellular carcinoma (HepG2), lung carcinoma (A549), acute leukemia (HL-60), and gastric carcinoma (AGS). Most derivatives exhibited significant cytotoxic activity, with those derived from azazerumbone 2 demonstrating greater potency than those derived from azazerumbone 1. The incorporation of secondary amide groups has been confirmed to enhance the cytotoxic activity of the newly synthesized derivatives against cancer cells. Notably, compounds 4c, 4g, and 4i displayed the strongest cytotoxicity across all tested cell lines, with IC_50_ values ranging from 0.81 ± 0.04 to 4.14 ± 0.44 μg mL^−1^, comparable to those of zerumbone and ellipticine. Docking studies revealed a strong correlation between the biological activity of zerumbone-secondary amide hybrids and their binding affinity to EGFR tyrosine kinase, further highlighting the crucial role of secondary amide groups in enhancing their anticancer potential. Furthermore, pharmacokinetic predictions indicate that compounds 4c, 4g, and 4i possess favorable drug-like properties, reinforcing their potential as lead candidates for anticancer drug development.

## Abstract 2

Zerumbone, along with its various derivatives and structurally related compounds, has attracted significant scientific interest due to its broad-spectrum pharmacological properties, particularly its anticancer potential.

## Graphical abstract

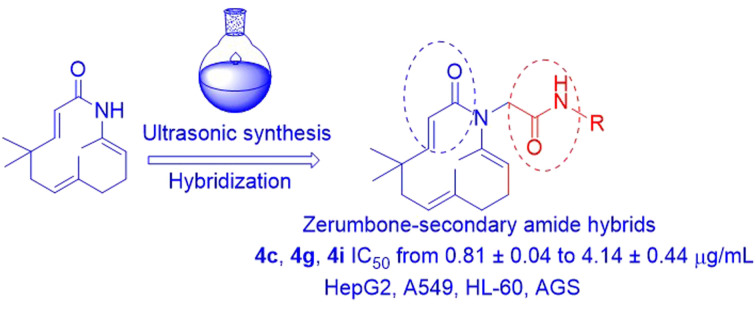

## Introduction

Zerumbone, the primary component of the essential oil derived from *Zingiber zerumbet* Sm., was first isolated in 1960.^1^ In recent years, zerumbone and its derivatives have attracted significant scientific interest due to their diverse bioactivities, including anti-cancer,^2–4^ anti-inflammatory,^5–7^ anti-HIV,^8^ antibacterial^9–11^ and anti-diabetes.^12,13^ Recent extensive research emphasizes the crucial role of the α,β-unsaturated carbonyl system in zerumbone, which acts as a key Michael acceptor and a pivotal structural feature contributing to its bioactivity, especially in inhibiting NF-κB.^7,14^ Therefore, when designing new zerumbone derivatives, preserving the α,β-unsaturated carbonyl system is essential.^15,16^

Amide derivatives play an important role in medicine and pharmacology.^17^ This functional group is found in numerous bioactive compounds with properties such as antibacterial,^18,19^ antifungal,^20^ anti-inflammatory,^21,22^ and anticancer activities.^17,18^ Recent studies have also confirmed the crucial role of secondary amide groups in exhibiting cytotoxic activity against cancer cells.^17,23^ For example, LGB321 and Vismodegib, classified as secondary derivatives, function as multi-receptor tyrosine kinase inhibitors, demonstrating potent antitumor and antiangiogenic effects in clinical trials. Due to owning significant biological activities, amide compounds have attracted considerable attention from researchers seeking novel structures with potent bioactivity.^17,24^ For instance, the secondary amide derivative (7) was designed and has exhibited significant anticancer activity.^23^

Nowadays, ultrasonic synthesis is one of the most promising approaches for performing chemical reactions under high-frequency sound waves.^25,26^ It has proven to be an effective tool for the synthesis of organic compounds, offering key advantages such as reduced reaction times, high yields, and environmental sustainability.^26^ Owing to these benefits, it has been extensively utilized in the synthesis of bioactive compounds with diverse biological functions.^20,26,27^

**Fig. 1:**
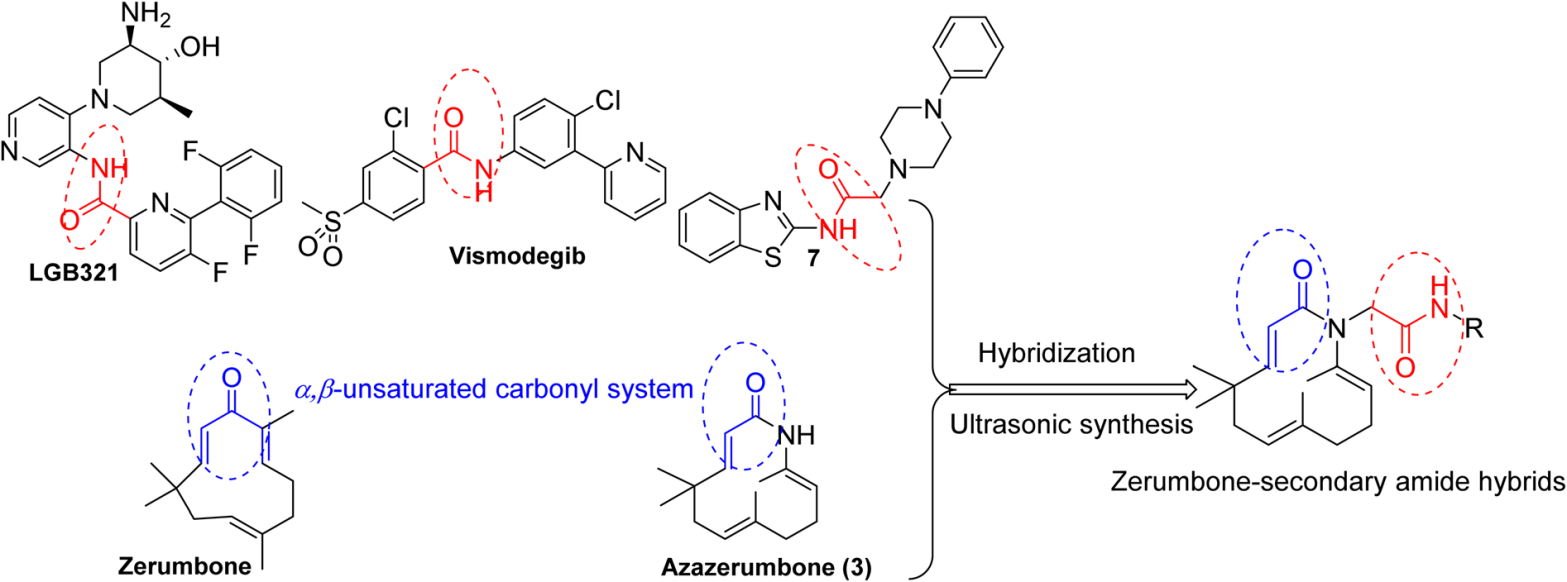
A hybridization approach for the designing of novel zerumbone-secondary amide hybrids.

In previous work,^28^ we have described the synthesis, cytotoxic evaluation, and molecular docking of novel zerumbone oxime esters and azazerumbone derivatives. Most of these derivatives exhibited significant cytotoxic effects against four human tumor cell lines. Remarkably, the modification of oxime into its corresponding oxime ester derivatives resulted in a pronounced enhancement of cytotoxic activity, showing a strong correlation between the biological activity of aromatic oxime ester derivatives and their binding affinity to the p65 subunit of NF-κB, as revealed by molecular docking studies. In this study, we continue our efforts to discover novel anticancer agents with enhanced efficacy and improved safety profiles, building upon previous research focused on developing zerumbone-based structures with superior biological activity. The strategy of merging two pharmacophores to enhance the biological and pharmacological properties of the synthesized compounds is a well-established approach in pharmaceutical and medicinal chemistry. Based on this rationale, we propose that incorporating secondary amides into a zerumbone framework may induce a synergistic effect on the molecular targets of both components, thereby facilitating the development of innovative and potentially effective anticancer agents (Fig. 1). Additionally, we explore ultrasound-assisted techniques for synthesizing these novel zerumbone-secondary amide derivatives, aiming to develop an efficient and environmentally friendly synthesis approach. Furthermore, the cytotoxic potential of these compounds is evaluated through *in vitro* assays and molecular docking studies to elucidate their interactions with specific biological targets.

## Results and discussion

### Chemistry

**Scheme 1:**
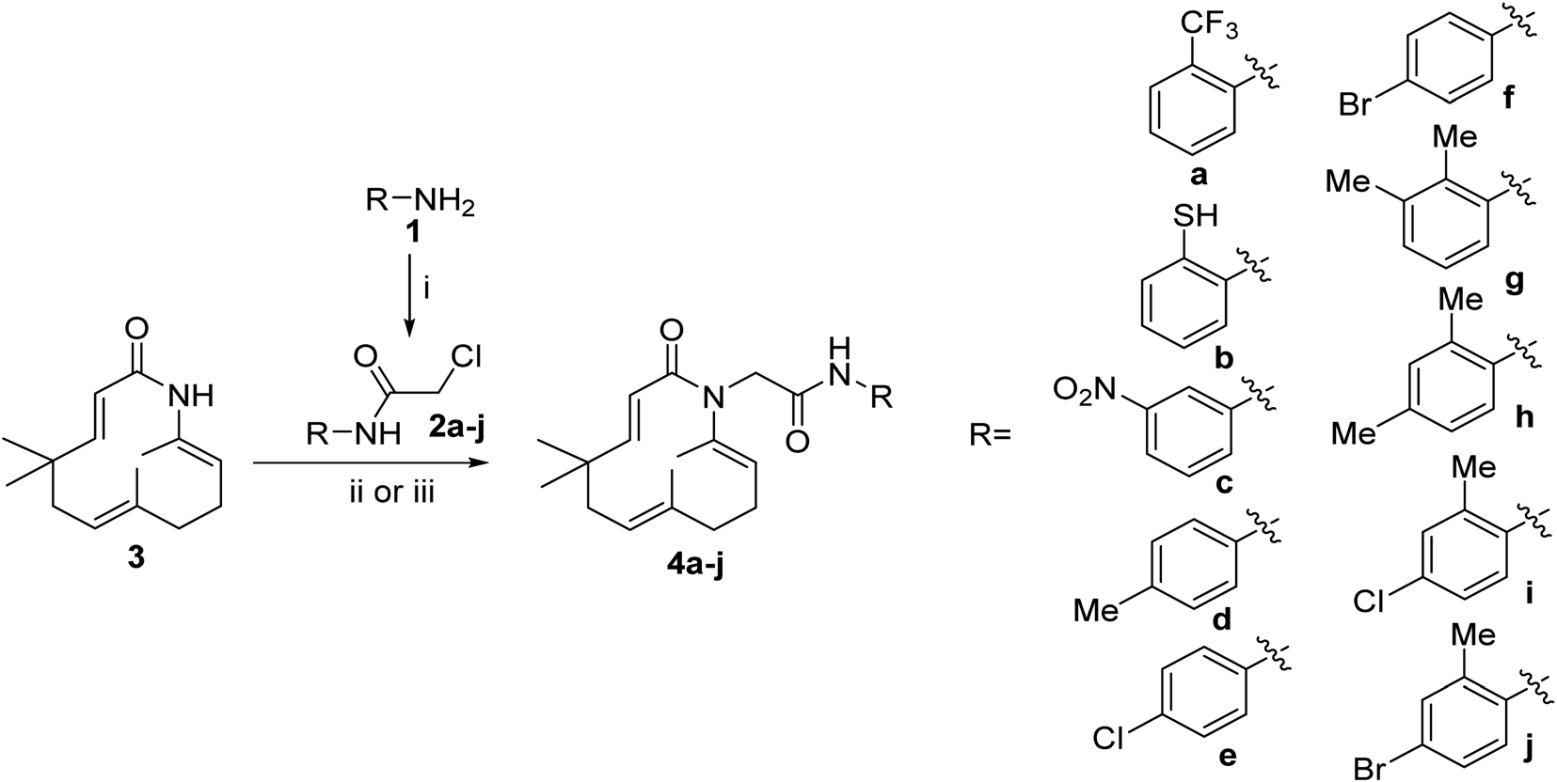
Reagents and conditions: (i), 2-chloroacetyl chloride (1.1 equiv.), saturated solution of NaHCO_3_/EtOAc 1/1, v/v, 0 °C, 1 h, 85–94%; (ii), 1.0 equiv. 2, 3.0 equiv. NaH, THF, room temperature, 36 h, 69–89%; (iii), 1.0 equiv. 2, 3.0 equiv. NaH, THF, ultrasound 40 kHz, room temperature, 2 h, 73–87%.

**Scheme 2:**
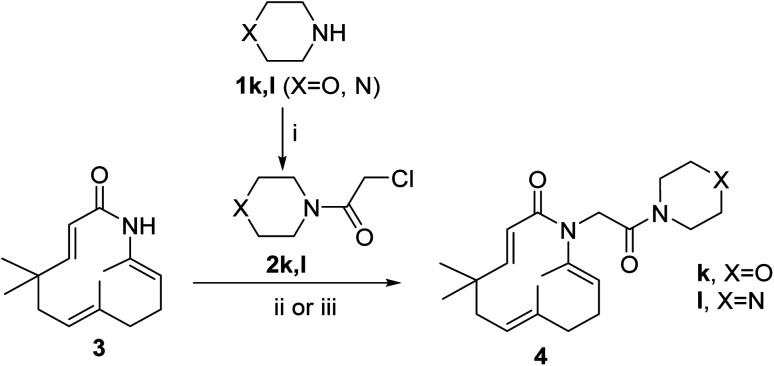
Reagents and conditions: (i), 2-chloroacetyl chloride (1.1 equiv.), saturated solution of NaHCO_3_/EtOAc 1/1 v/v, 0 °C, 1 h, 81–86%; (ii), 1.0 equiv. 2, 3.0 equiv. NaH, THF, room temperature, 36 h, 69–75%; (iii), 1.0 equiv. 2, 3.0 equiv. NaH, THF, ultrasound 40 kHz, room temperature, 2 h, 71–85%.

**Scheme 3:**
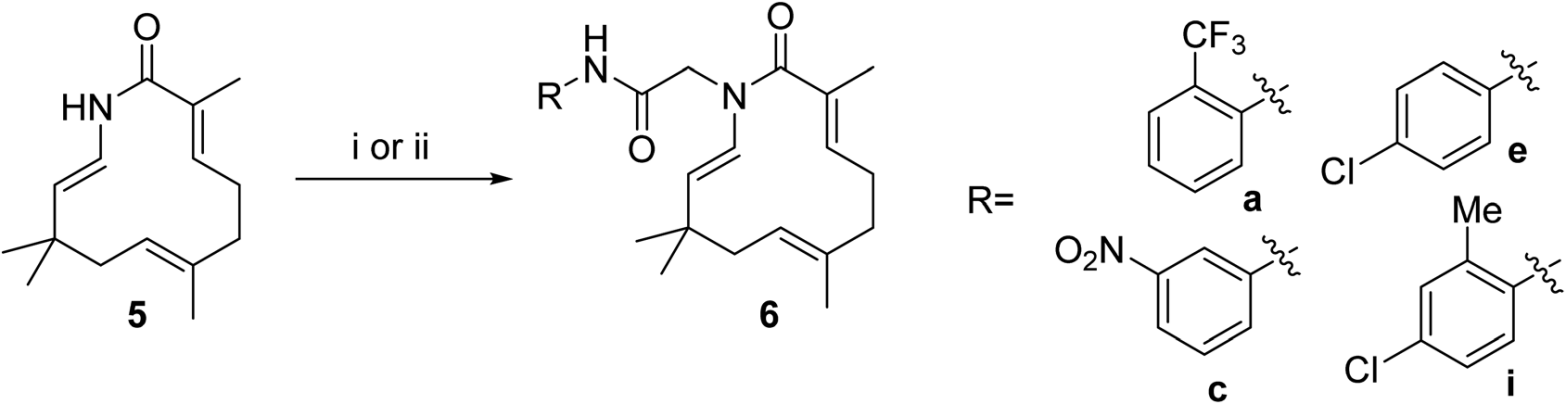
Reagents and conditions: (i), 1.0 equiv. 2, 3.0 equiv. NaH, THF, room temperature, 36 h, 75–92%; (ii), 1.0 equiv. 2, 3.0 equiv. NaH, THF, ultrasound 40 kHz, room temperature, 2 h, 68–89%.

**Scheme 4:**
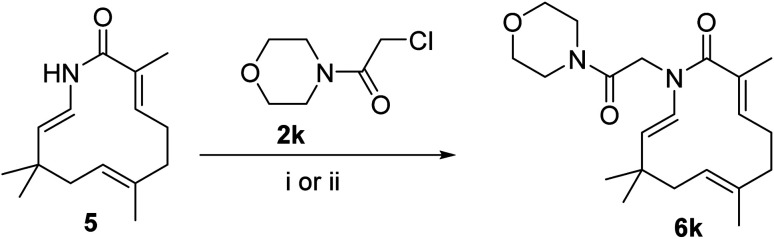
Reagents and conditions: (i), 1.0 equiv. 2k, 3.0 equiv. NaH, THF, room temperature, 36 h, 77%; (ii), 1.0 equiv. 2k, 3.0 equiv. NaH, THF, ultrasound 40 kHz, room temperature, 2 h, 69%.

Seventeen novel zerumbone amide derivatives (4a–l, and 6a, c, e, i, k) were successfully synthesized from azazerumbone 2 (3), and azazerumbone 1 (5), respectively, through a two-step process (Scheme 1–4). The starting materials, azazerumbone (3), and azazerumbone (5), were obtained from our previous research.^28^ In the first step, amides 2a–l was synthesized by the reaction of 2-chloroacetyl chloride with amines 1 in a saturated solution of NaHCO_3_/EtOAc (1 : 1, v/v), at 0 °C for 1 h, following protocol outlined in ref. 29. In the key step, zerumbone amide derivatives were synthesized *via* the reaction of azazerumbone 3 or azazerumbone 5 with amides 2a–l in THF solvent, utilizing NaH as the base under ambient temperature. The reaction was conducted under both ultrasonic and conventional conditions. Under ultrasonic conditions, the synthesis of compounds 4a–l was completed within 2 hours, achieving yields ranging from 71% to 87%. In contrast, under conventional stirring conditions, the synthesis required 36 hours at room temperature, with yields ranging from 69% to 89% (Schemes 1 and 2). Furthermore, compounds 6a, c, e, i, and k were obtained with yields of 68% to 69% under ultrasonic conditions within 2 hours, whereas conventional stirring for 36 hours resulted in higher yields of 75% to 92% (Schemes 3 and 4). The reaction conditions and yields for each compound are summarized in Table 1.

**Table 1:** The comparison between two methods to synthesize

No.	Compounds	Non-ultrasonic conditions		Ultrasonic conditions	
		Time	Yield^a^ (%)	Time	Yield^a^ (%)
1	4a	36 h	81	2 h	76
2	4b	36 h	79	2 h	83
3	4c	36 h	80	2 h	82
4	4d	36 h	84	2 h	85
5	4e	36 h	85	2 h	83
6	4f	36 h	87	2 h	85
7	4g	36 h	80	2 h	83
8	4h	36 h	83	2 h	84
9	4i	36 h	80	2 h	79
10	4j	36 h	82	2 h	87
11	4k	36 h	69	2 h	71
12	4l	36 h	75	2 h	85
13	6a	36 h	87	2 h	89
14	6c	36 h	75	2 h	68
15	6e	36 h	89	2 h	88
16	6i	36 h	92	2 h	89
17	6k	36 h	77	2 h	69

a Following purification by column chromatography.

As shown in Table 1, reactions conducted under ultrasonic conditions clearly require shorter reaction times than those performed under non-ultrasonic conditions to achieve comparable product yields. This research highlights ultrasonic synthesis as a promising method for synthesizing new zerumbone-secondary amide derivatives, offering significant time savings.

### Cytotoxicity of zerumbone-secondary amide hybrids

In the next part of this work, all compounds 4a–l, 6a, 6c, 6e, 6i and 6k were evaluated *in vitro* for their cytotoxic activity against four human cancer cell lines, including hepatoma carcinoma cell line (HepG2), human lung carcinoma (A549), human acute leukemia (HL-60), human gastric carcinoma (AGS) and the results were summarized in Table 2 (in bold are indicated the most significative results of inhibition activities). Zerumbone (1) and ellipticine were used as reference compounds for comparison. The activity values of azazerumbone 1 (5) and azazerumbone 2 (3) were obtained from the results of a previous study^28^ to compare with the standardized derivatives derived from them.

**Table 2:** The cytotoxicity evaluation (IC_50_) of novel zerumbone-amide derivatives

Compounds	IC_50_ (μg mL^−1^)			
	HepG2	A549	HL-60	AGS
4a	7.81 ± 0.25	7.49 ± 0.24	7.91 ± 0.47	8.28 ± 0.43
4b	**3.24 ± 0.23**	**5.46 ± 0.34**	6.27 ± 0.29	7.58 ± 0.17
4c	**1.81 ± 0.17**	**3.22 ± 0.27**	**3.98 ± 0.32**	**4.14 ± 0.44**
4d	6.64 ± 0.26	**5.61 ± 0.18**	7.04 ± 0.33	7.06 ± 0.45
4e	>100	>100	>100	>100
4f	>100	>100	>100	>100
4g	**2.16 ± 0.16**	**1.88 ± 0.12**	**2.24 ± 0.13**	**2.12 ± 0.15**
4h	7.09 ± 0.22	6.96 ± 0.20	7.51 ± 0.33	7.59 ± 0.34
4i	**0.81 ± 0.04**	**1.21 ± 0.05**	**0.96 ± 0.10**	**1.10 ± 0.06**
4j	**4.79 ± 0.20**	**3.98 ± 0.18**	**5.56 ± 0.45**	**5.77 ± 0.37**
4k	>100	>100	>100	>100
4l	**5.99 ± 0.23**	**5.41 ± 0.22**	**5.08 ± 0.26**	**5.76 ± 0.25**
6a	46.83 ± 3.26	39.84 ± 2.49	49.56 ± 2.75	64.92 ± 2.51
6c	45.37 ± 2.16	61.97 ± 2.88	40.17 ± 1.99	30.05 ± 2.87
6e	54.90 ± 3.13	58.95 ± 3.90	43.31 ± 1.93	56.05 ± 4.02
6i	48.75 ± 2.27	60.14 ± 4.76	51.82 ± 2.78	60.92 ± 4.50
6k	>100	>100	>100	>100
Azazerumbone 1 (5)	>100	>100	63.43 ± 2.30	>100
Azazerumbone 2 (3)	27.40 ± 2.43	56.90 ± 3.04	11.84 ± 1.20	39.64 ± 2.21
Zerumbone	**0.93 ± 0.08**	**1.03 ± 0.11**	**0.86 ± 0.04**	**1.11 ± 0.12**
Ellipticine	**0.33± 0.02**	**0.39 ± 0.03**	**0.35 ± 0.03**	**0.40 ± 0.04**

As shown in Table 2, thirteen out of the seventeen tested compounds exhibited markedly increased cytotoxic activity compared to their parent compounds (3 or 5). Notably, the zerumbone-secondary amide derivatives synthesized from azazerumbone 2 (3) displayed higher cytotoxic activity than those derived from azazerumbone 1 (5). Moreover, the conversion into zerumbone-secondary amides significantly enhanced the cytotoxic activity of azazerumbone 1 (5). Therefore, the incorporation of secondary amide groups has been confirmed to significantly enhance the cytotoxic activity of the newly synthesized derivatives against cancer cells. In particular, five compounds (4c, 4g, 4i, 4j, and 4l) demonstrated the highest cytotoxic activity across all four tested cancer cell lines. Among them, three compounds (4c, 4g, and 4i) exhibited potent cytotoxic activity comparable to that of zerumbone and ellipticine, with IC_50_ values ranging from 0.81 ± 0.04 to 4.14 ± 0.44 μg mL^−1^.

### Molecular docking study

**Fig. 2:**
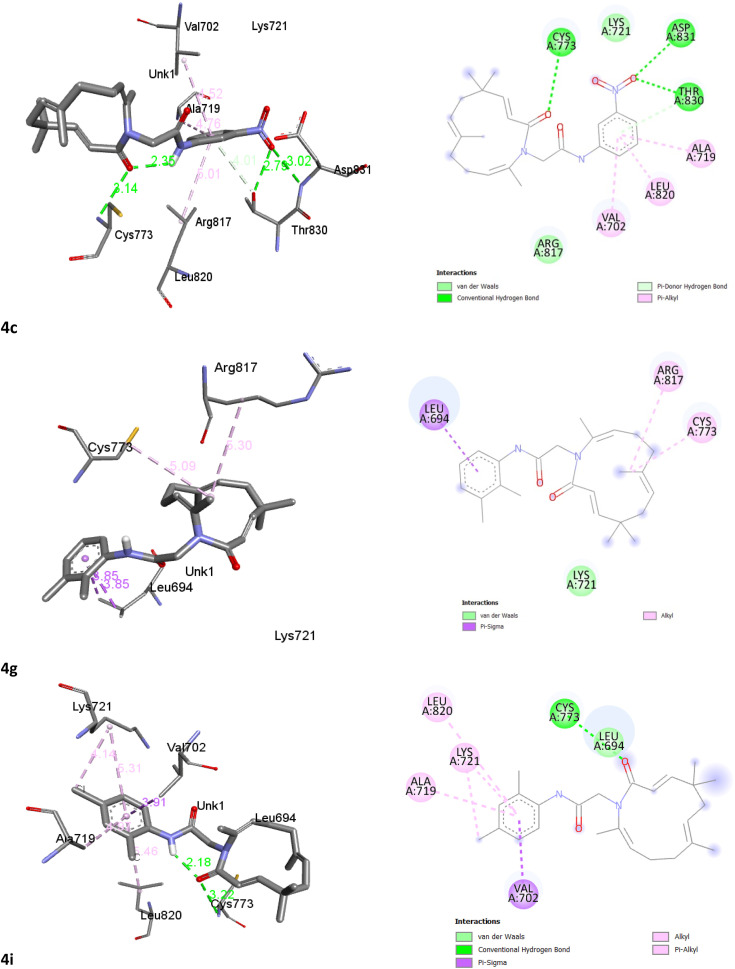
Binding interactions of ligands (4c, 4g, 4i) into the active site of receptor: 3D (left) and 2D (right).

Molecular docking serves as a crucial computational tool for characterizing protein–ligand binding sites. Based on reported experimental evidence,^23,30,31^ we conducted docking studies between EGFR tyrosine kinase and the newly synthesized zerumbone-secondary amide derivatives 4a–l. The docking results of erlotinib, zerumbone, and zerumbone-secondary amide derivatives with the receptor are presented in Table 3. Fig. 2 illustrates the binding interactions of selected ligands (4c, 4g, 4i) within the active site of the receptor.

**Table 3:** Binding energy and bond interactions

Entry	Binding energy (kcal mol^−1^)	Interacting amino acid		Total interactions
		Zerumbone part	Attached group	
Zerumbone	−6.4	Val702, Thr830, Leu694, Leu820		4
4a	−6.9	Val702, Gly695	Met769, Leu694, Leu820, Gly772	7
4b	−7.1	Val702		1
4c	**−8.0**	Cys773	Asp831, Thr830, Ala719, Leu820, Val702	7
4d	−7.3	Cys773	Val702, Thr830, Lys721, Leu764, Ala719	7
4g	**−7.8**	Arg817, Cys773	Leu694	3
4h	−6.9	Val702	Leu694, Leu768	4
4i	**−8.1**	Cys773	Val702, Lys721, Leu820, Ala719	6
4j	−7.3	Val702, Gly695	Leu694, Leu768	5
4l	−5.8	Val702, Leu820, Ala719	Gly695	4
Coligand (erlotinib)	−7.8	Met769, Cys773, Val702, Thr830, Lys721, Leu764, Ala719, Leu694, Gln767, Leu820, Pro770, Phe771		18

As presented in Table 3, the binding energy of erlotinib was −7.8 kcal mol^−1^, while that of zerumbone was −6.8 kcal mol^−1^. The binding affinities of zerumbone-secondary amide derivatives ranged from −5.8 kcal mol^−1^ to −8.1 kcal mol^−1^, with compounds 4c, 4g, and 4i demonstrating the highest affinity toward the target protein, and stronger than erlotinib, exhibiting binding energies of −8.0, −7.8, and −8.1 kcal mol^−1^, respectively. These compounds also formed similar interactions with key amino acids within the active binding sites. Furthermore, compounds 4c and 4i exhibited intramolecular hydrogen bonding between the oxygen atom in the zerumbone moiety and the hydrogen atom of the amide chain, with bond distances of 2.35 Å and 2.18 Å, respectively. These hydrogen-bonding interactions may contribute to the increased stability and enhanced binding affinity of these derivatives. Interestingly, the incorporation of polar substituent groups, particularly secondary amide groups, into the zerumbone framework appears to enhance the cytotoxic activity of these compounds. This finding supports the rationale behind the hybrid molecular design strategy, integrating zerumbone and secondary amides while preserving the α,β-unsaturated carbonyl system, which is essential for bioactivity.

A comparative analysis of cytotoxicity evaluation results (Table 2) and docking outcomes (Table 3) suggests a potential correlation between the biological activity and binding affinity of zerumbone-secondary amide derivatives with EGFR tyrosine kinase. Notably, compounds 4c, 4g, and 4i, which demonstrated the highest binding affinities, also exhibited significant cytotoxic activity. However, further experimental validation, such as molecular dynamics simulations, is required to establish a direct structure–activity relationship. Given the promising cytotoxic profile of these derivatives, this structural modification strategy offers a compelling approach for the development of novel anticancer drug candidates.

### Prediction of physicochemical and ADMET properties

**Fig. 3:**
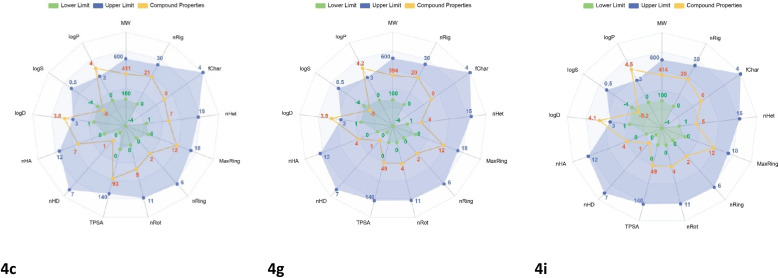
Radar diagram illustrating the physicochemical characteristics of compounds 4c, 4g, and 4i. MW: molecular weight; *n*Rig: number of rigid bonds; fChar: formal charge; *n*Het: number of heteroatoms; MaxRing: number of atoms in the largest ring; *n*Ring: number of rings; *n*Rot: number of rotatable bonds; TPSA: topological polar surface area (Å^2^); *n*HD: number of hydrogen bond donors; *n*HA: number of hydrogen bond acceptors; log *D*: logarithm of the octanol–water partition coefficient at physiological pH 7.4; log *S*: logarithm of aqueous solubility (mol L^−1^); and log *P*: logarithm of the octanol–water partition coefficient.

The activity testing results above indicate that 13 tested samples exhibit inhibitory effects against the growth of experimental cancer cells, among which three compounds (4c, 4g, and 4i) are considered potential candidates for further studies. Additionally, in the docking calculations mentioned above, further studies on molecular dynamics are also necessary. Therefore, we continued investigating the relationship between physicochemical and pharmacokinetic properties as criteria for drug-likeness evaluation. First, the radar plot of the physicochemical properties of the three hit compounds (4c, 4g, and 4i) in Fig. 3 indicates that they meet most of the criteria, except for log *P* and log *D*. Log *P* is associated with the lipophilicity of compounds and plays a crucial role in predicting drug absorption across the intestinal epithelium. According to SwissADME predictions, their log *P*_o/w_ values are below 5.0, indicating suitability for absorption.^32,33^ Furthermore, these compounds satisfy all drug-likeness criteria, with no violations of Lipinski's “Rule of Five” or other drug-likeness guidelines, including Veber's, Ghose's, and Egan's rules (Table 4).

**Table 4:** Prediction of ADMET properties of the compounds using pkCSM and Swiss ADME

Drug likeness	4c	4g	4i
Lipinski	Yes	Yes	Yes
Goshe	Yes	Yes	Yes
Veber	Yes	Yes	Yes
Egan	Yes	Yes	Yes
Muegge	Yes	No; 1 violation: XLOGP3 > 5	No; 1 violation: XLOGP3 > 5
Bioavailability score	0.55	0.55	0.55

**Absorption**			
Log *S* (mol L^−1^)	−6.189	−6.079	−6.373
Intestinal absorption (human) (% absorbed)	85.96	91.17	89.08
Caco-2 perm. (log Papp in 10^−6^ cm s^−1^	0.786	1.26	1.25

**Distribution**			
VDss (log *L* kg^−1^)	0.17	0.463	0.347
Fract. Unb. (Fu)	0	0.006	0.002
BBB perm. (log BB)	0.014	0.41	0.421

**Metabolism**			
	CYP3A4 substrate	CYP3A4 substrate	CYP3A4 substrate
	CYP2C19 inhibitor		
	CYP2C9 inhibitor		
	CYP3A4 inhibitor		

**Excretion**			
Total clearance log (ml min^−1^ kg^−1^)	1.169	1.191	−0.322
Half-life (T1/2)	0.414	0.26	0.308

**Toxicity**			
AMES toxicity	No	No	No
Rat oral acute toxicity	No (0.051)	No (0.029)	No (0.035)
Skin sensitization	Yes	Yes	Yes
hERG I inhibitor	No	No	No
hERG II inhibitor	No	No	No
EyeCorrosion	No	No	No
Respiratory	Yes	Moderate	Moderate
Eye irritation	Yes	Yes	Moderate

Second, three hit compounds were evaluated for their ADMET properties (Absorption, Distribution, Metabolism, Excretion and Toxicity) using SwissADME (Daina A, Michielin O, Zoete V. SwissADME: a free web tool to evaluate pharmacokinetics, drug-likeness and medicinal chemistry friendliness of small molecules),^34^ and pkCSM (Pires DEV, Blundell TL, Ascher DB). pkCSM: predicting small-molecule pharmacokinetic and toxicity properties using graph-based signatures^35^ (Table 4). The predicted aqueous solubility (log *S*) of three compounds reflected the solubility of compounds in an aqueous medium at 25 °C and ranged from −6.373 to −6.189, indicating a moderate solubility in water due to the presence of lipophilic groups. In addition, these synthesized compounds exhibited high intestinal absorption in the range of 85.96–91.17%. Furthermore, the absorption of orally administered drugs was evaluated based on the Caco-2 permeability value. From the model, compounds 4g and 4i have a high Caco-2 permeability with predicted values of 1.26 and 1.25, respectively. The distribution of the studied compounds was evaluated based on the volume of distribution (VDss), fraction unbound and blood–brain barrier permeability (BBB perm.). The result indicated that compound 4g was highly distributed in tissue with its log VDss of 0.463 followed by compound 4i (0.347) and 4c (0.17). All these compounds were anticipated to penetrate the brain and interact with cytochrome P450 as either substrates or inhibitors. Low clearance and a short half-life in the human body of these compounds were also observed. Anticipating toxicity showed that they are not hERG inhibitors. Additionally, they did not exhibit a mutagenic effect or eye corrosion, although they may cause reactions in the skin and respiratory systems. It can be concluded that three top-hit compounds (4c, 4g, 4i) displayed appropriate pharmacokinetic parameters and could be considered as prospective lead compounds for the development of anticancer drugs.

## Experimental

### General considerations

All reactions were performed in the appropriate oven-dried glass apparatus and under nitrogen atmosphere. Unless otherwise stated, solvents and chemicals were obtained from commercial sources and used without further purification. Column chromatography was performed using silica gel (60 Å, particle size 40–60 μm). Melting points were determined using a Buchi Melting Point B-545 and were uncorrected. Infrared (IR) analysis was conducted with a PerkinElmer Spectrum Two spectrometer using KBr pellets. NMR spectra were recorded on a Bruker Advance I (600 MHz). Chemical shifts (*δ*) are given in parts per million (ppm) and coupling constants (*J*) in hertz (Hz). High resolution mass spectrometry analysis (HRMS) was recorded on a SCIEX X500 QTOF instrument.

### General procedure for the synthesis of compounds amide 2a–l

A solution of aminde amines 1 (1.0 equivalent) in saturated solution of NaHCO_3_/EtOAc, 1/1 v/v, (10 mL) was added to the 2-chloroacetyl chloride (1.1 equivalent). The mixture was magnetically stirred at 0 °C temperature and the progress of the reaction was monitored by TLC using 10% ethyl acetate in hexane. After completion (1 h), the mixture of the reactions was concentrated and extracted with CH_2_Cl_2_. The organic phase was washed with water and saturated bine. Drying of the organic phase (MgSO_4_), filtration of the drying agent, and evaporation of the solvent *in vacuo* afforded crude compounds. The crude mass obtained was purified by column chromatography on silica gel (Hexane–EtOAc, 90 : 10) to obtain pure compounds 2a–l in pure form (85–94% yield).

#### 2-Chloro-*N*-(2-(trifluoromethyl)phenyl)acetamide (2a)

White powder, m.p. 74–75 °C. IR (KBr) *ν*_max_/cm^−1^ 3265; 3014; 2959; 2859; 1706; 1671; 1591; 1538; 1455; 1320; 1278; 1115; 1037; 789; 768. ^1^H NMR (600 MHz, CDCl_3_-*d*_1_) *δ* ppm: 8.73 (1H, bs, NH); 8.22 (1H, d, *J* = 8.4 Hz); 7.65 (1H, d, *J* = 7.2 Hz); 7.59 (1H, t, *J* = 8.4 Hz); 7.29 (1H, t, *J* = 7.2 Hz); 4.22 (2H, s). ^13^C NMR (150 MHz, CDCl_3_-*d*_1_) *δ* ppm: 164.2; 134.2; 132.9; 126.2 (q, *J* = 26 Hz); 125.2 (q, *J* = 285 Hz); 124.7; 123.9; 122.9; 42.8. HR-ESI-MS: found *m*/*z* 238.0240; calcd for C_9_H_8_ClF_3_NO^+^: 238.0241 [M + H]^+^.

#### 2-Chloro-*N*-(2-mercaptophenyl)acetamide (2b)

Orange powder, m.p. decomposition. IR (KBr) *ν*_max_/cm^−1^ 3266; 3060; 2959; 2856; 1706; 1670; 1557; 1520; 1457; 1300; 1212; 1115; 1037; 769; 745. ^1^H NMR (600 MHz, DMSO-*d*_6_) *δ* ppm: 8.10 (1H, d, *J* = 8.4 Hz); 8.02 (1H, d, *J* = 8.4 Hz); 7.54 (1H, t, *J* = 7.8 Hz); 7.46 (1H, t, *J* = 7.8 Hz); 5.22 (2H, s). ^13^C NMR (150 MHz, DMSO-*d*_6_) *δ* ppm: 166.7; 152.2; 135.2; 126.4; 125.6; 122.8; 122.2; 41.8. HR-ESI-MS: found *m*/*z* 202.0097; calcd for C_8_H_9_ClNOS^+^: 202.0092 [M + H]^+^.

#### 2-Chloro-*N*-(3-nitrophenyl)acetamide (2c)

Yellow powder, m.p. 89–90 °C. IR (KBr) *ν*_max_/cm^−1^ 3303; 3085; 2998; 2917; 2850; 1704; 1686; 1530; 1432; 1350; 1271; 1169; 739; 716. ^1^H NMR (600 MHz, CDCl_3_-*d*_1_) *δ* ppm: 8.44 (1H, bs, NH); 8.43 (1H, d, *J* = 1.8 Hz); 8.04 (1H, dd, *J* = 1.8, 8.4 Hz); 7.95 (1H, dd, *J* = 1.8, 8.4 Hz); 7.54 (1H, t, *J* = 8.4 Hz); 4.23 (2H, s). ^13^C NMR (150 MHz, CDCl_3_-*d*_1_) *δ* ppm: 164.3; 148.0; 137.8; 130.0; 125.6; 119.8; 114.9; 42.7. HR-ESI-MS: found *m*/*z* 215.0235; calcd for C_8_H_8_ClN_2_O_3_^+^: 215.0223 [M + H]^+^.

#### 2-Chloro-*N*-(*p*-tolyl)acetamide (2d)

Light yellow powder, m.p. 151–153 °C. IR (KBr) *ν*_max_/cm^−1^ 3274; 3092; 2955; 2917; 2850; 1730; 1670; 1616; 1552; 1465; 1344; 1250; 1180; 815. ^1^H NMR (600 MHz, CDCl_3_-*d*_1_) *δ* ppm: 8.15 (1H, bs, NH); 7.41 (2H, d, *J* = 8.4 Hz); 7.15 (2H, d, *J* = 8.4 Hz); 4.18 (2H, s); 2.33 (3H, s). ^13^C NMR (150 MHz, CDCl_3_-*d*_1_) *δ* ppm: 163.6; 135.0; 134.1; 129.6 (2× C); 120.2 (2× C); 42.8; 20.9. HR-ESI-MS: found *m*/*z* 184.0520; calcd for C_9_H_11_ClNO^+^: 184.0524 [M + H]^+^.

#### 2-Chloro-*N*-(4-chlorophenyl)acetamide (2e)

White powder, m.p. 129–130 °C. IR (KBr) *ν*_max_/cm^−1^ 3263; 3082; 2952; 2917; 2850; 1704; 1669; 1614; 1550; 1490; 1338; 1247; 1096; 825. ^1^H NMR (600 MHz, CDCl_3_-*d*_1_) *δ* ppm: 8.22 (2H, bs, NH); 7.50 (1H, d, *J* = 9.0 Hz); 7.32 (2H, d, *J* = 9.0 Hz); 4.20 (2H, s). ^13^C NMR (150 MHz, CDCl_3_) *δ* ppm: 163.9; 135.1; 130.4; 129.2 (2× C); 121.3 (2× C); 42.7. HR-ESI-MS: found *m*/*z* 203.9970; calcd for C_8_H_8_Cl_2_NO^+^: 203.9977 [M + H]^+^.

#### *N*-(4-Bromophenyl)-2-chloroacetamide (2f)

Light yellow powder, m.p. 170–172 °C. IR (KBr) *ν*_max_/cm^−1^ 3256; 3080; 2956; 2917; 2850; 1730; 1686; 1530; 1432; 1356; 1271; 1154; 820. ^1^H NMR (600 MHz, CDCl_3_-*d*_1_) *δ* ppm: 8.20 (1H, bs, NH); 7.46 (2H, d, *J* = 9.0 Hz); 7.45 (2H, d, *J* = 9.0 Hz); 4.18 (2H, s). ^13^C NMR (150 MHz, CDCl_3_-*d*_1_) *δ* ppm: 162.9; 136.2; 131.2; 132.1 (2× C); 121.6 (2× C); 42.8. HR-ESI-MS: found *m*/*z* 247.9482; calcd for C_8_H_8_BrClNO^+^: 247.9478 [M + H]^+^.

#### 2-Chloro-*N*-(2,3-dimethylphenyl)acetamide (2g)

Light pink powder, m.p. 64–65 °C. IR (KBr) *ν*_max_/cm^−1^ 3257; 3017; 2959; 2921; 2852; 1704; 1653; 1542; 1454; 1386; 1296; 1193; 970; 817. ^1^H NMR (600 MHz, CDCl_3_-*d*_1_) *δ* ppm: 8.19 (1H, bs, NH); 7.55 (1H, d, *J* = 7.8 Hz); 7.12 (1H, t, *J* = 7.8 Hz); 7.05 (1H, d, *J* = 7.2 Hz); 4.05 (2H, s); 2.31 (3H, s); 2.18 (3H, s). ^13^C NMR (150 MHz, CDCl_3_-*d*_1_) *δ* ppm: 163.1; 124.0; 120.7; 115.5; 114.4; 112.5; 107.9; 48.7; 29.6; 27.3. HR-ESI-MS: found *m*/*z* 198.0677; calcd for C_10_H_13_ClNO^+^: 198.0680 [M + H]^+^.

#### 2-Chloro-*N*-(2,4-dimethylphenyl)acetamide (2h)

Light brown powder, m.p. 104–105 °C. IR (KBr) *ν*_max_/cm^−1^ 3260; 3085; 2955; 2917; 2850; 1715; 1686; 1531; 1430; 1321; 1230; 1179; 829. ^1^H NMR (600 MHz, CDCl_3_-*d*_1_) *δ* ppm: 8.12 (1H, bs, NH); 7.67 (1H, d, *J* = 7.8 Hz) 7.04 (1H, d, *J* = 7.8 Hz); 7.02 (1H, s); 4.22 (2H, s); 2.30 (3H, s); 2.25 (3H, s). ^13^C NMR (150 MHz, CDCl_3_-*d*_1_) *δ* ppm: 163.8; 135.6; 131.3; 129.4; 127.4; 122.8; 43.1; 20.9; 17.4. HR-ESI-MS: found *m*/*z* 198.0676; calcd for C_10_H_13_ClNO^+^: 198.0680 [M + H]^+^.

#### 2-Chloro-*N*-(4-chloro-2-methylphenyl)acetamide (2i)

Dark purple powder, m.p. 130–131 °C. IR (KBr) *ν*_max_/cm^−1^ 3254; 3046; 2951; 2917; 2859; 1707; 1667; 1579; 1537; 1460; 1327; 1252; 1199; 816. ^1^H NMR (600 MHz, CDCl_3_-*d*_1_) *δ* ppm: 8.19 (1H, bs, NH); 8.85 (1H, d, *J* = 9.0 Hz); 7.20–7.22 (2H, m); 4.23 (2H, s); 2.28 (3H, s). ^13^C NMR (150 MHz, CDCl_3_-*d*_1_) *δ* ppm: 163.7; 133.2; 130.8; 130.7; 130.4; 126.9; 123.5; 43.1; 17.3. HR-ESI-MS: found *m*/*z* 218.0130; calcd for C_9_H_10_Cl_2_NO^+^: 218.0134 [M + H]^+^.

#### *N*-(4-Bromo-2-methylphenyl)-2-chloroacetamide (2j)

Brown powder, m.p. 68–70 °C. IR (KBr) *ν*_max_/cm^−1^ 3252; 3017; 2953; 2924; 2852; 1739; 1666; 1579; 1484; 1397; 1253; 1189; 868. ^1^H NMR (600 MHz, CDCl_3_-*d*_1_) *δ* ppm: 8.21 (1H, bs, NH); 8.85 (1H, d, *J* = 8.4 Hz); 7.16–7.17 (2H, m); 4.10 (2H, s); 2.27 (3H, s). ^13^C NMR (150 MHz, CDCl_3_-*d*_1_) *δ* ppm: 163.6; 133.1; 133.1; 132.3; 130.7; 129.4; 124.4; 42.5; 16.7. HR-ESI-MS: found *m*/*z* 261.9640; calcd for C_9_H_10_BrClNO^+^: 261.9634 [M + H]^+^.

#### 2-Chloro-1-morpholinoethan-1-one (2k)

Colourless oil. IR (KBr) *ν*_max_/cm^−1^ 3082; 2965; 2921; 2853; 1660; 1411; 1265; 1155; 848. ^1^H NMR (600 MHz, CDCl_3_-*d*_1_) *δ* ppm: 4.07 (2H, s); 3.72 (2H, t, *J* = 4.2 Hz); 3.70 (2H, t, *J* = 4.2 Hz); 3.63 (2H, t, *J* = 4.8 Hz); 3.53 (2H, t, *J* = 4.8 Hz). ^13^C NMR (150 MHz, CDCl_3_-*d*_1_) *δ* ppm: 165.2; 66.6; 66.4; 46.7; 42.4; 40.5. HR-ESI-MS: found *m*/*z* 164.0470; calcd for C_6_H_11_ClNO_2_^+^: 164.0473 [M + H]^+^.

#### 2-Chloro-1-(piperazin-1-yl)ethan-1-one (2l)

White powder, m.p. 113–114 °C. IR (KBr) *ν*_max_/cm^−1^ 3210; 3089; 2961; 2921; 2856; 1640; 1430; 1275; 1165; 820. ^1^H NMR (600 MHz, CDCl_3_-*d*_1_) *δ* ppm: 4.09 (2H, s); 3.72 (2H, bs); 3.65 (2H, bs); 3.63 (2H, bs); 3.56 (2H, bs). ^13^C NMR (150 MHz, CDCl_3_-*d*_1_) *δ* ppm: 165.4; 46.2; 45.8; 42.0; 41.7; 40.6. HR-ESI-MS: found *m*/*z* 163.0642; calcd for C_6_H_12_ClN_2_O^+^: 163.0638 [M + H]^+^.

### General procedure for the synthesis of compounds 4a–l

A solution of azazerumbone 2 (3) (1.0 equivalent) in THF (10 mL) was added to the amide 2a–l (1.0 equivalent) followed by NaH (3.0 equivalent) at 0 °C temperature. The mixture was magnetically stirred at room temperature and the progress of the reaction was monitored by TLC using 20% ethyl acetate in hexane. After completion (36 h), the mixture of the reactions was concentrated and extracted with CH_2_Cl_2_. The organic phase was washed with water and saturated bine. Drying of the organic phase (MgSO_4_), filtration of the drying agent, and evaporation of the solvent *in vacuo* afforded crude compounds. The crude mass obtained was purified by column chromatography on silica gel (Hexane–EtOAc, 90 : 10) to obtain pure compounds 4a–l in pure form (69–89% yield).

### General procedure for the ultrasonic synthesis of compounds 4a–l

A solution of azazerumbone 2 (3) (1.0 equivalent) in THF (10 mL) was added to the amide 2a–l (1.0 equivalent) followed by NaH (3.0 equivalent) at 0 °C temperature. The mixture was carried out in ultrasonic condition at room temperature and 40 kHz. After completion (2 h), the mixture of the reactions was concentrated and extracted with CH_2_Cl_2_. The organic phase was washed with water and saturated bine. Drying of the organic phase (MgSO_4_), filtration of the drying agent, and evaporation of the solvent *in vacuo* afforded crude compounds. The crude mass obtained was purified by column chromatography on silica gel (Hexane–EtOAc, 90 : 10) to obtain pure compounds 4a–l in pure form (71–87% yield).

#### 2-((3*E*,7*E*,11*E*)-5,5,8,12-Tetramethyl-2-oxoazacyclododeca-3,7,11-trien-1-yl)-*N*-(2-(trifluoromethyl)phenyl)acetamide (4a)

Colourless oil. IR (KBr) *ν*_max_/cm^−1^ 3217; 3083; 2955; 2917; 2848; 1717; 1620; 1532; 1425; 1380; 1200; 1065; 974; 759; 730. ^1^H NMR (600 MHz, CDCl_3_-*d*_1_) *δ* ppm: 8.89 (1H, bs, NH); 8.15 (1H, d, *J* = 9.6 Hz); 7.60 (1H, d, *J* = 9.6 Hz); 7.52 (1H, t, *J* = 9.6 Hz); 7.21 (1H, t, *J* = 9.6 Hz); 6.54 (1H, d, *J* = 19.2 Hz); 5.91 (1H, d, *J* = 19.2 Hz); 4.22 (2H, s); 2.12–2.46 (6H, m); 1.87 (3H, s); 1.60 (3H, s); 1.13 (6H, s). ^13^C NMR (150 MHz, CDCl_3_-*d*_1_) *δ* ppm: 168.7; 168.2; 151.2; 135.2; 133.9; 133.6; 132.7; 126.2; 126.1; 125.5 (q, 26 Hz); 125.2; 125.1; 124.6; 122.0 (q, *J* = 285 Hz); 121.8; 50.9; 39.5; 38.9; 37.2; 36.8; 29.5; 25.6; 15.3; 15.2. HR-ESI-MS: found *m*/*z* 435.2234; calcd for C_24_H_29_F_3_N_2_O_2_^+^: 435.2254 [M + H]^+^.

#### *N*-(2-Mercaptophenyl)-2-((3*E*,7*E*,11*E*)-5,5,8,12-tetramethyl-2-oxoazacyclododeca-3,7,11-trien-1-yl)acetamide (4b)

Yellow-brown powder, m.p. 127–129 °C. IR (KBr) *ν*_max_/cm^−1^ 3320; 3093; 2957; 2917; 2850; 1730; 1621; 1593; 1460; 1378; 1180; 759; 722. ^1^H NMR (600 MHz, CDCl_3_-*d*_1_) *δ* ppm: 7.95 (1H, d, *J* = 7.8 Hz); 7.82 (1H, d, *J* = 7.8 Hz); 7.44 (1H, t, *J* = 7.2 Hz); 7.35 (1H, t, *J* = 7.2 Hz); 6.52 (1H, d, *J* = 15.6 Hz); 5.91 (1H, d, *J* = 15.6 Hz); 5.06 (1H, t, *J* = 7.2 Hz); 5.00 (1H, t, *J* = 6.0 Hz); 2.10–2.30 (6H, m); 1.88 (3H, s); 1.59 (3H, s); 1.12 (6H, s). ^13^C NMR (150 MHz, CDCl_3_-*d*_1_) *δ* ppm: 168.7; 167.3; 152.4; 150.0; 136.0; 133.9; 133.7; 133.5; 125.8; 125.1; 125.0; 122.9; 122.3; 121.6; 46.9; 39.4; 38.7 (2× C); 36.7; 29.8; 25.5; 15.3; 15.2. HR-ESI-MS: found *m*/*z* 399.2114; calcd for C_23_H_31_N_2_O_2_S^+^: 399.2106 [M + H]^+^.

#### *N*-(3-Nitrophenyl)-2-((3*E*,7*E*,11*E*)-5,5,8,12-tetramethyl-2-oxoazacyclododeca-3,7,11-trien-1-yl)acetamide (4c)

Yellow powder, m.p. 94–95 °C. IR (KBr) *ν*_max_/cm^−1^ 3350; 3089; 2956; 2917; 2850; 1730; 1610; 1570; 1467; 1349; 1294; 1180; 737. ^1^H NMR (600 MHz, CDCl_3_-*d*_1_) *δ* ppm: 9.55 (1H, bs, NH); 8.42 (1H, t, *J* = 1.8 Hz); 7.93 (1H, dd, *J* = 1.8, 7.2 Hz); 7.85 (1H, dd, *J* = 1.8, 7.2 Hz); 7.45 (1H, t, *J* = 8.4 Hz); 6.53 (1H, d, *J* = 16.2 Hz); 5.92 (1H, d, *J* = 16.2 Hz); 5.13 (1H, t, *J* = 7.2 Hz); 4.99 (1H, t, *J* = 6.0 Hz); 4.18 (2H, s); 2.10–2.40 (6H, m); 1.90 (3H, s); 1.60 (3H, s); 1.13 (6H, s). ^13^C NMR (150 MHz, CDCl_3_-*d*_1_) *δ* ppm: 169.1; 168.8; 151.2; 134.2; 134.1; 133.5; 133.2; 129.6; 129.5; 125.4; 125.0; 121.9; 118.6; 114.6; 51.7; 39.4; 38.7; 36.8 (2× C); 29.5; 25.5; 15.2; 15.1. HR-ESI-MS: found *m*/*z* 412.2220; calcd for C_23_H_30_N_3_O_4_^+^: 412.2230 [M + H]^+^.

#### 2-((3*E*,7*E*,11*E*)-5,5,8,12-Tetramethyl-2-oxoazacyclododeca-3,7,11-trien-1-yl)-*N*-(*p*-tolyl)acetamide (4d)

White powder, m.p. 158–160 °C. IR (KBr) *ν*_max_/cm^−1^ 3320; 3089; 2955; 2919; 2848; 1712; 1603; 1597; 1489; 1390; 1221; 1169; 830. ^1^H NMR (600 MHz, CDCl_3_-*d*_1_) *δ* ppm: 8.92 (1H, bs, NH); 7.40 (2H, d, *J* = 8.4 Hz); 7.10 (2H, d, *J* = 8.4 Hz); 6.48 (1H, d, *J* = 15.6 Hz); 5.90 (1H, d, *J* = 15.6 Hz); 5.13 (1H, t, *J* = 7.8 Hz); 4.98 (1H, t, *J* = 6.0 Hz); 4.20 (2H, s); 2.30 (3H, s); 2.18–2.32 (6H, m); 1.88 (3H, s); 1.59 (3H, s); 1.11 (6H, s). ^13^C NMR (150 MHz, CDCl_3_-*d*_1_) *δ* ppm: 168.6; 167.4; 150.6; 135.5; 134.3; 133.3; 130.0; 129.3 (2× C); 124.9; 122.2; 119.9 (2× C); 113.7; 51.4; 39.4; 38.7; 36.7; 31.9; 29.7; 25.5; 20.8; 15.2; 15.0. HR-ESI-MS: found *m*/*z* 381.2520; calcd for C_24_H_33_N_2_O_2_^+^: 381.2536 [M + H]^+^.

#### *N*-(4-Chlorophenyl)-2-((3*E*,7*E*,11*E*)-5,5,8,12-tetramethyl-2-oxoazacyclododeca-3,7,11-trien-1-yl)acetamide (4e)

White powder, m.p. 235–237 °C. IR (KBr) *ν*_max_/cm^−1^ 3330; 3092; 2955; 2919; 2849; 1711; 1620; 1593; 1483; 1376; 1217; 1174; 1074; 829. ^1^H NMR (600 MHz, CDCl_3_-*d*_1_) *δ* ppm: 9.16 (1H, s, NH); 7.46 (2H, dd, *J* = 1.8; 7.2 Hz); 7.25 (2H, dd, *J* = 1.8; 7.2 Hz); 6.49 (1H, d, *J* = 15.6 Hz); 5.90 (1H, d, *J* = 15.6 Hz); 5.12 (1H, t, *J* = 7.2 Hz); 4.98 (1H, t, *J* = 6.0 Hz); 4.12 (2H, bs); 2.15–2.32 (6H, m); 1.88 (3H, s); 1.60 (3H, s); 1.54 (6H, s). ^13^C NMR (150 MHz, CDCl_3_-*d*_1_) *δ* ppm: 168.8; 167.6; 150.8; 136.6; 134.3; 134.0; 133.4; 129.0; 128.9 (2× C); 124.9; 122.0; 121.1 (2× C); 51.6; 39.4; 38.7; 36.7; 29.7 (2× C); 25.5; 15.2; 15.0. HR-ESI-MS: found *m*/*z* 401.1990; calcd for C_23_H_30_ClN_2_O_2_^+^: 401.1991 [M + H]^+^.

#### *N*-(4-Bromophenyl)-2-((3*E*,7*E*,11*E*)-5,5,8,12-tetramethyl-2-oxoazacyclododeca-3,7,11-trien-1-yl)acetamide (4f)

White powder, m.p. 226–228 °C. IR (KBr) *ν*_max_/cm^−1^ 3327; 3099; 2955; 2919; 2849; 1709; 1621; 1593; 1489; 1395; 1216; 1174; 829. ^1^H NMR (600 MHz, CDCl_3_-*d*_1_) *δ* ppm: 9.16 (1H, s, NH); 7.42 (2H, d, *J* = 8.5 Hz); 7.41 (2H, d, *J* = 8.5 Hz); 6.49 (1H, d, *J* = 15.6 Hz); 5.90 (1H, d, *J* = 15.6 Hz); 5.12 (1H, t, *J* = 7.8 Hz); 4.98 (1H, t, *J* = 6.0 Hz); 4.11 (2H, s); 2.15–2.32 (6H, m); 1.88 (3H, s); 1.60 (3H, s); 1.12 (6H, s). ^13^C NMR (150 MHz, CDCl_3_-*d*_1_) *δ* ppm: 168.8; 167.6; 150.8; 137.1; 134.6; 134.2; 134.0; 133.4; 131.8 (2× C); 124.9; 122.0; 121.4 (2× C); 51.6; 39.4; 38.7; 36.9; 29.0; 25.5; 15.2; 15.0. HR-ESI-MS: found *m*/*z* 445.1470; calcd for C_23_H_30_BrN_2_O_2_^+^: 445.1480 [M + H]^+^.

#### *N*-(2,3-Dimethylphenyl)-2-((3*E*,7*E*,11*E*)-5,5,8,12-tetramethyl-2-oxoazacyclododeca-3,7,11-trien-1-yl)acetamide (4g)

Light brown powder, m.p. 121–123 °C. IR (KBr) *ν*_max_/cm^−1^ 3220; 3063; 2954; 2919; 2848; 1706; 1614; 1547; 1467; 1389; 1203; 1065; 974; 779; 716. ^1^H NMR (600 MHz, CDCl_3_-*d*_1_) *δ* ppm: 8.77 (1H, s, NH); 7.65 (1H, d, *J* = 8.4 Hz); 7.08 (1H, t, *J* = 7.8 Hz); 6.96 (1H, d, *J* = 7.2); 6.48 (1H, d, *J* = 16.2 Hz); 5.91 (1H, d, *J* = 16.2 Hz); 5.14 (1H, t, *J* = 7.8 Hz); 4.97 (1H, t, *J* = 6.0 Hz); 2.25–2.40 (2H, m); 2.29 (3H, s); 2.15–2.22 (4H, m); 2.16 (3H, s); 1.89 (3H, s); 1.57 (3H, s); 1.11 (6H, s). ^13^C NMR (150 MHz, CDCl_3_-*d*_1_) *δ* ppm: 168.5; 167.9; 150.7; 137.2; 135.8; 134.1; 134.0; 133.4; 127.9; 126.6; 125.7; 124.9; 122.1; 120.7; 51.1; 39.4 (2× C); 38.7; 36.7; 29.9; 25.5; 20.6; 15.2; 15.0; 13.5. HR-ESI-MS: found *m*/*z* 395.2690; calcd for C_25_H_35_N_2_O_2_^+^: 395.2693 [M + H]^+^.

#### *N*-(2,4-Dimethylphenyl)-2-((3*E*,7*E*,11*E*)-5,5,8,12-tetramethyl-2-oxoazacyclododeca-3,7,11-trien-1-yl)acetamide (4h)

White powder, m.p. 115–117 °C. IR (KBr) *ν*_max_/cm^−1^ 3322; 3090; 2955; 2919; 2848; 1710; 1620; 1593; 1483; 1390; 1245; 1170; 840. ^1^H NMR (600 MHz, CDCl_3_-*d*_1_) *δ* ppm: 8.75 (1H, bs, NH); 7.77 (1H, d, *J* = 7.8 Hz); 6.99 (1H, d, *J* = 8.4 Hz); 6.98 (1H, s); 6.47 (1H, d, *J* = 16.2 Hz); 5.90 (1H, d, *J* = 16.2 Hz); 5.12 (1H, t, *J* = 7.2 Hz); 4.97 (1H, t, *J* = 6.0 Hz); 4.13 (2H, s); 2.27 (3H, s); 2.22 (3H, s); 2.12–2.42 (6H, m); 1.89 (3H, s); 1.60 (3H, s); 1.11 (6H, s). ^13^C NMR (150 MHz, CDCl_3_-*d*_1_) *δ* ppm: 168.5; 167.7; 150.6; 134.2; 134.1; 134.0; 133.6; 133.4; 131.1; 128.3; 127.0; 124.9; 122.1; 122.0; 51.1; 39.3; 38.7; 36.6; 29.6; 25.5; 20.8; 17.8; 15.2; 15.0. HR-ESI-MS: found *m*/*z* 395.2680; calcd for C_25_H_35_N_2_O_2_^+^: 395.2693 [M + H]^+^.

#### *N*-(4-Chloro-2-methylphenyl)-2-((3*E*,7*E*,11*E*)-5,5,8,12-tetramethyl-2-oxoazacyclododeca-3,7,11-trien-1-yl)acetamide (4i)

White powder, m.p. 125–126 °C. IR (KBr) *ν*_max_/cm^−1^ 3220; 3067; 2954; 2920; 2851; 1707; 1614; 1538; 1421; 1393; 1294; 1194; 969; 817. ^1^H NMR (600 MHz, CDCl_3_-*d*_1_) *δ* ppm: 8.96 (1H, bs, NH); 7.93 (1H, d, *J* = 9.6 Hz); 7.13–7.15 (2H, m); 6.48 (1H, d, *J* = 15.6 Hz); 5.90 (1H, d, *J* = 15.6 Hz); 5.11 (1H, t, *J* = 7.2 Hz); 4.97 (1H, t, *J* = 6.0 Hz); 4.12 (2H, s); 2.24 (3H, s); 2.12–2.42 (6H, m); 1.89 (3H, s); 1.60 (3H, s); 1.11 (6H, s). ^13^C NMR (150 MHz, CDCl_3_-*d*_1_) *δ* ppm: 168.7; 167.7; 150.9; 134.9; 134.1; 134.0; 133.5; 130.1; 129.8; 129.2; 126.4; 124.9; 122.8; 122.0; 51.3; 39.3; 38.7; 36.7 (2× C); 29.4; 25.5; 17.8; 15.2; 15.0. HR-ESI-MS: found *m*/*z* 415.2130; calcd for C_24_H_32_ClN_2_O_2_^+^: 415.2147 [M + H]^+^.

#### *N*-(4-Bromo-2-methylphenyl)-2-((3*E*,7*E*,11*E*)-5,5,8,12-tetramethyl-2-oxoazacyclododeca-3,7,11-trien-1-yl)acetamide (4j)

White powder, m.p. 90–92 °C. IR (KBr) *ν*_max_/cm^−1^ 3312; 3097; 2957; 2917; 2848; 1710; 1620; 1587; 1468; 1387; 1223; 1154; 815; 716. ^1^H NMR (600 MHz, CDCl_3_-*d*_1_) *δ* ppm: 9.26 (1H, s, NH); 7.41 (1H, dd, *J* = 1.8; 8.4 Hz); 7.39 (1H, s); 7.33 (1H, dd, *J* = 1.8, 8.4 Hz); 6.20 (1H, d, *J* = 15.6 Hz); 5.90 (1H, d, *J* = 15.6 Hz); 5.14 (1H, t, *J* = 7.8 Hz); 5.01 (1H, t, *J* = 6.0 Hz); 4.13 (2H, bs); 2.08–2.40 (6H, m); 2.18 (3H, s); 1.82 (3H, s); 1.57 (3H, s); 1.05 (6H, s). ^13^C NMR (150 MHz, CDCl_3_-*d*_1_) *δ* ppm: 167.1; 165.9; 147.7; 135.6; 134.6; 133.7; 132.5; 131.4; 128.6; 126.2; 124.4; 122.6; 116.9; 115.5; 48.0; 38.8; 38.0; 36.0 (2× C); 29.0; 24.9; 17.4; 14.9; 14.8. HR-ESI-MS: found *m*/*z* 459.1630; calcd for C_24_H_32_BrN_2_O_2_^+^: 459.1642 [M + H]^+^.

#### (3*E*,7*E*,11*E*)-5,5,8,12-Tetramethyl-1-(2-morpholino-2-oxoethyl)azacyclododeca-3,7,11-trien-2-one (4k)

Colourless oil. IR (KBr) *ν*_max_/cm^−1^ 3090; 2971; 2924; 2858; 1649; 1442; 1274; 1242; 1114; 848. ^1^H NMR (600 MHz, DMSO-*d*_6_) *δ* ppm: 6.17 (1H, d, *J* = 15.6 Hz); 5.90 (1H, d, *J* = 15.6 Hz); 5.18 (1H, t, *J* = 7.2 Hz); 5.01 (1H, t, *J* = 6.0 Hz); 4.10 (2H, s); 3.50–3.56 (4H, m); 3.41–3.45 (4H, m); 2.10–2.90 (6H, m); 1.76 (3H, s); 1.57 (3H, s); 1.06 (6H, s). ^13^C NMR (150 MHz, DMSO-*d*_6_) *δ* ppm: 167.5; 166.2; 147.4; 134.4; 133.7; 131.8; 131.8; 124.4; 122.7; 68.9; 66.0; 65.7; 45.5; 44.8; 41.6; 41.4; 38.0; 36.0; 28.7; 24.9; 15.0; 14.9. HR-ESI-MS: found *m*/*z* 361.2470; calcd for C_21_H_33_N_2_O_3_^+^: 361.2485 [M + H]^+^.

#### (3*E*,7*E*,11*E*)-5,5,8,12-Tetramethyl-1-(2-oxo-2-(piperazin-1-yl)ethyl)azacyclododeca-3,7,11-trien-2-one (4l)

White powder, m.p. 79–81 °C. IR (KBr) *ν*_max_/cm^−1^ 3210; 3093; 2961; 2921; 2856; 1648; 1444; 1272; 1240; 1113; 821. ^1^H NMR (600 MHz, CDCl_3_-*d*_1_) *δ* ppm: 6.41 (1H, d, *J* = 15.6 Hz); 5.92 (1H, d, *J* = 15.6 Hz); 5.32 (1H, t, *J* = 7.8 Hz); 5.02 (1H, t, *J* = 6.0 Hz); 4.14 (2H, s); 3.57–3.61 (4H, m); 3.54–3.56 (4H, m); 2.20–2.32 (6H, m); 1.84 (3H, s); 1.60 (3H, s); 1.09 (6H, s). ^13^C NMR (150 MHz, CDCl_3_-*d*_1_) *δ* ppm: 168.1; 166.9; 149.5; 134.1; 133.3; 131.4; 125.1; 122.1; 66.9; 46.0; 45.1; 39.4; 39.0; 38.7; 37.1; 32.7; 31.9; 27.9; 22.7; 14.4; 14.1. HR-ESI-MS: found *m*/*z* 360.2659; calcd for C_21_H_34_N_3_O_2_^+^: 360.2651 [M + H]^+^.

### General procedure for the synthesis of compounds 6a, 6c, 6e, 6i, and 6k

A solution of azazerumbone 1 (5) (1.0 equivalent) in THF (10 mL) was added to the amide 2a or 2c, 2e, 2i, and 2k (1.0 equivalent) followed by NaH (3.0 equivalent) at 0 °C temperature. The mixture was magnetically stirred at room temperature and the progress of the reaction was monitored by TLC using 20% ethyl acetate in hexane. After completion (36 h), the mixture of the reactions was concentrated and extracted with CH_2_Cl_2_. The organic phase was washed with water and saturated bine. Drying of the organic phase (MgSO_4_), filtration of the drying agent, and evaporation of the solvent *in vacuo* afforded crude compounds. The crude mass obtained was purified by column chromatography on silica gel (Hexane–EtOAc, 90 : 10) to obtain pure compounds 6a, 6c, 6e, 6i, and 6k in pure form (75–92% yield).

### General procedure for the ultrasonic synthesis of compounds 6a, 6c, 6e, 6i, and 6k

A solution of azazerumbone 1 (5) (1.0 equivalent) in THF (10 mL) was added to the amide 2a or 2c, 2e, 2i, and 2k (1.0 equivalent) followed by NaH (3.0 equivalent) at 0 °C temperature. The mixture was carried out in ultrasonic condition at room temperature and 40 kHz. After completion (2 h), the mixture of the reactions was concentrated and extracted with CH_2_Cl_2_. The organic phase was washed with water and saturated bine. Drying of the organic phase (MgSO_4_), filtration of the drying agent, and evaporation of the solvent *in vacuo* afforded crude compounds. The crude mass obtained was purified by column chromatography on silica gel (Hexane–EtOAc, 90 : 10) to obtain pure compounds 6a, 6c, 6e, 6i, and 6k in pure form (68–89% yield).

#### 2-((3*E*,7*E*,11*E*)-3,7,10,10-Tetramethyl-2-oxoazacyclododeca-3,7,11-trien-1-yl)-*N*-(2-(trifluoromethyl)phenyl)acetamide (6a)

Colourless oil. IR (KBr) *ν*_max_/cm^−1^ 3232; 3088; 2955; 2916; 2846; 1714; 1615; 1583; 1421; 1370; 1209; 1079; 779; 729. ^1^H NMR (600 MHz, CDCl_3_-*d*_1_) *δ* ppm: 8.21 (1H, d, *J* = 7.8 Hz); 8.12 (1H, bs, NH); 7.59 (1H, d, *J* = 7.8 Hz); 7.54 (1H, t, *J* = 7.8 Hz); 7.22 (1H, t, *J* = 7.8 Hz); 6.61 (1H, d, *J* = 14.4 Hz); 5.61 (1H, t, *J* = 7.2 Hz); 5.16 (1H, t, *J* = 6.0 Hz); 4.92 (1H, d, *J* = 14.4 Hz); 4.44 (2H, s); 2.41 (2H, q, *J* = 6.6 Hz); 2.31 (2H, t, *J* = 6.6 Hz); 2.12 (2H, d, *J* = 6.0 Hz); 1.90 (3H, s); 1.60 (3H, s); 1.04 (6H, s). ^13^C NMR (150 MHz, CDCl_3_-*d*_1_) *δ* ppm: 173.4; 167.2; 138.5; 134.8; 134.0; 132.8; 129.4; 128.5; 126.1 (q, *J* = 26.0 Hz); 126.0; 125.1; 124.9 (q, *J* = 285.0 Hz); 124.6; 124.5; 121.1; 49.1; 39.1; 38.6; 35.2; 30.0; 25.2; 15.0; 13.7; 13.6. HR-ESI-MS: found *m*/*z* 435.2252; calcd for C_24_H_30_F_3_N_2_O_2_^+^: 435.2254 [M + H]^+^.

#### *N*-(3-Nitrophenyl)-2-((3*E*,7*E*,11*E*)-3,7,10,10-tetramethyl-2-oxoazacyclododeca-3,7,11-trien-1-yl)acetamide (6c)

Yellow powder, m.p. 75–76 °C. IR (KBr) *ν*_max_/cm^−1^ 3283; 3094; 2955; 2921; 2853; 1712; 1608; 1529; 1431; 1348; 1138; 737; 674. ^1^H NMR (600 MHz, CDCl_3_-*d*_1_) *δ* ppm: 8.95 (1H, bs, NH); 8.37 (1H, t, *J* = 1.8 Hz); 7.91 (1H, d, *J* = 8.4 Hz); 7.79 (1H, d, *J* = 8.4 Hz); 7.42 (1H, t, *J* = 8.4 Hz); 6.52 (1H, d, *J* = 14.4 Hz); 5.61 (1H, t, *J* = 7.2 Hz); 5.14 (1H, t, *J* = 6.6 Hz); 5.08 (1H, d, *J* = 14.4 Hz); 4.38 (2H, s); 2.40 (2H, q, *J* = 6.6 Hz); 2.31 (2H, t, *J* = 6.6 Hz); 2.13 (2H, d, *J* = 6.0 Hz); 1.91 (3H, s); 1.58 (3H, s); 1.09 (6H, s). ^13^C NMR (150 MHz, CDCl_3_-*d*_1_) *δ* ppm: 174.2; 167.1; 148.5; 139.2; 138.9; 133.9; 129.7; 129.6; 128.3; 125.3; 125.1; 122.1; 118.7; 114.5; 50.2; 39.0; 38.5; 35.2; 30.1; 25.1; 15.0; 13.8; 13.7. HR-ESI-MS: found *m*/*z* 412.2228; calcd for C_23_H_30_N_3_O_4_^+^: 412.2231 [M + H]^+^.

#### *N*-(4-Chlorophenyl)-2-((3*E*,7*E*,11*E*)-3,7,10,10-tetramethyl-2-oxoazacyclododeca-3,7,11-trien-1-yl)acetamide (6e)

White powder, m.p. 110–111 °C. IR (KBr) *ν*_max_/cm^−1^ 3256; 3083; 2954; 2920; 2853; 1720; 1600; 1547; 1491; 1348; 1194; 830; 732. ^1^H NMR (600 MHz, CDCl_3_-*d*_1_) *δ* ppm: 8.51 (1H, bs, NH); 7.43 (2H, dd, *J* = 1.8, 8.4 Hz); 7.24 (2H, dd, *J* = 1.8, 8.4 Hz); 6.53 (1H, d, *J* = 15.6 Hz); 5.59 (1H, t, *J* = 7.2 Hz); 5.15 (1H, t, *J* = 6.0 Hz); 5.06 (1H, d, *J* = 15.6 Hz); 4.34 (2H, s); 2.38 (2H, d, *J* = 6.0 Hz); 2.29 (2H, t, *J* = 6.0 Hz); 2.12 (2H, d, *J* = 6.0 Hz); 1.89 (3H, s); 1.57 (3H, s); 1.07 (6H, s). ^13^C NMR (150 MHz, CDCl_3_-*d*_1_) *δ* ppm: 173.9; 166.7; 138.8; 136.4; 133.9; 129.7; 128.9 (2× C); 126.8; 125.1; 121.8; 121.1 (2× C); 120.1; 50.0; 39.1; 38.7; 35.2; 30.1; 25.1; 15.0; 13.7; 13.2. HR-ESI-MS: found *m*/*z* 401.1984; calcd for C_23_H_30_ClN_2_O_2_^+^: 401.1990 [M + H]^+^.

#### *N*-(4-Chloro-2-methylphenyl)-2-((3*E*,7*E*,11*E*)-3,7,10,10-tetramethyl-2-oxoazacyclododeca-3,7,11-trien-1-yl)acetamide (6i)

White powder, m.p. 122–123 °C. IR (KBr) *ν*_max_/cm^−1^ 3264; 3092; 2954; 2919; 2851; 1729; 1600; 1529; 1451; 1348; 1178; 820; 733. ^1^H NMR (600 MHz, CDCl_3_-*d*_1_) *δ* ppm: 8.24 (1H, bs, NH); 7.89 (1H, d, *J* = 8.4 Hz); 7.16 (1H, d, *J* = 8.4 Hz); 7.14 (1H, s); 6.55 (1H, d, *J* = 14.4 Hz); 5.55 (1H, t, *J* = 7.8 Hz); 5.13 (1H, t, *J* = 6.6 Hz); 5.08 (1H, d, *J* = 14.4 Hz); 4.37 (2H, s); 2.38 (2H, q, *J* = 6.6 Hz); 2.29 (2H, t, *J* = 6.0 Hz); 2.11 (2H, t, *J* = 6.0 Hz); 1.89 (3H, s); 1.58 (3H, s); 1.08 (6H, s). ^13^C NMR (150 MHz, CDCl_3_-*d*_1_) *δ* ppm: 173.8; 167.0; 138.6; 134.5; 133.9; 130.1; 130.1; 129.6; 129.6; 128.6; 126.6; 125.2; 123.2; 121.8; 50.1; 39.0; 38.6; 35.3; 30.1; 25.1; 17.7; 15.1; 14.1; 13.7. HR-ESI-MS: found *m*/*z* 415.2139; calcd for C_24_H_32_ClN_2_O_2_^+^: 415.2147 [M + H]^+^.

#### (3*E*,7*E*,11*E*)-3,7,10,10-Tetramethyl-1-(2-morpholino-2-oxoethyl)azacyclododeca-3,7,11-trien-2-one (6k)

Colourless oil. IR (KBr) *ν*_max_/cm^−1^ 3089; 2970; 2925; 2856; 1644; 1441; 1271; 1242; 1112; 845. ^1^H NMR (600 MHz, CDCl_3_-*d*_1_) *δ* ppm: 6.49 (1H, d, *J* = 15.0 Hz); 5.66 (1H, t, *J* = 7.8 Hz); 5.13 (1H, t, *J* = 6.0 Hz); 4.69 (1H, d, *J* = 15.0 Hz); 4.38 (2H, s); 3.68–3.70 (2H, m); 3.58–3.61 (2H, m); 3.51–3.55 (2H, m); 2.36 (2H, q, *J* = 6.6 Hz); 2.27 (2H, t, *J* = 6.6 Hz); 2.12 (2H, d, *J* = 6.0 Hz); 1.85 (3H, s); 1.57 (3H, s); 1.06 (6H, s). ^13^C NMR (150 MHz, CDCl_3_-*d*_1_) *δ* ppm: 172.9; 166.0; 137.7; 133.9; 130.5; 128.9; 125.0; 119.8; 65.5 (2× C); 45.4; 42.0 (2× C); 39.0; 38.6; 35.1; 30.0; 25.0; 15.1; 14.1; 13.6. HR-ESI-MS: found *m*/*z* 361.2565; calcd for C_21_H_33_N_2_O_3_^+^: 361.2486 [M + H]^+^.

### Cell culture and cell viability assay

The synthesized compounds (4a–l, 6a, 6c, 6e, 6i and 6k) were evaluated for their cytotoxicity against four human cancer cell lines, including hepatoma carcinoma cell line (HepG2), human lung carcinoma (A549), human acute leukemia (HL-60), human gastric carcinoma (AGS) obtained from the American Type Culture Collection (USA) ATCC and used for cytotoxic evaluation. The human cancer cell lines were cultured in RPMI 1640 medium supplemented with 10% fetal bovine serum (FBS), 100 U per mL penicillin, and 100 μg per mL streptomycin at 37 °C in a humidified environment containing 95% air and 5% CO_2_. Cells in the exponential growth phase were utilized throughout the experiments. The antiproliferative effects of the test compounds on cancer cell viability were assessed using the 3-[4,5-dimethylthiazol-2-yl]-2,5-diphenyl-tetrazolium bromide (MTT) assay, which measures cellular metabolic activity. In brief, human cancer cell lines were seeded at a density of 1 × 10^5^ cells per mL and treated for 72 hours with serial dilutions of the test compounds (dissolved in DMSO) at concentrations of 0.125, 0.5, 2.0, 8.0, 32.0, and 128.0 μg mL^−1^. Following incubation, 50 μL of MTT solution (2 mg mL^−1^) was added to each well, and the cells were further incubated at 37 °C for 4 hours. The plates were subsequently centrifuged at 1000 rpm for 10 minutes at room temperature, and the supernatant was carefully aspirated. The resulting formazan crystals were dissolved by adding 150 μL of dimethyl sulfoxide (DMSO) to each well. Absorbance was immediately recorded at 540 nm using a TECAN GENIOUS microplate reader. All experiments were conducted in triplicate, and the mean absorbance values were calculated. The results were expressed as the percentage of growth inhibition, determined by the reduction in absorbance relative to untreated control cells. A dose–response curve was generated, and the half-maximal inhibitory concentration (IC_50_) was calculated for each compound and each cell line.

### Molecular docking study

Molecular docking simulations were performed to evaluate the binding ability of compound 4a–l against common cytotoxic targets according to our chemical design. The crystallographic structure of the EGFR tyrosine kinase domain in complex with erlotinib (PDB ID: 4HJO, resolution 2.75 Å) was obtained from the Protein Data Bank (https://www.rcsb.org/structure/4HJO) and utilized as the target for docking simulations. Preparation of the protein crystal structure involved the removal of all water molecules and non-receptor ligands. Polar hydrogens and Kollman charges were added using AutoDock Tools, and the resulting structure was saved in PDBQT format. Ligand structures were generated using Chem3D, followed by optimization and conversion to the PDBQT format. A grid box with dimensions of 15 × 15 × 15 Å and a grid spacing of 0.375 Å was employed to define the docking area. Binding energy is calculated based on the AutoDock scoring function embedded in the automated molecular docking software package. The energetic terms include contributions from intermolecular interactions, including van der Waals forces, electrostatic interactions, hydrogen bonding, desolvation effects, and loss of ligand torsional entropy upon binding. For each ligand, the top nine poses were generated after docking. A more negative binding energy and zero RMSD were selected as the most favorable docking pose and interaction. To validate the docking procedure, erlotinib was redocked into the active site of the receptor. The redocked structure showed a superposition with the co-crystallized ligand, yielding an RMSD of 1.502 Å, confirming the reliability of the docking algorithm when compared to the experimental crystallographic structure. Furthermore, the redocked erlotinib exhibited similar binding interactions to the co-crystallized ligand, forming two conventional hydrogen bonds with Met769 (3.08 Å) and Cys773 (3.15 Å), as well as hydrophobic interactions with residues Val702, Thr830, Lys721, Leu764, Ala719, Leu694, Gln767, Leu820, Pro770, and Phe771.

### Prediction of physicochemical and ADMET (absorption, distribution, metabolism, excretion, and toxicity) properties

Three potential lead compounds (4c, 4g and 4i) were assessed for their ADMET properties (Absorption, Distribution, Metabolism, Excretion, and Toxicity) using SwissADME (Daina A, Michielin O, Zoete V. SwissADME: a free web tool for evaluating pharmacokinetics, drug-likeness, and medicinal chemistry friendliness of small molecules),^34^ ADMETlab 3.0 (https://admetlab3.scbdd.com/), and pkCSM (Pires DEV, Blundell TL, Ascher DB). pkCSM: predicting small-molecule pharmacokinetic and toxicity properties using graph-based signatures^35^ (Table 4).

## Conclusions

In conclusion, a series of seventeen novel zerumbone-secondary amide hybrids was successfully designed and synthesized with high yields using both conventional and ultrasonic methods. Reactions conducted under ultrasonic conditions required significantly shorter reaction times than those performed without ultrasound while maintaining comparable product yields. Most synthesized derivatives exhibited significant cytotoxic activity against four human tumor cell lines. Notably, zerumbone-secondary amide derivatives derived from azazerumbone 2 (3) demonstrated greater cytotoxic potency than those from azazerumbone 1 (5). Moreover, the conversion of azazerumbone 1 into zerumbone-secondary amides markedly enhanced its cytotoxic activity, suggesting that the incorporation of secondary amide groups contributes to increased anticancer potency. Among the tested derivatives, three compounds (4c, 4g, and 4i) displayed the most potent cytotoxic effects across all tested cell lines, with IC_50_ values ranging from 0.81 ± 0.04 to 4.14 ± 0.44 μg mL^−1^, comparable to those of zerumbone and ellipticine. Molecular docking studies have demonstrated that zerumbone-amide derivatives exhibit a strong binding affinity to EGFR tyrosine kinase, which correlates with their observed cytotoxic activity. This finding also reinforces that the incorporation of secondary amide groups into the zerumbone framework has contributed to the enhancement of the cytotoxic activity of these compounds. Furthermore, the three most promising compounds (4c, 4g, and 4i) exhibited favorable pharmacokinetic properties, further supporting their potential as lead candidates for anticancer drug development.

## Data availability

The datasets supporting this article have been uploaded as part of the ESI.†

## Author contributions

Pham The Chinh: writing – review & editing, writing – original draft, visualization, supervision, project administration, methodology, investigation, formal analysis, data curation, conceptualization. Pham Thi Tham: writing – original draft, investigation. Vu Thi Lien: investigation. Dao Thi Nhung: software. Le Thi Thuy Loan: investigation. Vu Thi Thu Le: investigation. Vu Tuan Kien: investigation. Cao Thanh Hai: investigation. Phan Thanh Phuong: software.

## Conflicts of interest

The author(s) declared no potential conflicts of interest with respect to the research, authorship, and/or publication of this article.

## Supplementary Material

RA-015-D5RA01215E-s001
