# Supplementary material for: Novel zerumbone-secondary amide hybrids: ultrasonic synthesis, cytotoxic evaluation, molecular docking and in silico ADMET studies
Source: RSC Adv. 2025 Mar 25;15(12):8986–98. doi: 10.1039/d5ra01215e (PMC11934104; doi:10.1039/d5ra01215e)
Supplement: RA-015-D5RA01215E-s001 [file RA-015-D5RA01215E-s001.pdf]

Supplementary Information (SI) for RSC Advances.
This journal is © The Royal Society of Chemistry 2025

# Novel zerumbone-secondary amide hybrids: ultrasonic synthesis, cytotoxic evaluation, molecular docking and *in silico* ADMET studies

Pham The Chinh[a]*, Pham Thi Tham[b]*[1], Vu Thi Lien[a]*, Dao Thi Nhung[c], Le Thi Thuy Loan[a,d], Vu Thi Thu Le[e], Vu Tuan Kien[a], Cao Thanh Hai[a], Phan Thanh Phuong[a]

**General**: All reactions were performed in the appropriate oven-dried glass apparatus and under nitrogen atmosphere. Unless otherwise stated, solvents and chemicals were obtained from commercial sources and used without further purification. Column chromatography was performed using silica gel (60Å, particle size 40-60 µm). Melting points were determined using a Buchi Melting Point B-545 and were uncorrected. Infrared (IR) analysis was conducted with a PerkinElmer Spectrum Two spectrometer using KBr pellets. NMR spectra were recorded on a Bruker Advance I (600 MHz). Chemical shifts (*δ*) are given in parts per million (ppm) and coupling constants (*J*) in hertz (Hz). High resolution mass spectrometry analysis (HRMS) was recorded on a SCIEX X500 QTOF instrument.

[a]Thai Nguyen University of Sciences - TNU, Tan Thinh, 24000 Thai Nguyen, Vietnam
[b]Hanoi University of Industry, Cau Dien, Bac Tu Liem, Hanoi, Vietnam.
[c]VNU University of Science, Vietnam National University, Hanoi, 334 Nguyen Trai Street, Thanh Xuan, Ha Noi, Vietnam
[d]Tay Nguyen University, Le Duan, Buon Ma Thuat, Dak Lak, Vietnam
[e] Thai Nguyen University of Agriculture and Forestry -TNU, Quyet Thang, 24000 Thai Nguyen, Vietnam
[1] Corresponding author. Tel.: +84 988113933; e-mail: chinhpt@tnus.edu.vn (P.T.Chinh); phamthitham85@haui.edu.vn (P.T. Tham); vuthilien203@gmail.com(V.T. Lien)

# Content


**Figure S1**. $^{1}$H-NMR spectrum of compound **2a** 3s

**Figure S2**. $^{13}$C-NMR and HRMS spectra of compound **2a** 4s

**Figure S3**. $^{1}$H-NMR and $^{13}$C-NMR spectra of compound **2b** 5s

**Figure S4**. $^{1}$H-NMR and $^{13}$C-NMR spectra of compound **2c** 6s

**Figure S5**. $^{1}$H-NMR and $^{13}$C-NMR spectra of compound **2d** 7s

**Figure S6**. HRMS spectrum of compound **2d** 8s

**Figure S7**. $^{1}$H-NMR and $^{13}$C-NMR spectra of compound **2e** 9s

**Figure S8**. HRMS spectrum of compound **2e** 10s

**Figure S9**. $^{1}$H-NMR and $^{13}$C-NMR spectra of compound **2f** 11s

**Figure S10**. $^{1}$H-NMR and $^{13}$C-NMR spectra of compound **2g** 12s

**Figure S11**. $^{1}$H-NMR and $^{13}$C-NMR spectra of compound **2h** 13s

**Figure S12**. HRMS spectrum of compound **2h** 14s

**Figure S13**. $^{1}$H-NMR and $^{13}$C-NMR spectra of compound **2i** 15s

**Figure S14**. HRMS spectrum of compound **2i** 16s

**Figure S15**. $^{1}$H-NMR and $^{13}$C-NMR spectra of compound **2j** 17s

**Figure S16**. $^{1}$H-NMR and $^{13}$C-NMR spectra of compound **2k** 18s

**Figure S17**. HRMS spectrum of compound **2k** 19s

**Figure S18**. $^{1}$H-NMR and $^{13}$C-NMR spectra of compound **2l** 20s

**Figure S19**. $^{1}$H-NMR spectrum of compound **4a** 21s

**Figure S20**. $^{13}$C-NMR and HRMS spectra of compound **4a** 22s

**Figure S21**. HSQC and HMBC spectra of compound **4a** 23s

**Figure S22**. $^{1}$H NMR and $^{13}$C NMR spectra of compound **4b** 24s

**Figure S23**. $^{1}$H NMR spectrum of compound **4c** 25s

**Figure S24**. $^{13}$C NMR and HRMS spectra of compound **4c** 26s

**Figure S25**. $^{1}$H NMR spectrum of compound **4d** 27s

**Figure S26**. $^{13}$C NMR and HRMS spectra of compound **4d** 28s

**Figure S27**. $^{1}$H NMR spectrum of compound **4e** 29s

**Figure S28**. $^{13}$C NMR and HRMS spectra of compound **4e** 30s

**Figure S29**. $^{1}$H NMR spectrum of compound **4f** 31s

**Figure S30**. $^{13}$C NMR and HRMS spectra of compound **4f** 32s

**Figure S31**. $^{1}$H NMR spectrum of compound **4g** 33s

**Figure S32**. $^{13}$C NMR and HRMS spectra of compound **4g** 34s

**Figure S33**. $^{1}$H NMR spectrum of compound **4h** 35s

**Figure S34**. $^{13}$C NMR and HRMS spectra of compound **4h** 36s

**Figure S35**. $^{1}$H NMR spectrum of compound **4i** 37s

**Figure S36**. $^{13}$C NMR and HRMS spectra of compound **4i** 38s

**Figure S37**. $^{1}$H NMR spectrum of compound **4j** 39s

**Figure S38**. $^{13}$C NMR and HRMS spectra of compound **4j** 40s

**Figure S39**. $^{1}$H NMR spectrum of compound **4k** 41s

**Figure S40**. $^{13}$C NMR and HRMS spectra of compound **4k** 42s

**Figure S41**. $^{1}$H NMR and $^{13}$C NMR spectra of compound **4l** 43s

**Figure S42**. $^{1}$H NMR spectrum of compound **6a** 44s

**Figure S43**. $^{13}$C NMR and HRMS spectra of compound **6a** 45s

**Figure S44**. $^{1}$H NMR spectrum of compound **6c** 46s

**Figure S45**. $^{13}$C NMR and HRMS spectra of compound **6c** 47s

**Figure S46**. $^{1}$H NMR spectrum of compound **6e** 48s

**Figure S47**. $^{13}$C NMR and HRMS spectra of compound **6e** 49s

**Figure S48**. $^{1}$H NMR spectrum of compound **6i** 50s

**Figure S49**. $^{13}$C NMR and HRMS spectra of compound **6i** 51s

**Figure S50**. $^{1}$H NMR spectrum of compound **6k** 52s

**Figure S51**. $^{13}$C NMR and HRMS spectra of compound **6k** 53s

**Fig 1** Binding interactions of ligands into the active site of receptor: 3D (left) and 2D (right) 54s

**Table 3** Binding energy and bond interactions 55s

Figure 2. Radar diagram illustrating the physicochemical characteristics of compounds 4c, 4g, and 4i 56s

**Table 4.** Prediction of ADMET properties of the compounds using pkCSM and Swiss ADME 57s

HT91-CDCl3-1H

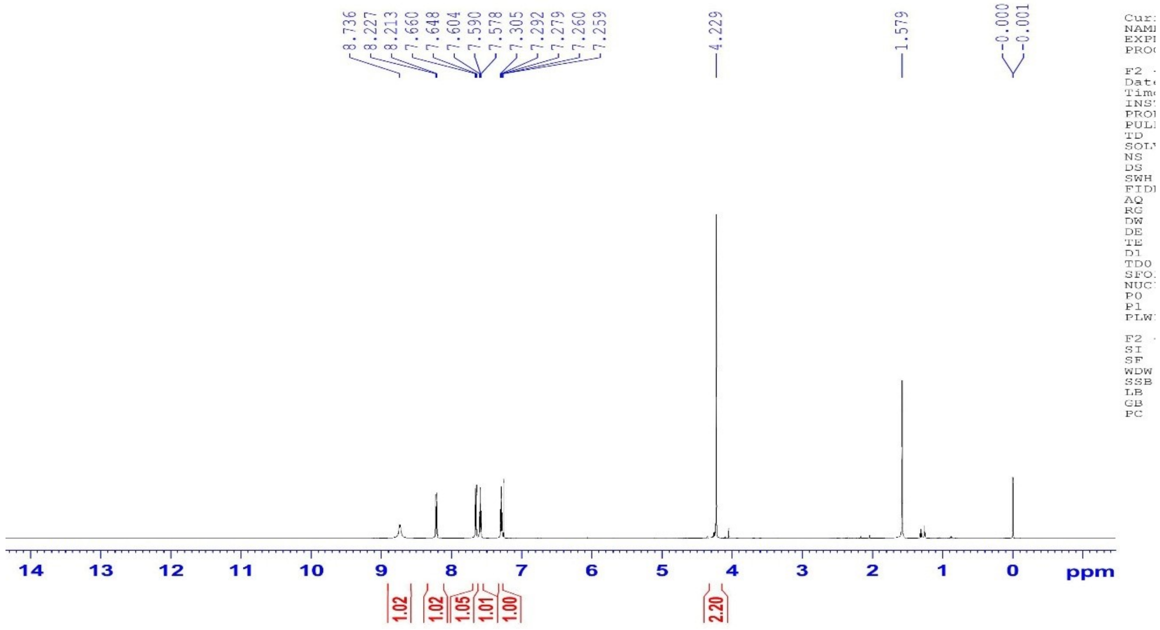

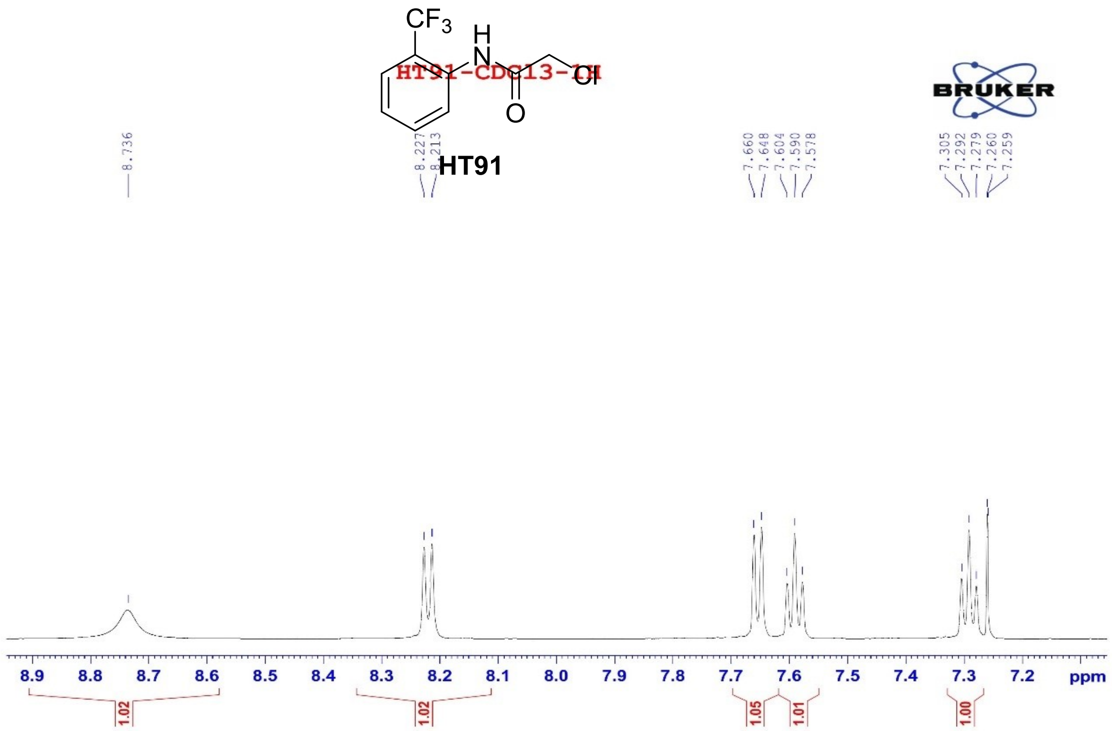


**Figure S1**. $^{1}$H-NMR spectrum of compound **2a**

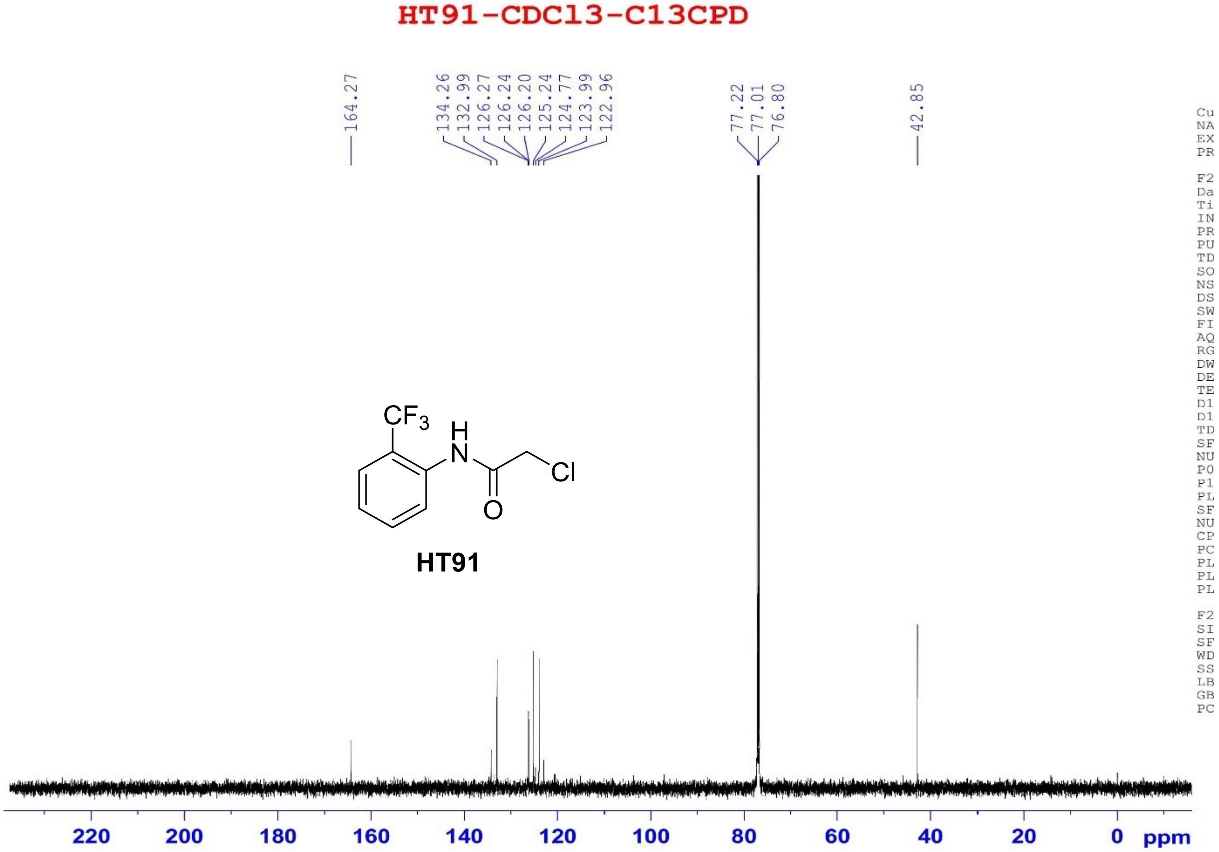


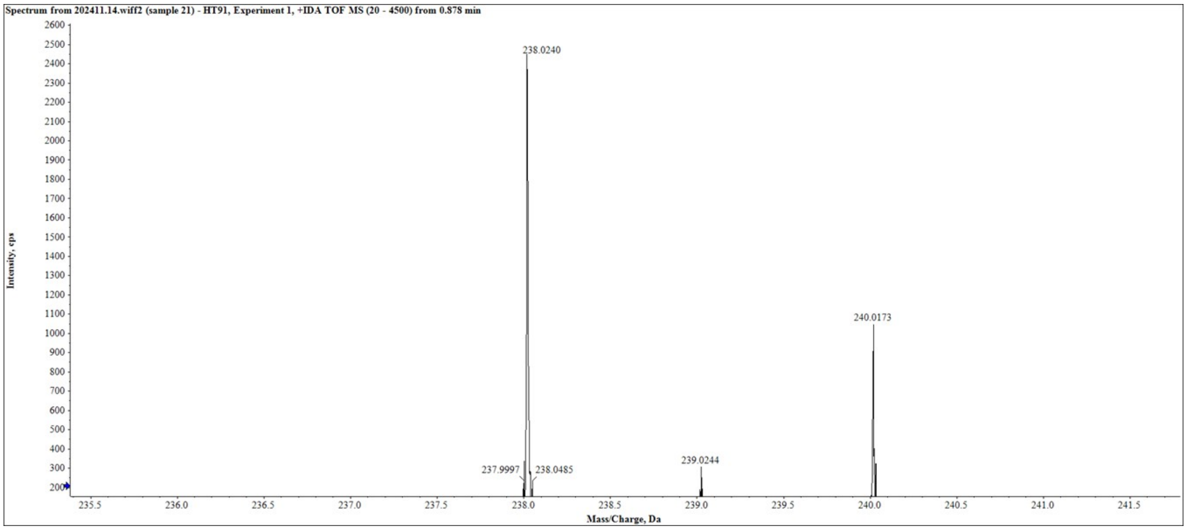


| Hit | Formula | m/z | RDB | ppm | MS Rank | MSMS ppm | MSMS Rank | Found |
|---|---|---|---|---|---|---|---|---|
| 1 | C9H7ClF3NO | 238.02410 | 5.0 | -0.3 | 1 | | | NA/NA |

**Figure S2**. $^{13}$C-NMR and HRMS spectra of compound **2a**

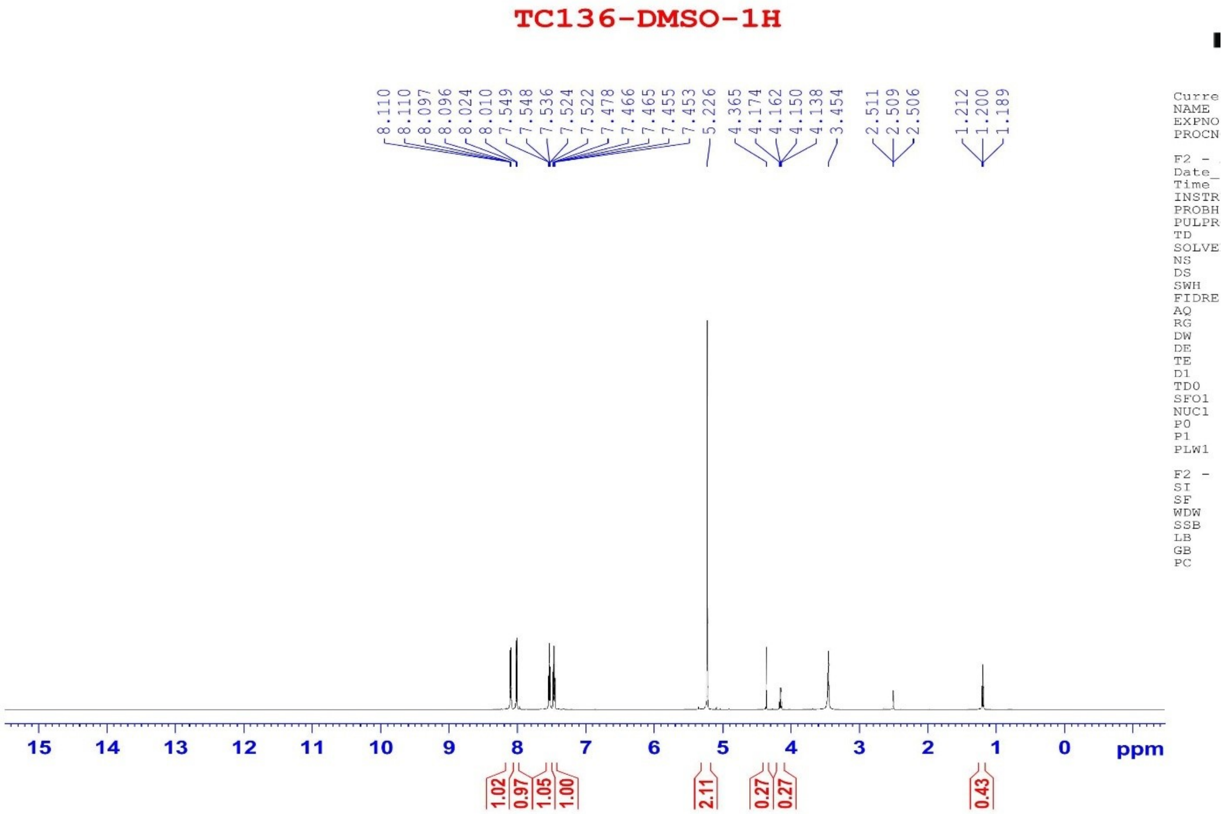


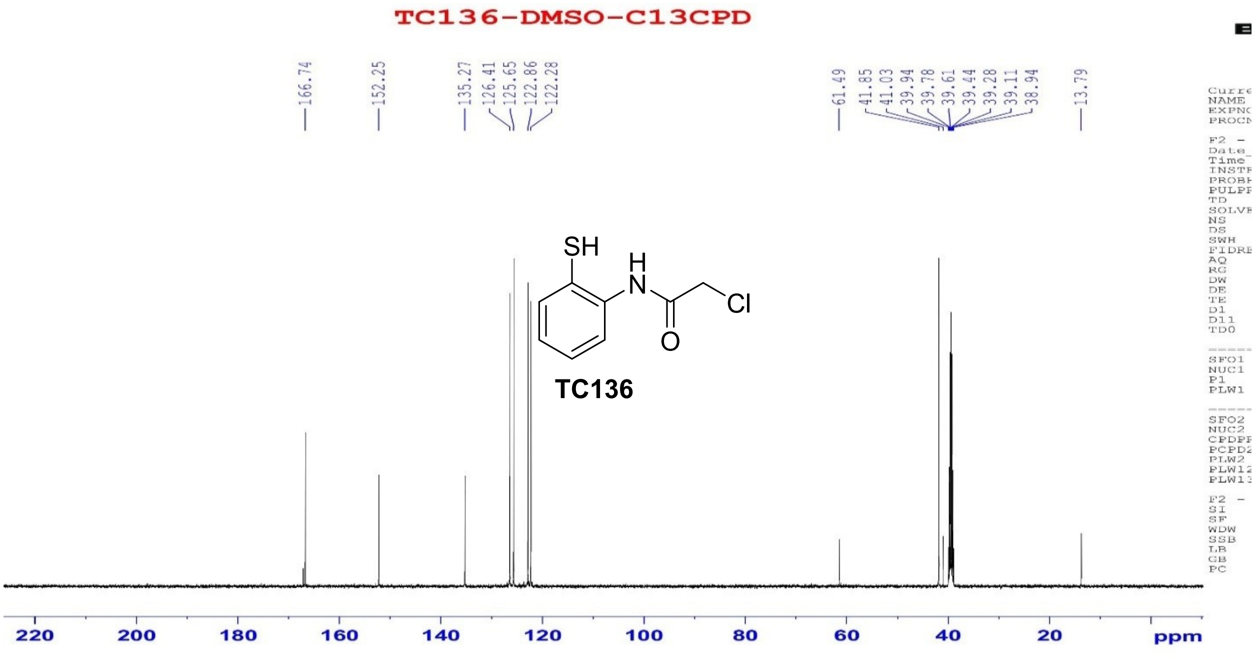


**Figure S3**. $^{1}$H-NMR and $^{13}$C-NMR spectra of compound **2b**

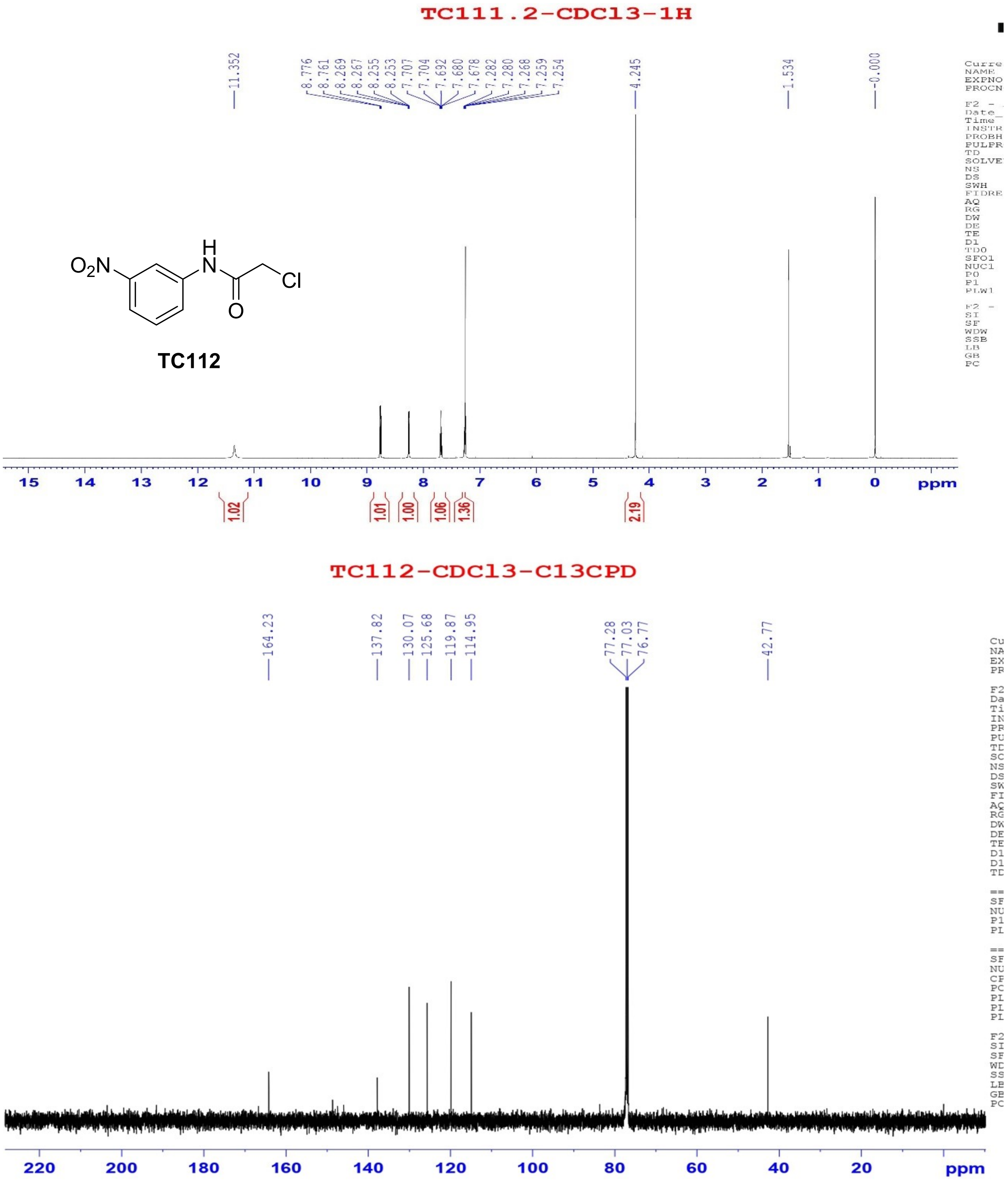


**Figure S4**. $^{1}$H-NMR and $^{13}$C-NMR spectra of compound **2c**

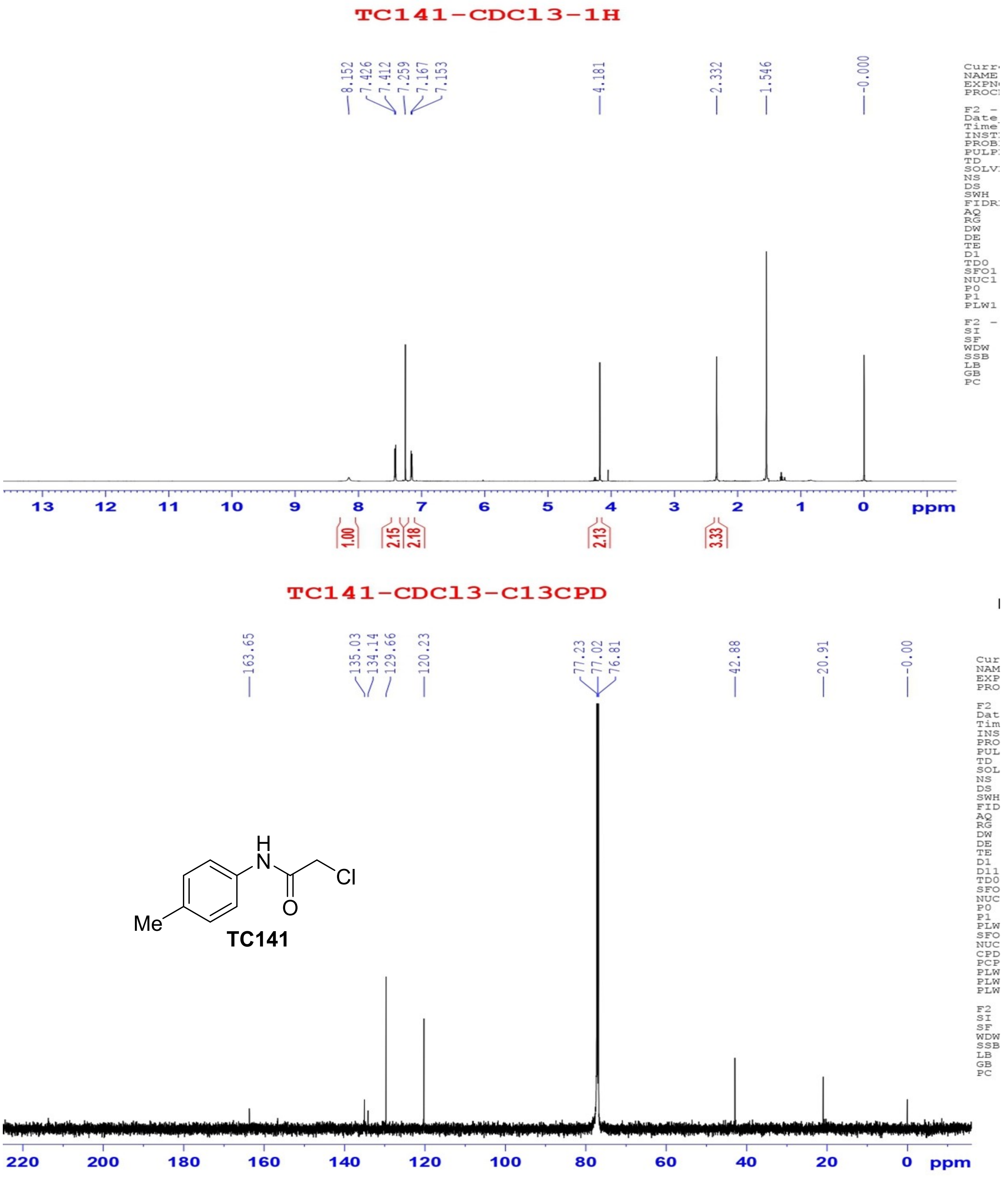


**Figure S5**. $^{1}$H-NMR and $^{13}$C-NMR spectra of compound **2d**

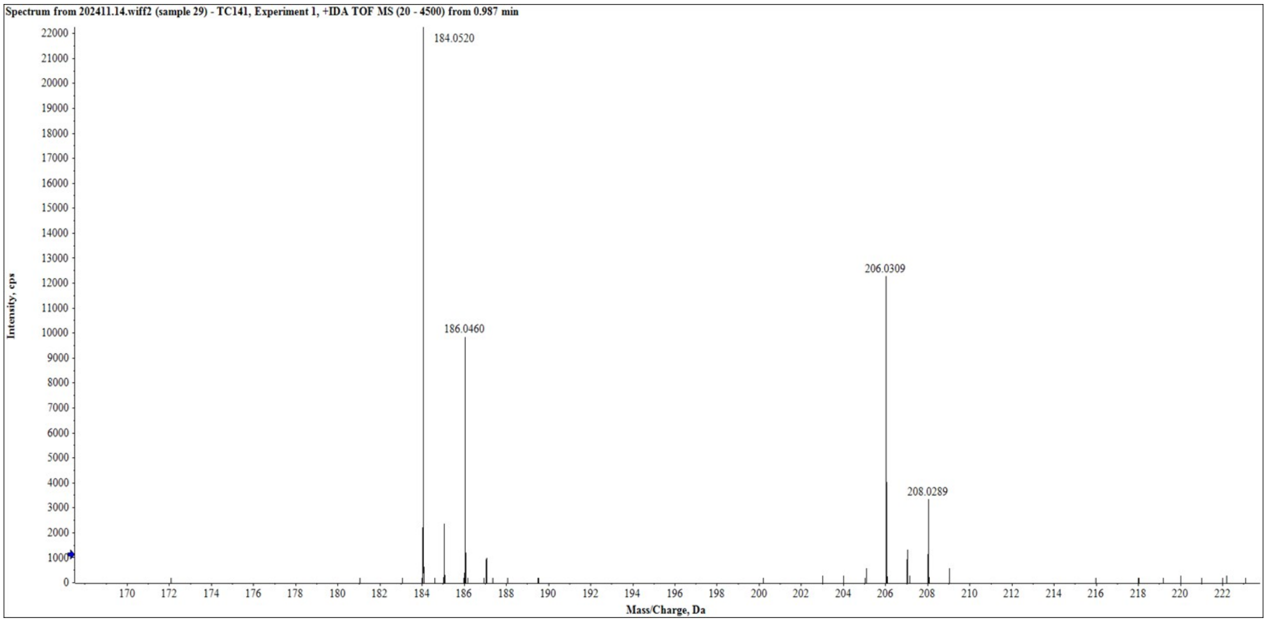


| Hit | Formula | m/z | RDB | ppm | MS Rank | MSMS ppm | MSMS Rank | Found |
|---|---|---|---|---|---|---|---|---|
| 1 | C9H10ClNO | 184.05237 | 5.0 | -2.0 | 1 | | | NA/NA |

**Figure S6**. HRMS spectrum of compound **2d**

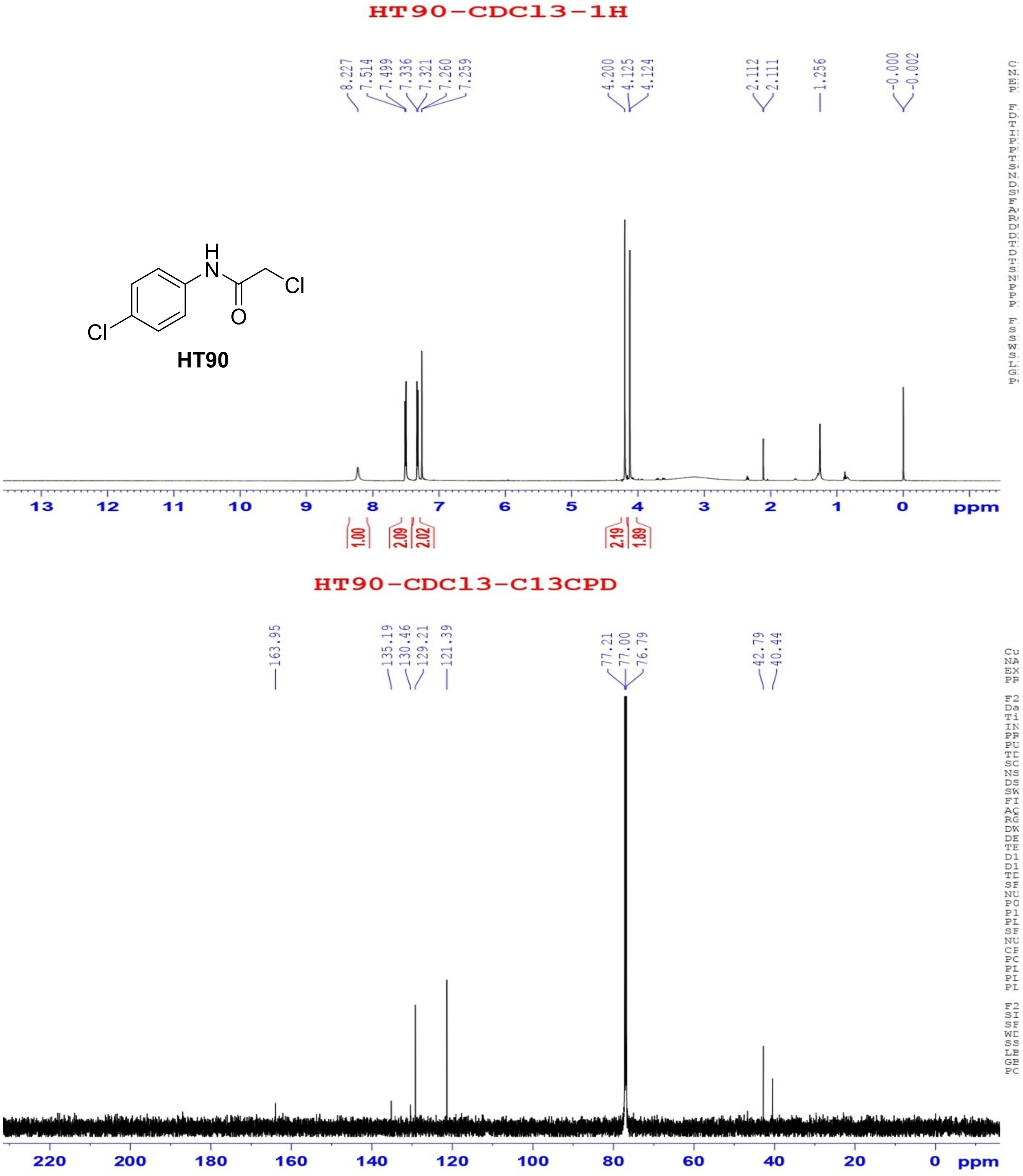


**Figure S7**. $^{1}$H-NMR and $^{13}$C-NMR spectra of compound **2e**

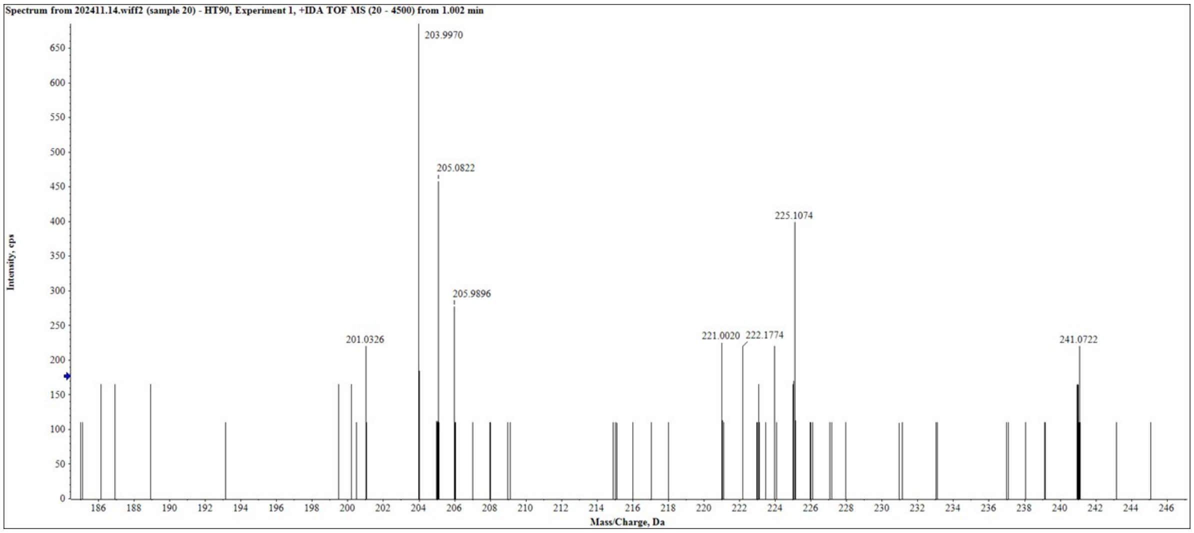


| Hit | Formula | m/z | RDB | ppm | MS Rank | MSMS ppm | MSMS Rank | Found |
|---|---|---|---|---|---|---|---|---|
| 1 | C8H7Cl2NO | 203.99775 | 5.0 | -22.4 | 1 | | | NA/NA |

**Figure S8**. HRMS spectrum of compound **2e**

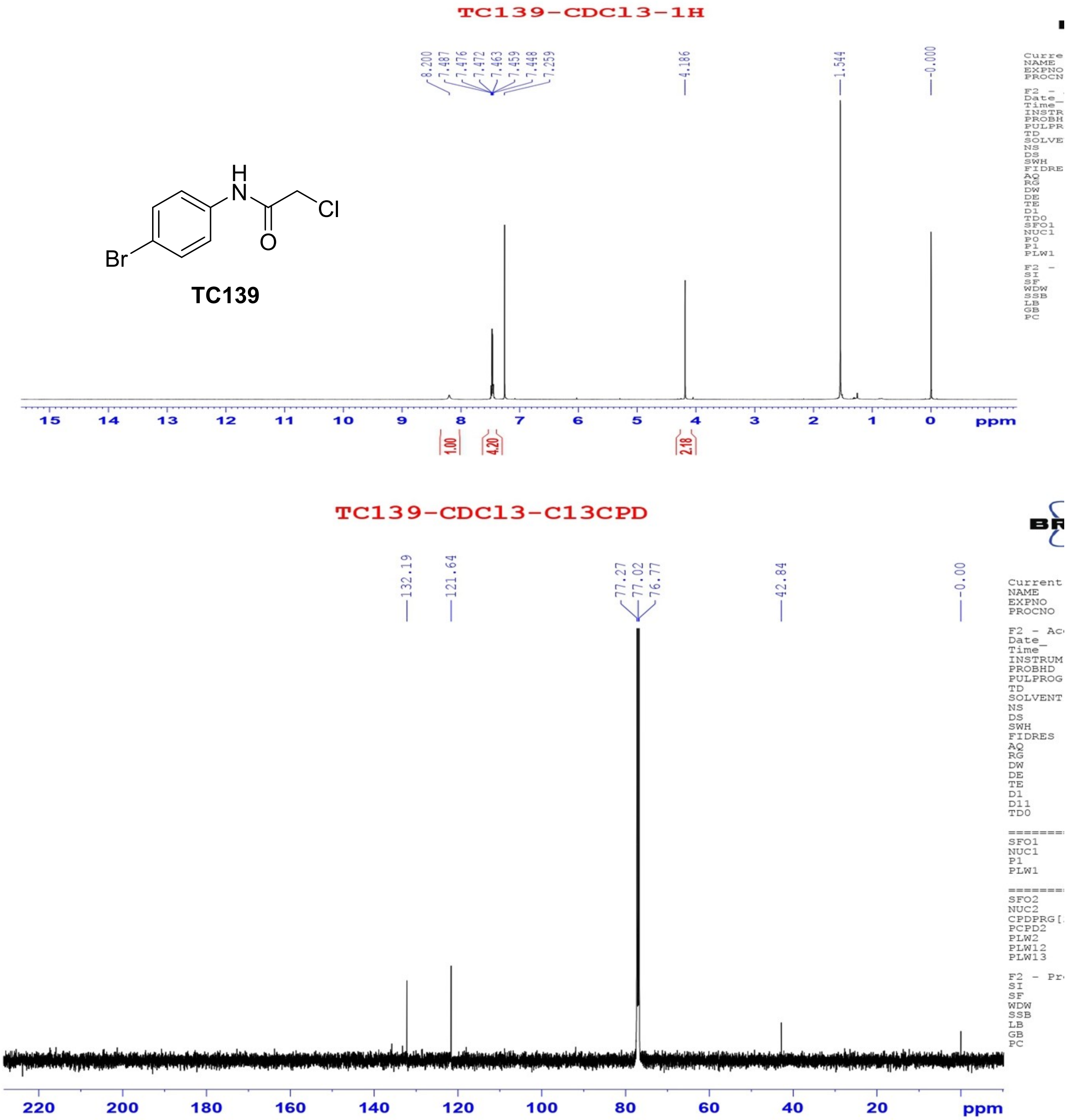


**Figure S9**. $^1$H-NMR and $^{13}$C-NMR spectra of compound **2f**

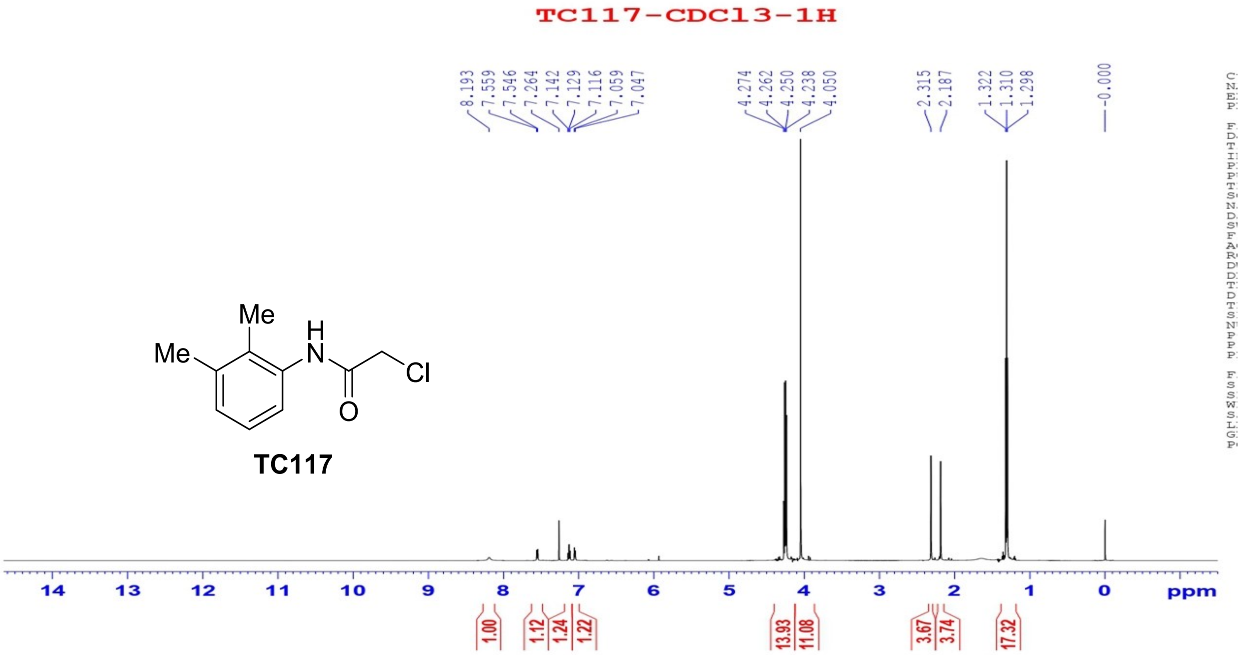


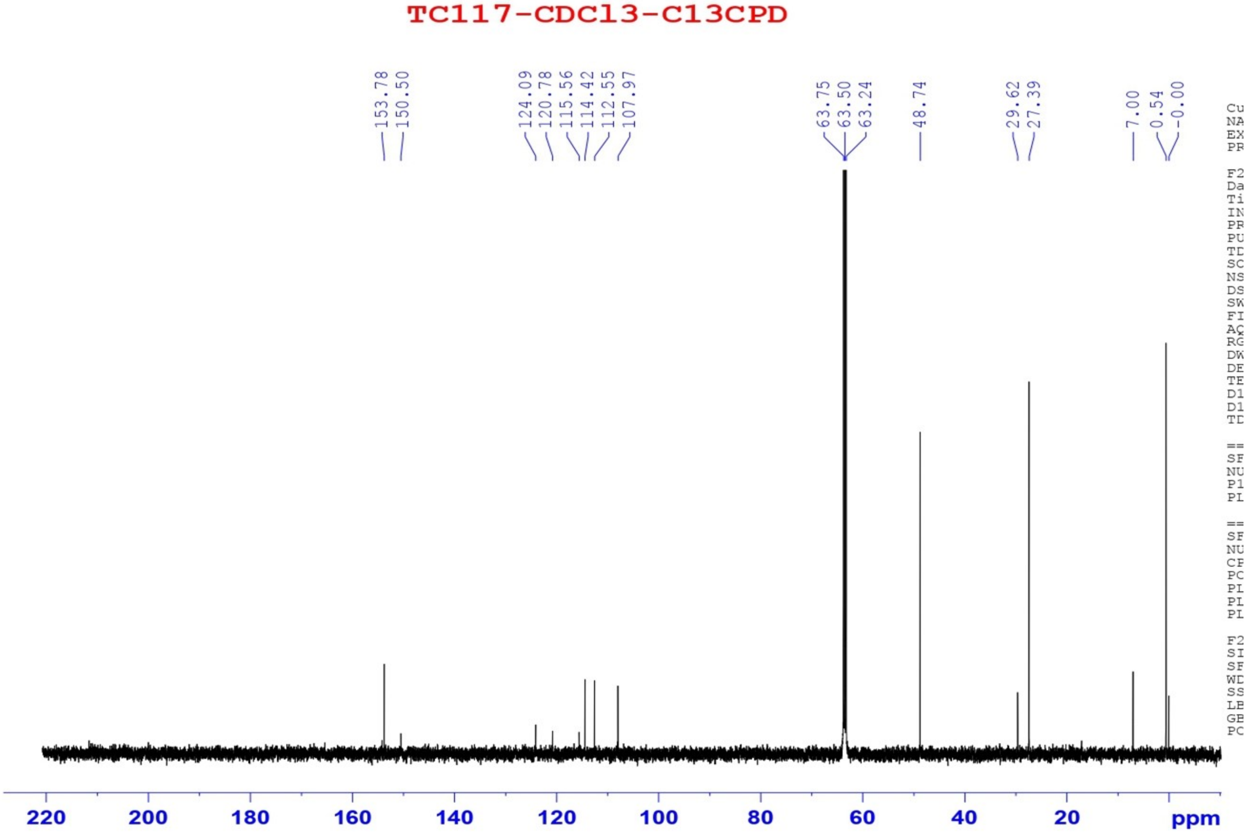


**Figure S10**. $^{1}$H-NMR and $^{13}$C-NMR spectra of compound **2g**

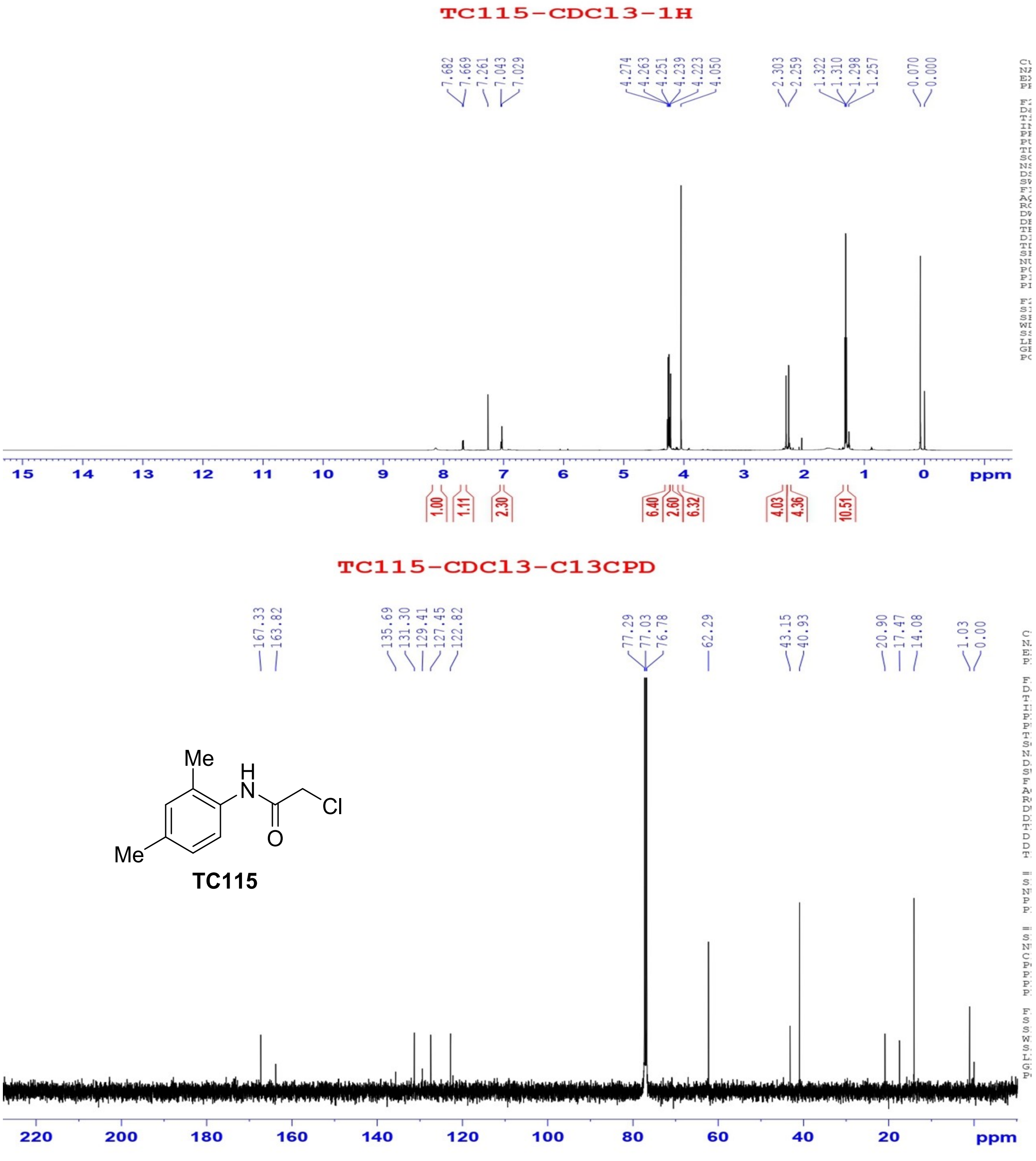


**Figure S11**. $^{1}$H-NMR and $^{13}$C-NMR spectra of compound **2h**

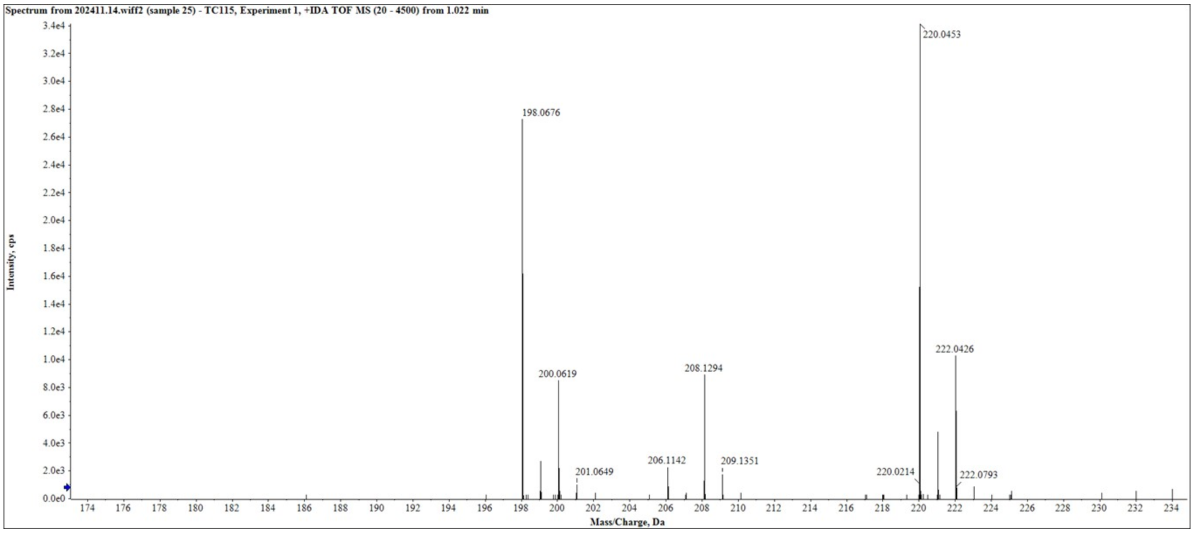


| Hit | Formula | m/z | RDB | ppm | MS Rank | MSMS ppm | MSMS Rank | Found |
|---|---|---|---|---|---|---|---|---|
| 1 | C10H12ClNO | 198.06802 | 5.0 | -2.1 | 1 | | | NA/NA |

**Figure S12**. HRMS spectrum of compound **2h**

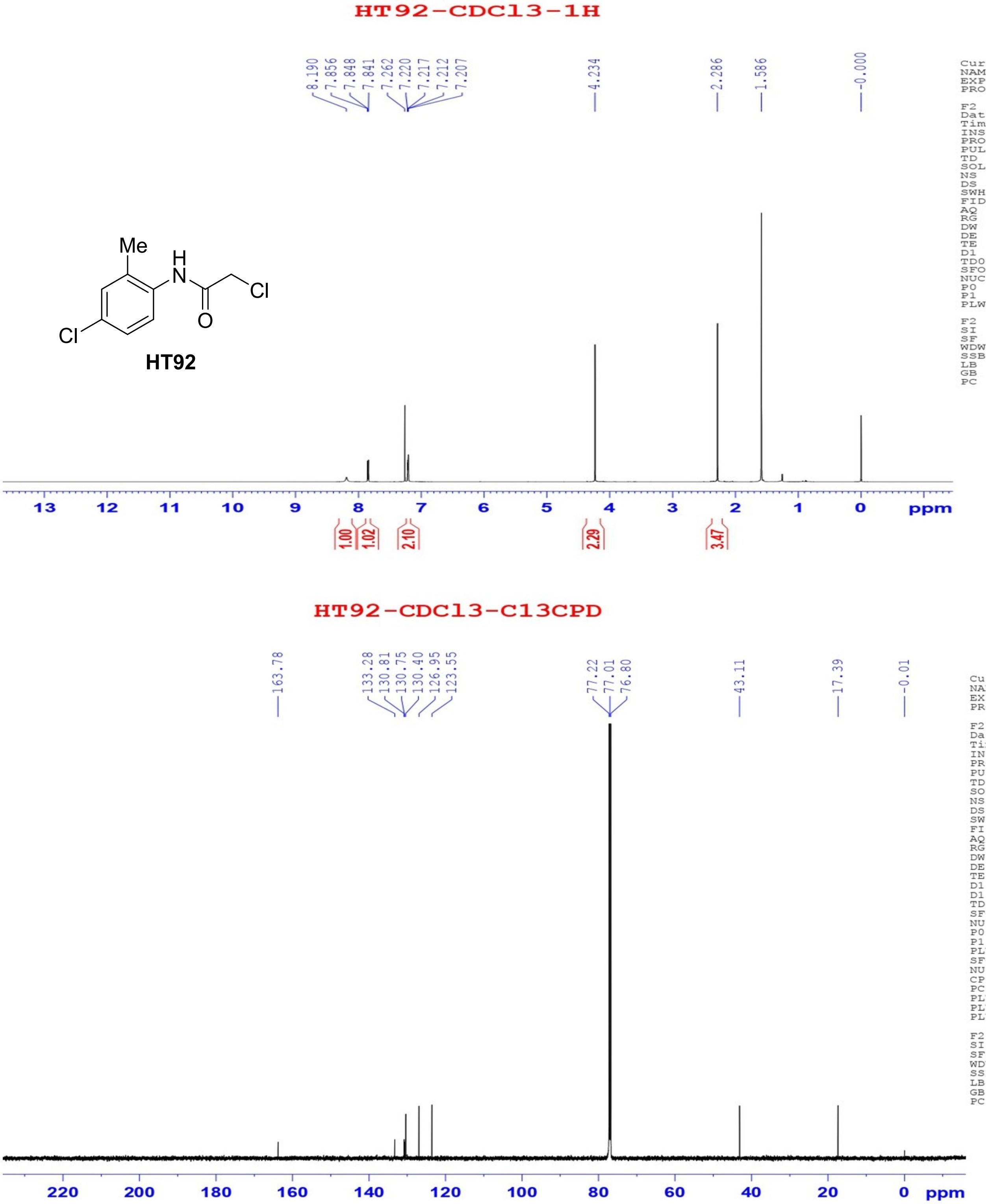


**Figure S13**. $^{1}$H-NMR and $^{13}$C-NMR spectra of compound **2i**

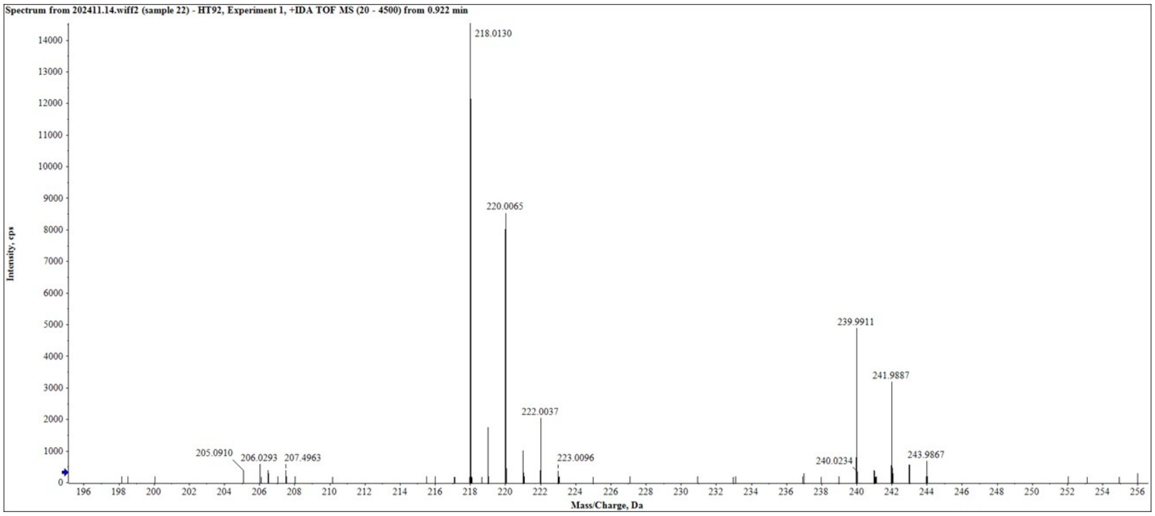


| Hit | Formula | m/z | RDB | ppm | MS Rank | MSMS ppm | MSMS Rank | Found |
|---|---|---|---|---|---|---|---|---|
| 1 | C9H9Cl2NO | 218.01340 | 5.0 | -1.8 | 1 | | | NA/NA |

**Figure S14**. HRMS spectrum of compound **2i**

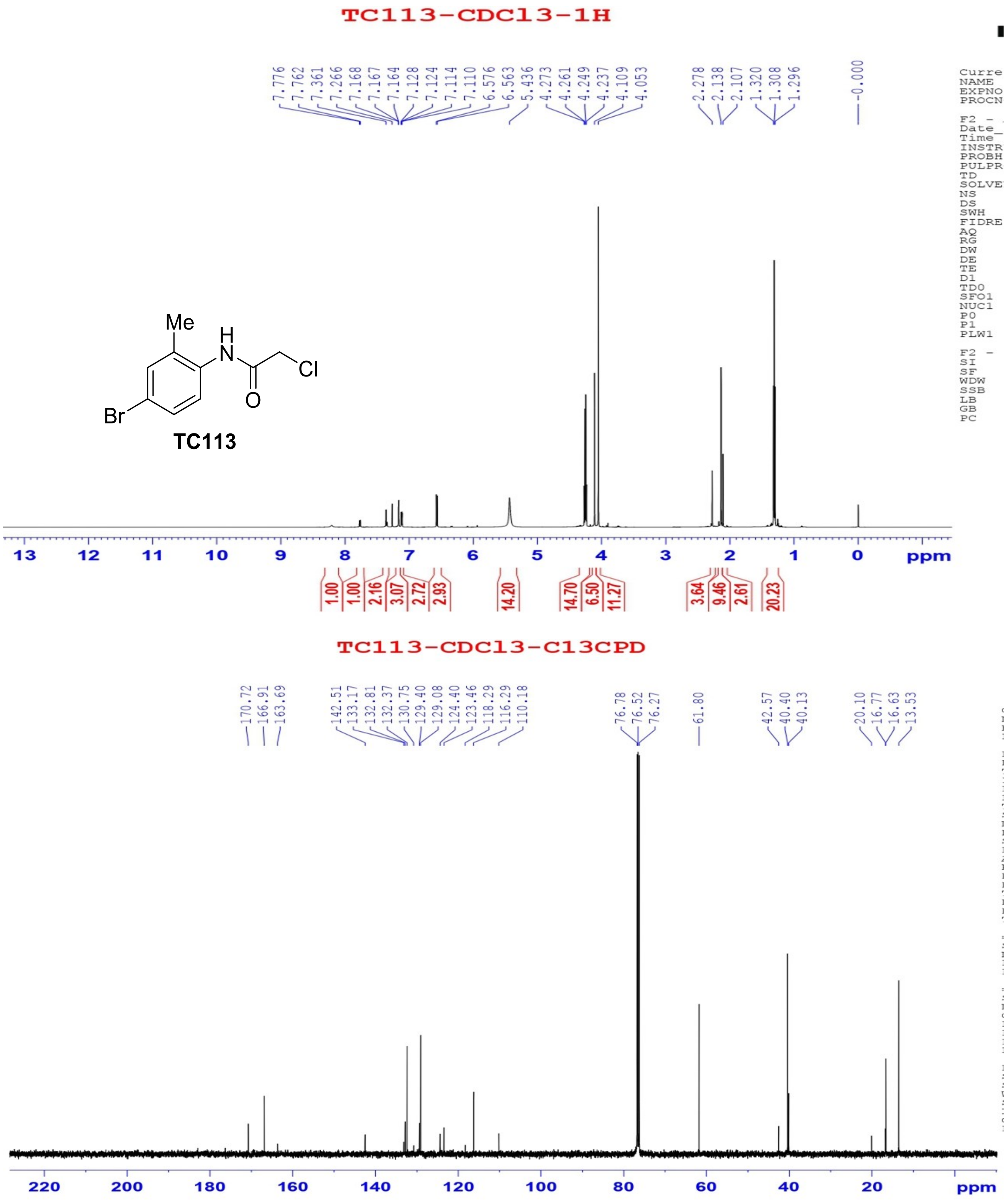


**Figure S15**. $^{1}$H-NMR and $^{13}$C-NMR spectra of compound **2j**

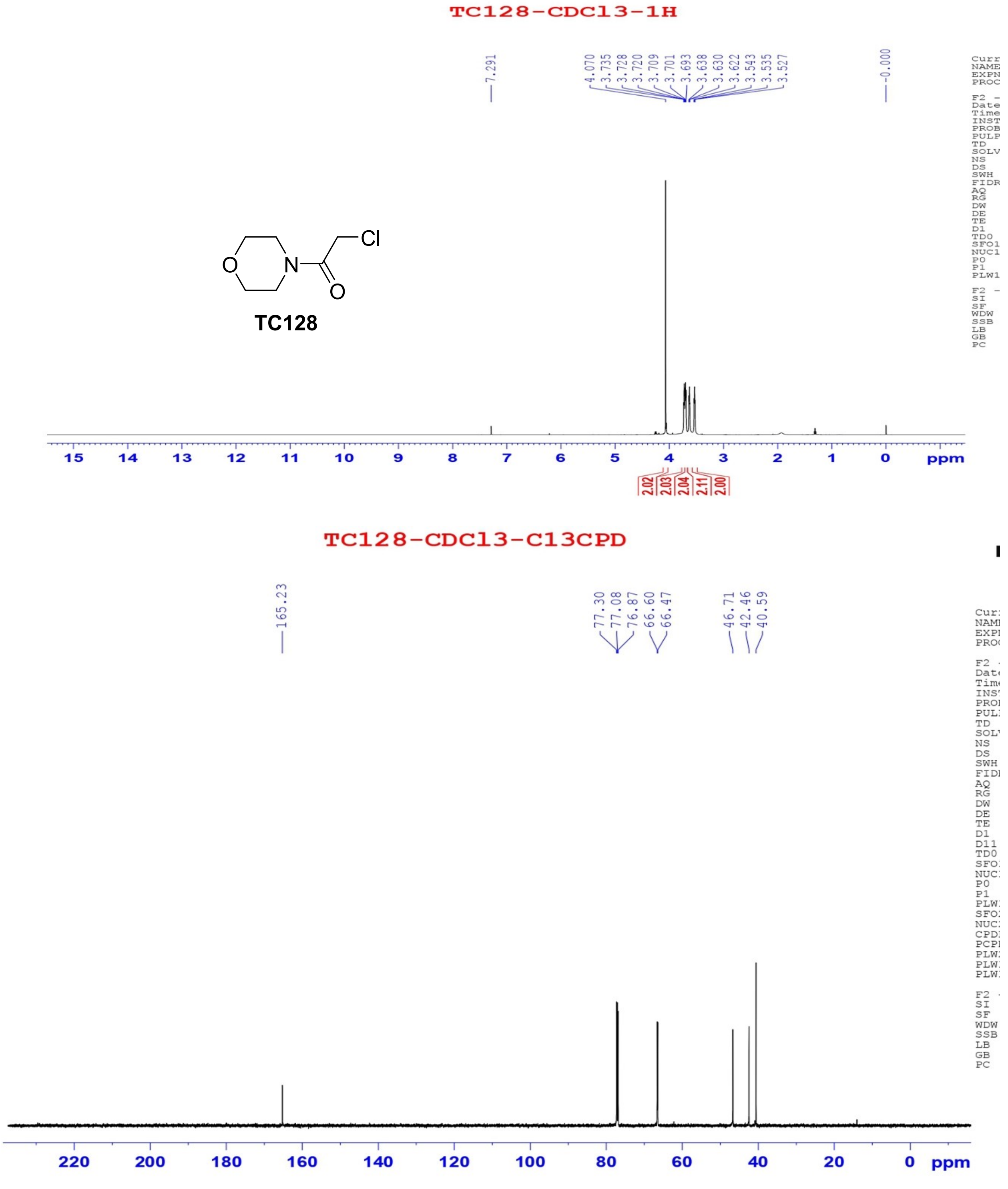


**Figure S16**. $^{1}$H-NMR and $^{13}$C-NMR spectra of compound **2k**

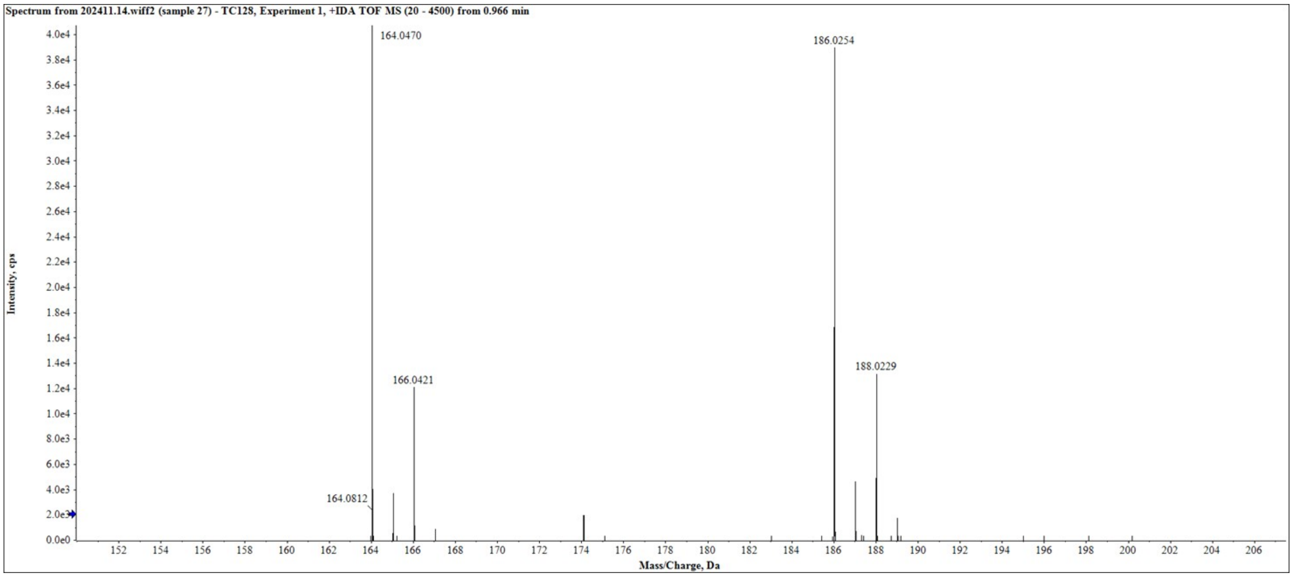


| Hit | Formula | m/z | RDB | ppm | MS Rank | MSMS ppm | MSMS Rank | Found |
|---|---|---|---|---|---|---|---|---|
| 1 | C6H10ClNO2 | 164.04728 | 2.0 | -1.7 | 1 | | | NA/NA |

**Figure S17**. HRMS spectrum of compound **2k**

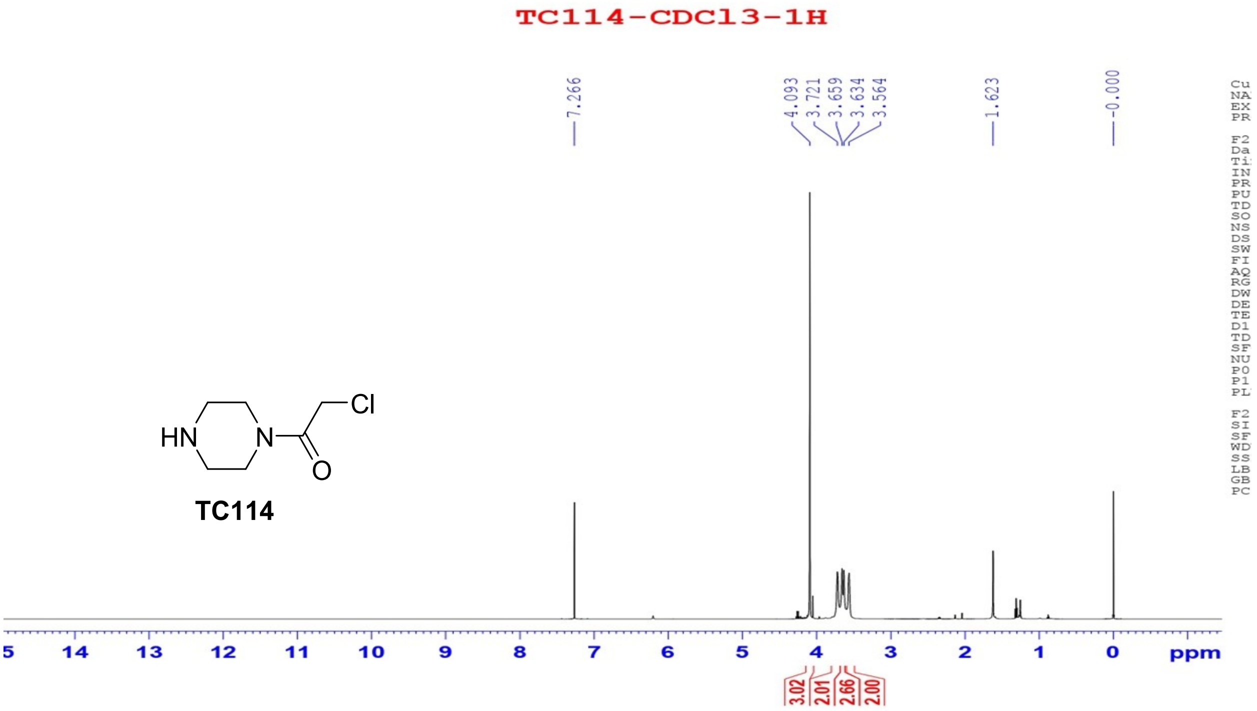


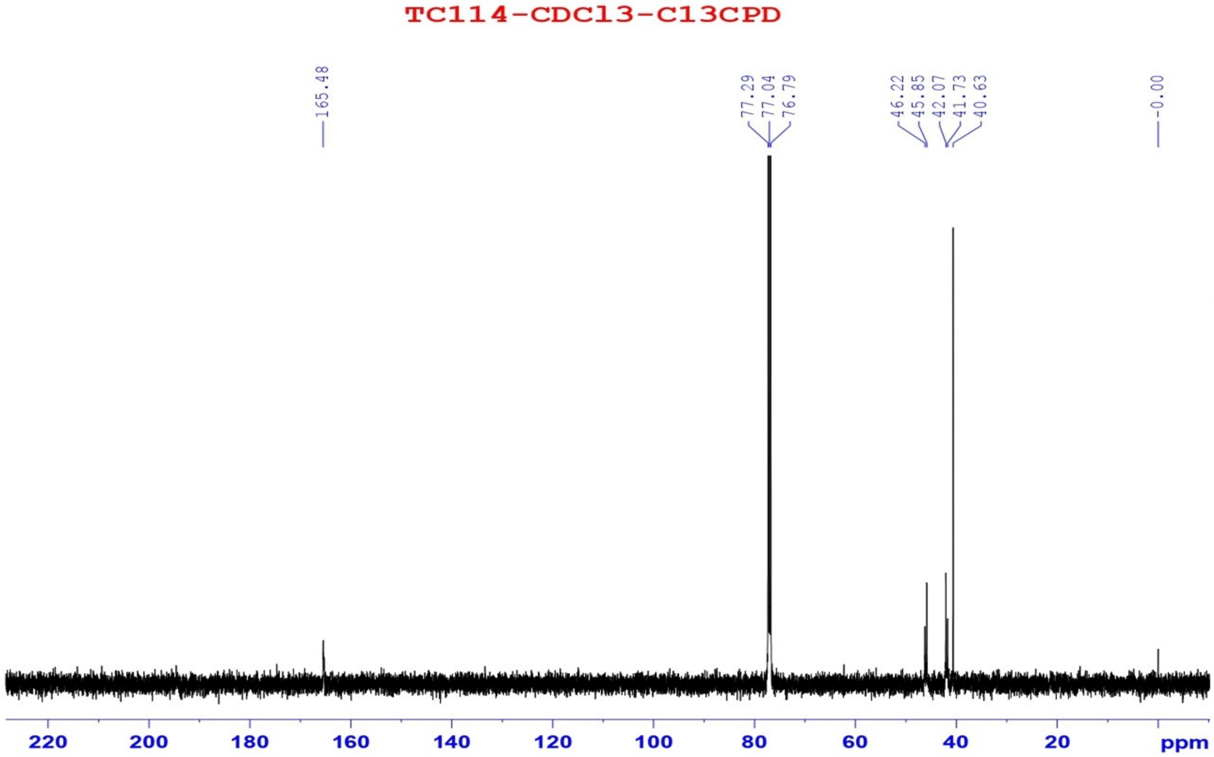


**Figure S18**. $^{1}$H-NMR and $^{13}$C-NMR spectra of compound **2l**

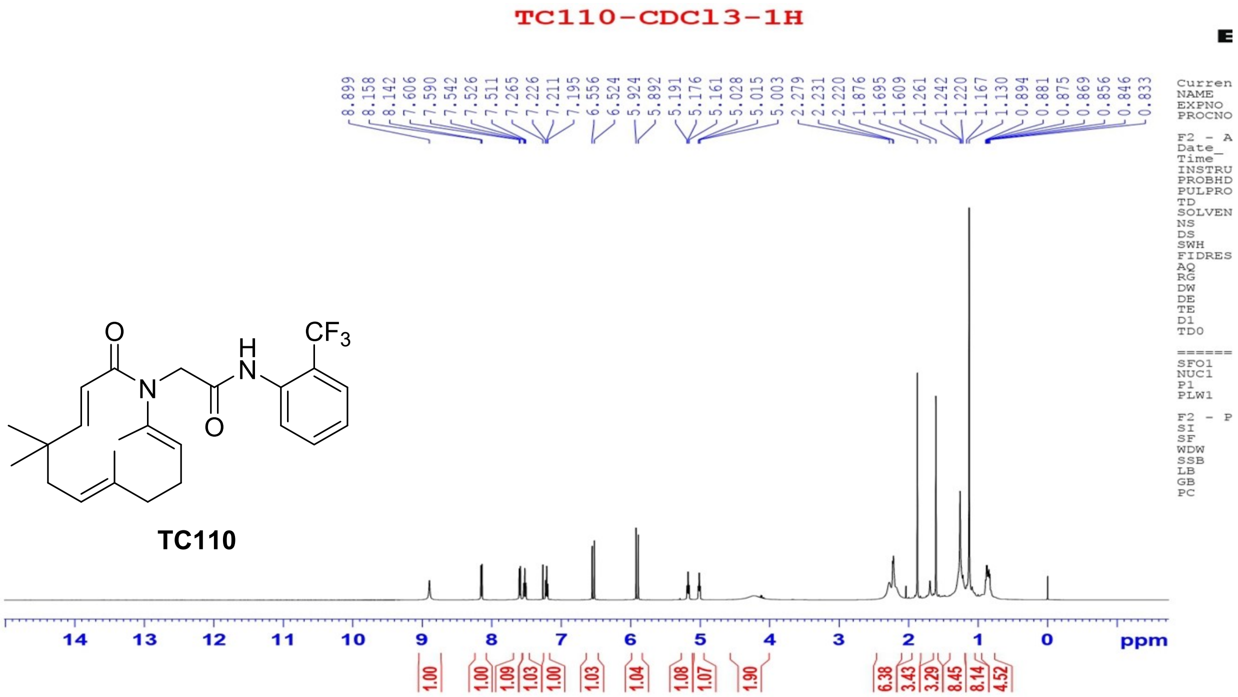
TC110-CDCl3-1H
8.899
8.158
8.142
7.606
7.590
7.542
7.526
7.511
7.265
7.226
7.211
7.195
6.556
6.524
5.924
5.892
5.191
5.176
5.161
5.028
5.015
5.003
2.279
2.231
2.220
1.876
1.695
1.609
1.261
1.242
1.220
1.167
1.130
0.894
0.881
0.875
0.869
0.856
0.846
0.833
CF3
TC110
14
13
12
11
10
9
8
7
6
5
4
3
2
1
0
ppm
1.00
1.00
1.09
1.03
1.00
1.03
1.04
1.08
1.07
1.90
6.38
3.43
3.29
8.45
8.14
4.52

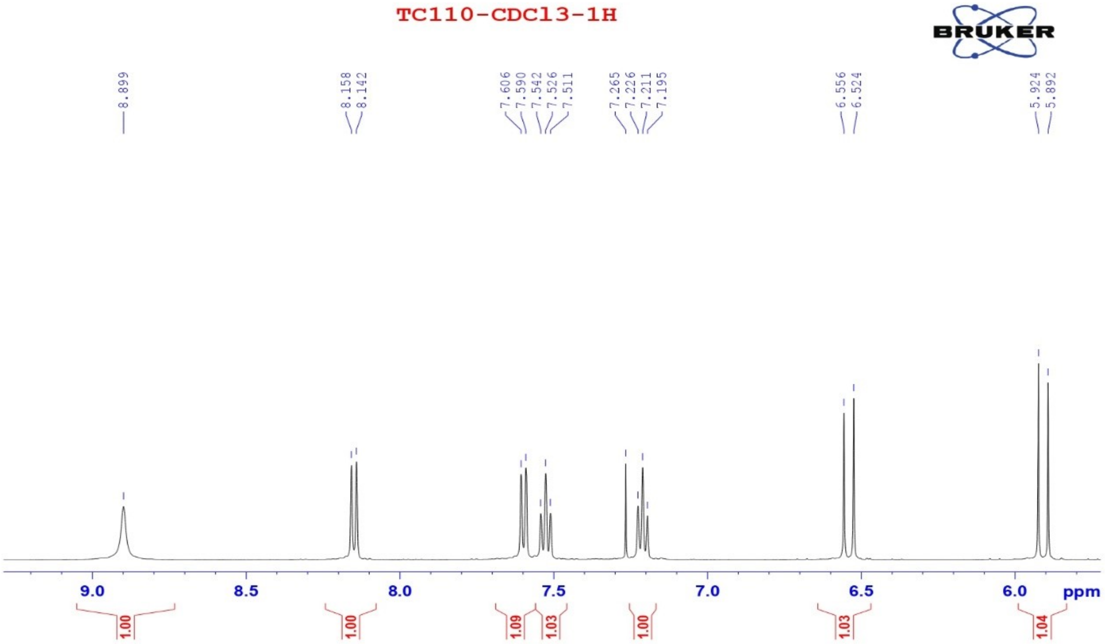


**Figure S19**. $^{1}$H-NMR spectrum of compound **4a**

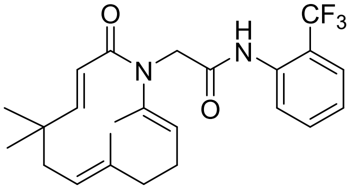

**TC110**

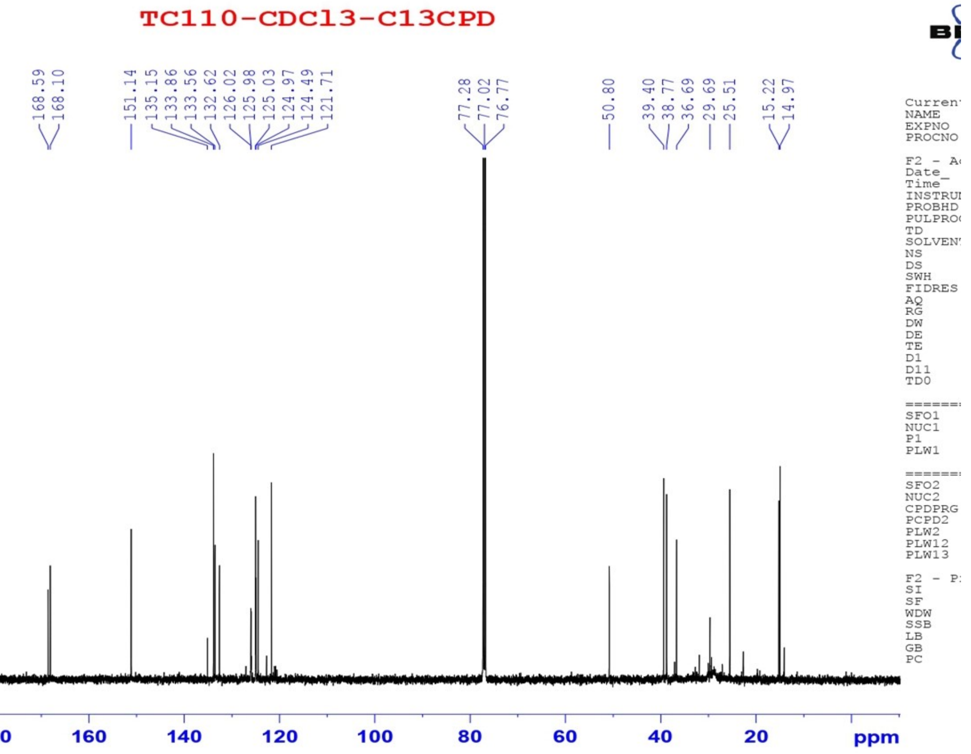


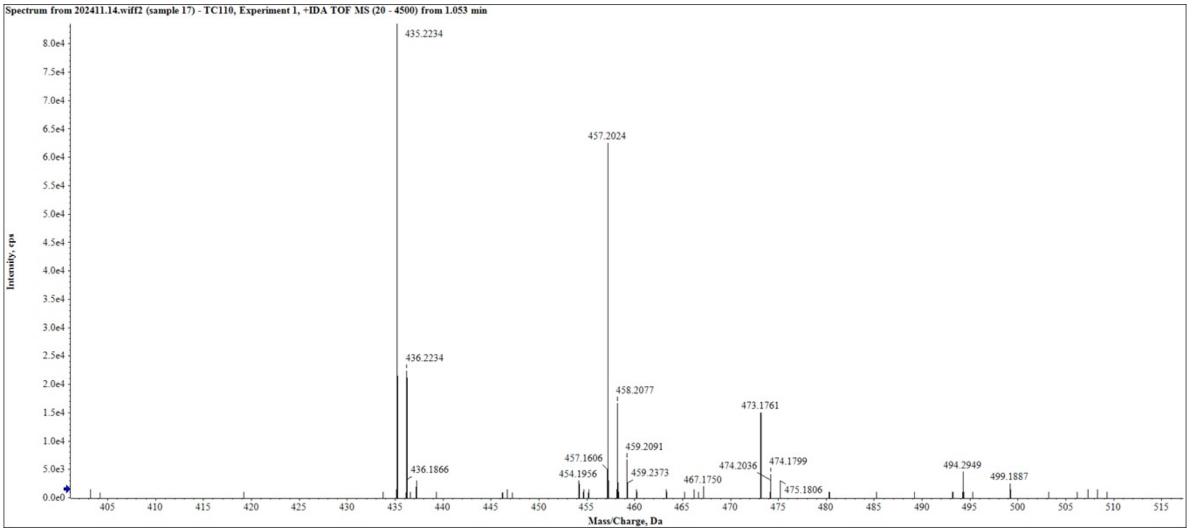


| Hit | Formula | m/z | RDB | ppm | MS Rank | MSMS ppm | MSMS Rank | Found |
|---|---|---|---|---|---|---|---|---|
| 1 | C24H29F3N2O2 | 435.22539 | 10.0 | -4.6 | 1 | | | NA/NA |

**Figure S20**. $^{13}$C-NMR and HRMS spectra of compound **4a**

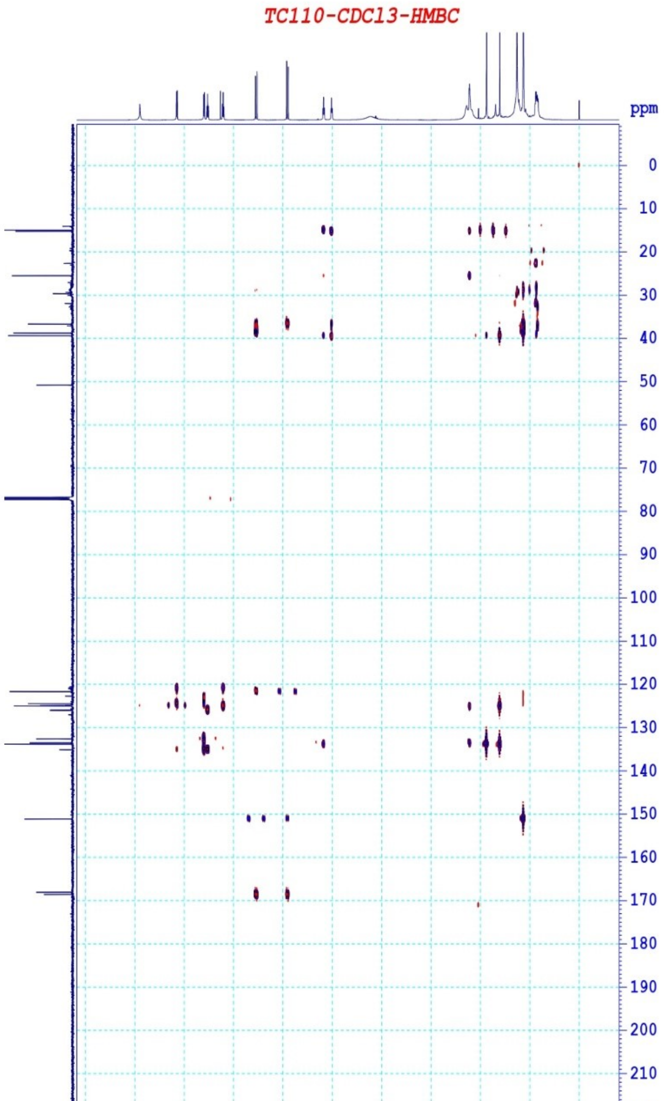
TC110-CDCl3-HMBC
ppm
0
10
20
30
40
50
60
70
80
90
100
110
120
130
140
150
160
170
180
190
200
210

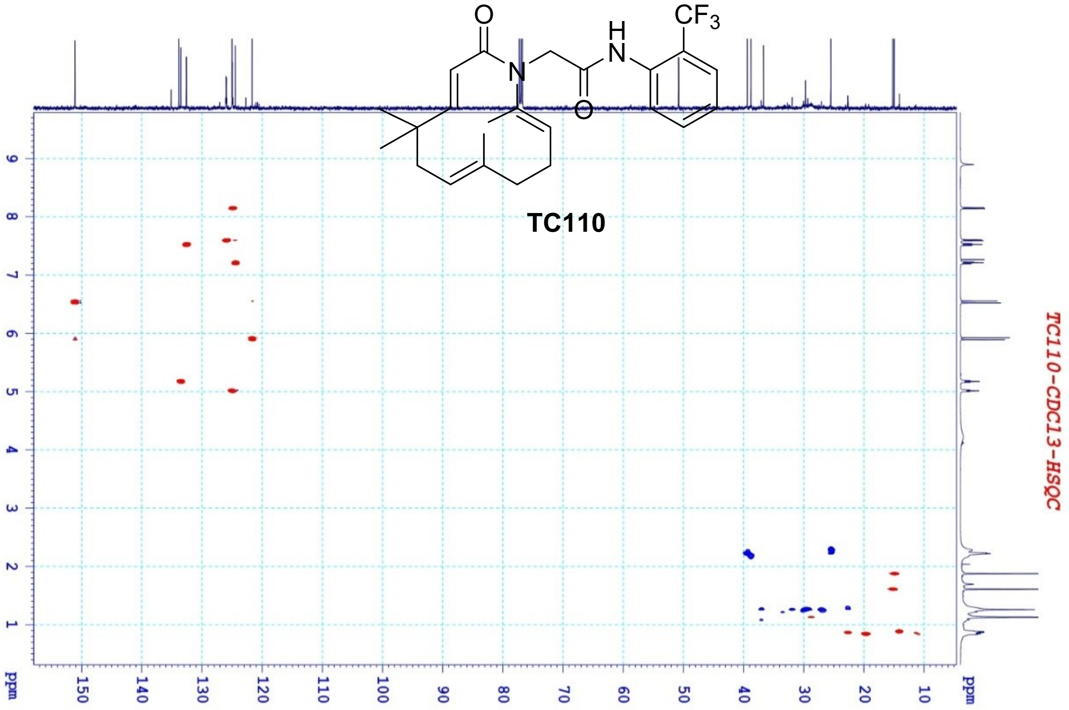


**Figure S21**. HSQC and HMBC spectra of compound **4a**

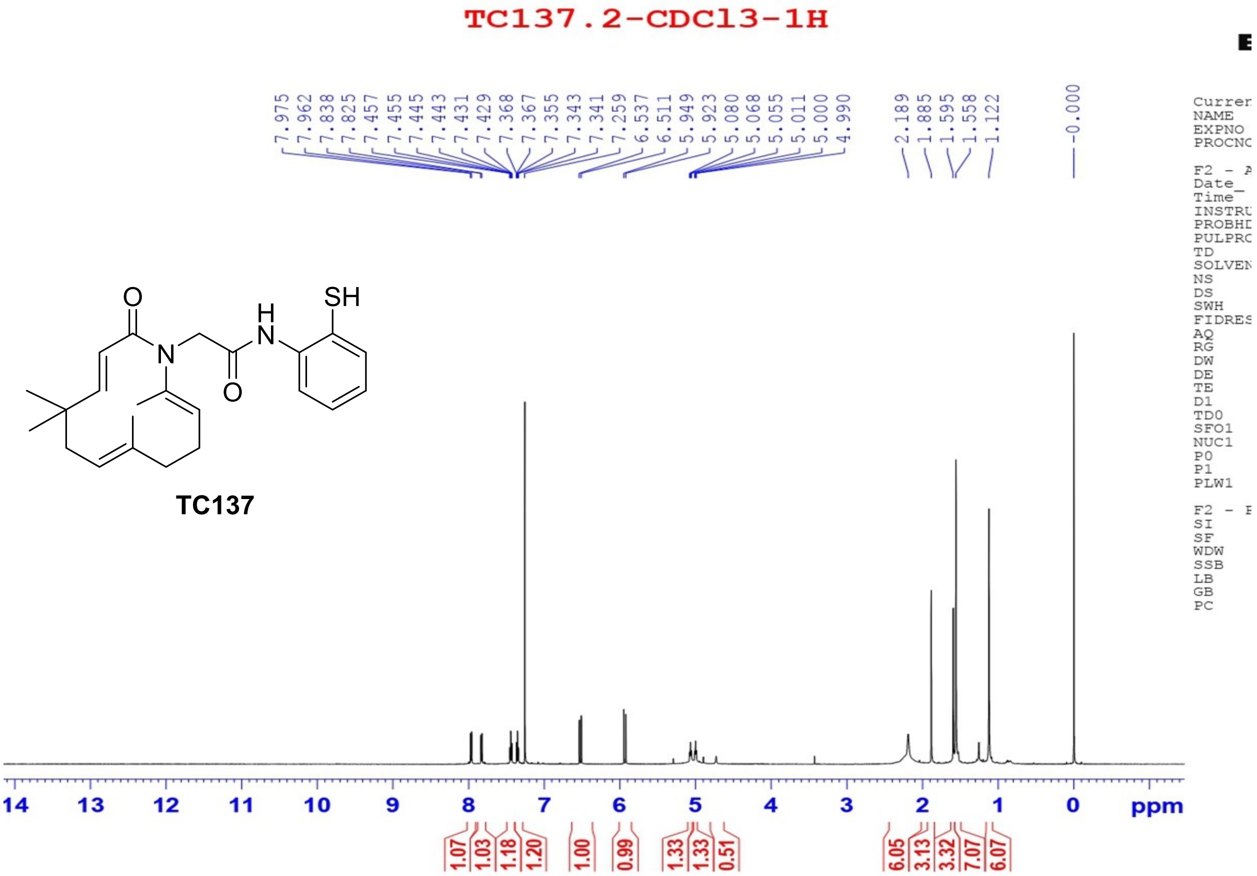


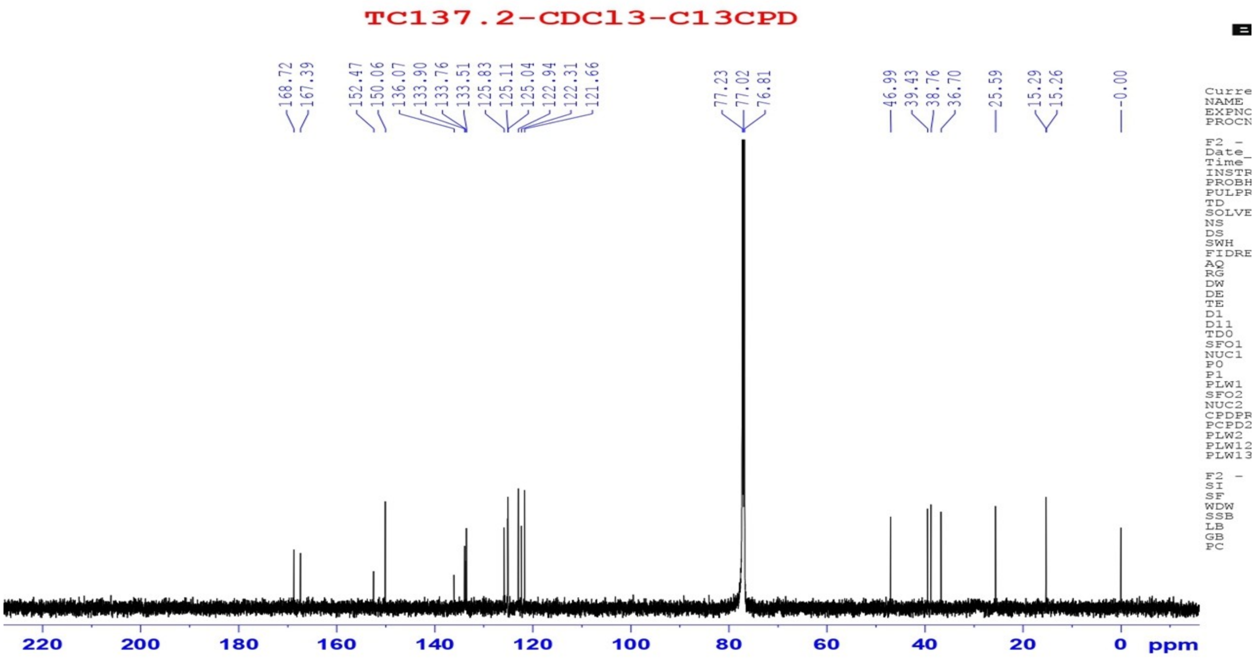


**Figure S22**. $^{1}H$ NMR and $^{13}C$ NMR spectra of compound **4b**

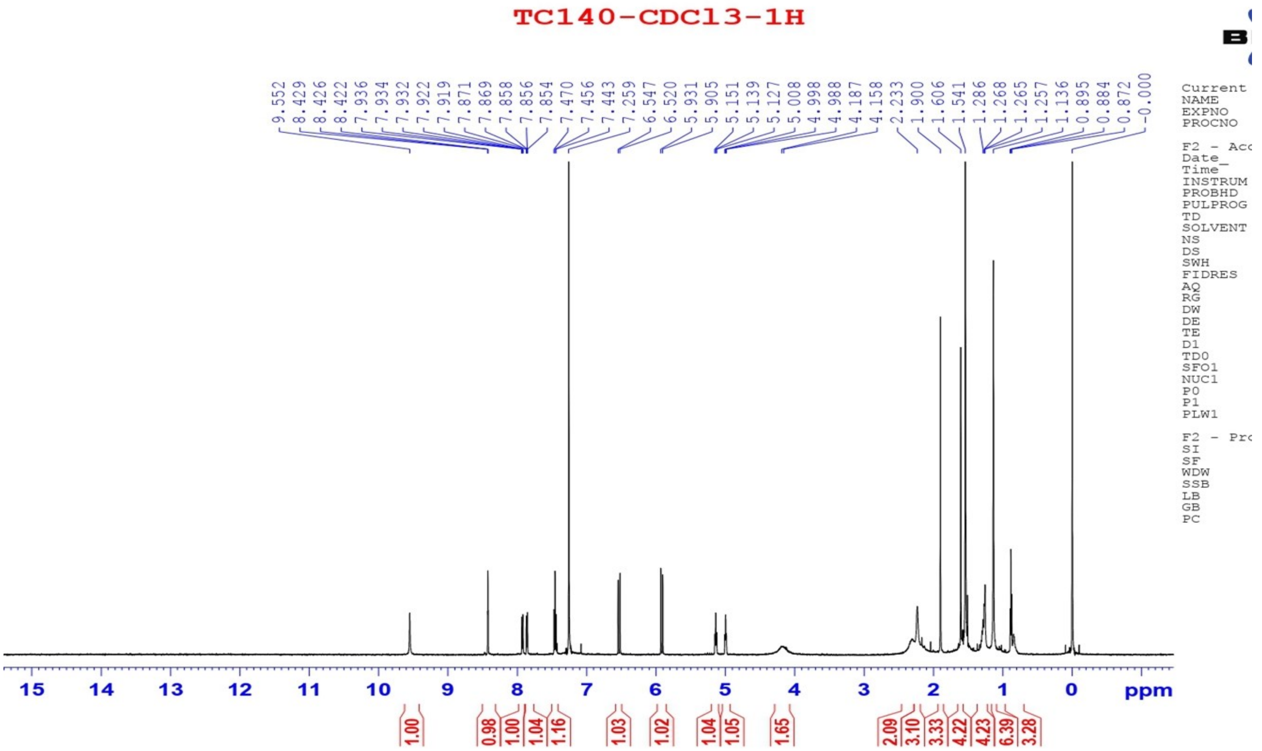
TC140-CDCl3-1H
9.552
8.429
8.426
8.422
7.936
7.934
7.932
7.922
7.919
7.871
7.869
7.858
7.856
7.854
7.470
7.456
7.443
7.259
6.547
6.520
5.931
5.905
5.151
5.139
5.127
5.008
4.998
4.988
4.187
4.158
2.233
1.900
1.606
1.541
1.286
1.268
1.265
1.257
1.136
0.895
0.884
0.872
-0.000
Current
NAME
EXPNO
PROCNO
F2 - Acc
Date_
Time_
INSTRUM
PROBHD
PULPROG
TD
SOLVENT
NS
DS
SWH
FIDRES
AQ
RG
DW
DE
TE
D1
TD0
SFO1
NUC1
P0
P1
PLW1
F2 - Pro
SI
SF
WDW
SSB
LB
GB
PC
15
14
13
12
11
10
9
8
7
6
5
4
3
2
1
0
ppm
1.00
0.98
1.00
1.04
1.16
1.03
1.02
1.04
1.05
1.65
2.09
3.10
3.33
4.22
4.23
6.39
3.28

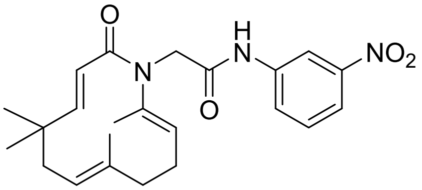
O
N
H
N
NO2
O

**TC140**

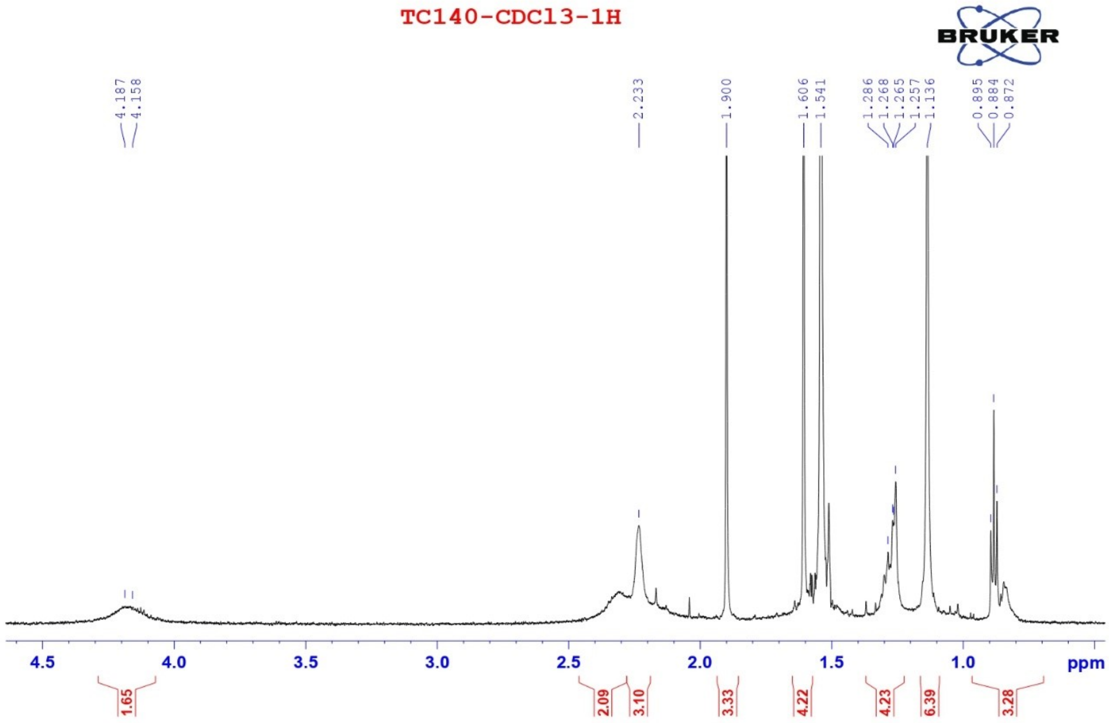


**Figure S23**. $^{1}$H NMR spectrum of compound **4c**

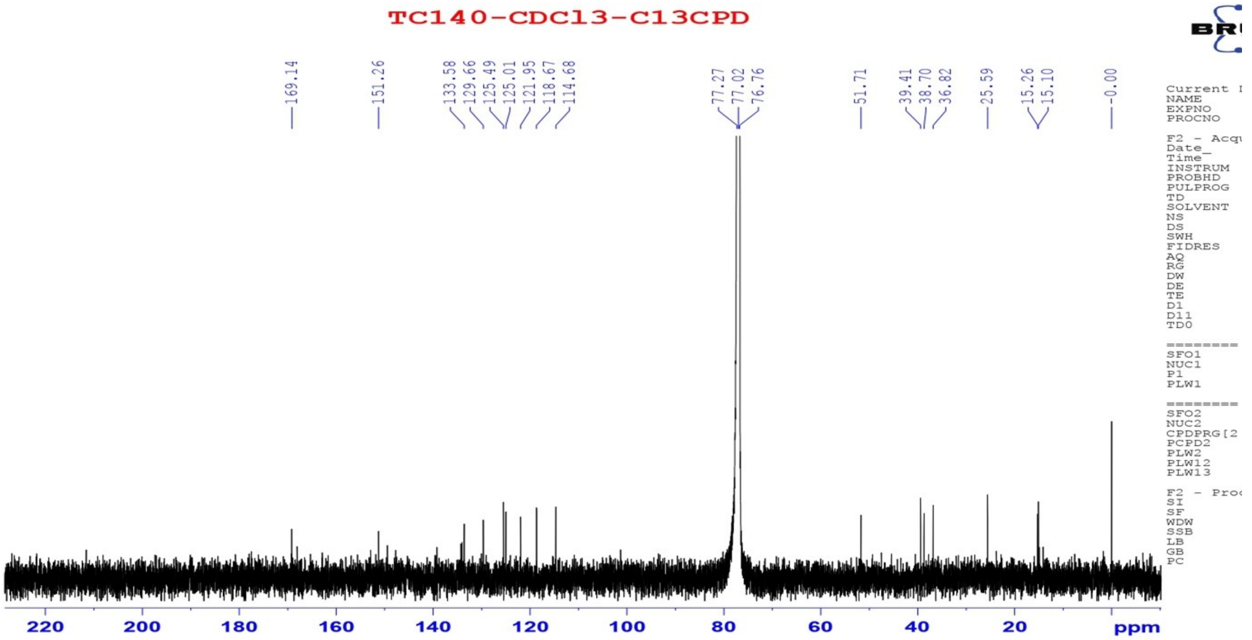


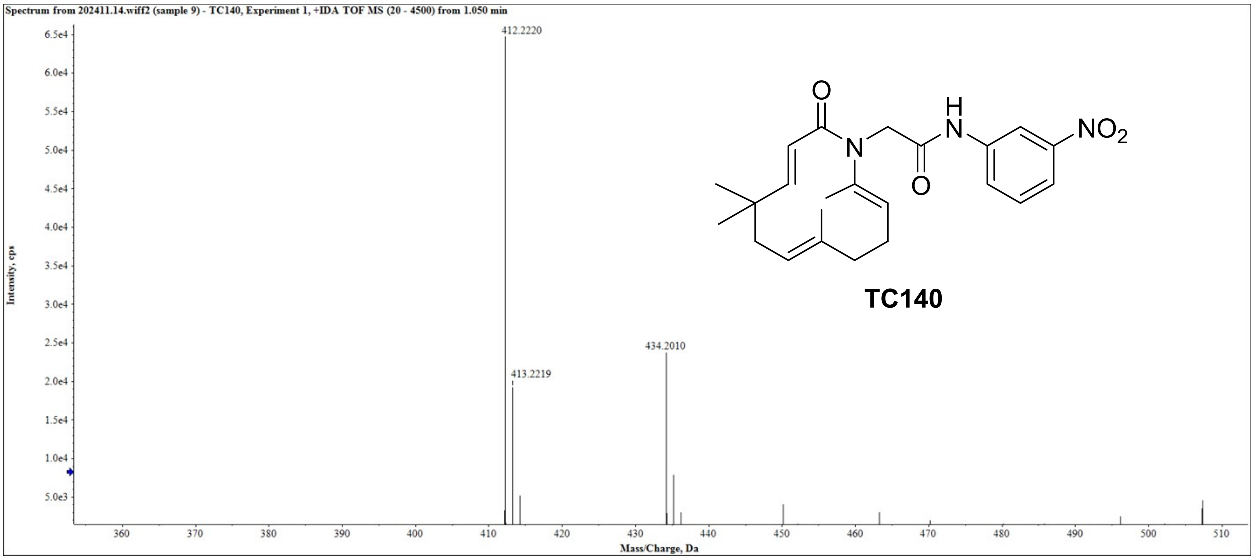


| Hit | Formula | m/z | RDB | ppm | MS Rank | MSMS ppm | MSMS Rank | Found |
|---|---|---|---|---|---|---|---|---|
| 1 | C23H29N3O4 | 412.22308 | 11.0 | -2.7 | 1 | | | NA/NA |

**Figure S24**. $^{13}C$ NMR and HRMS spectra of compound **4c**

TC142.2-CDCl3-1H

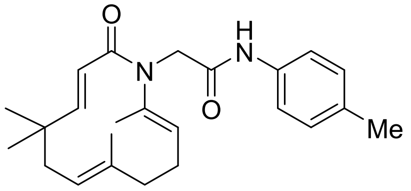

TC142.2

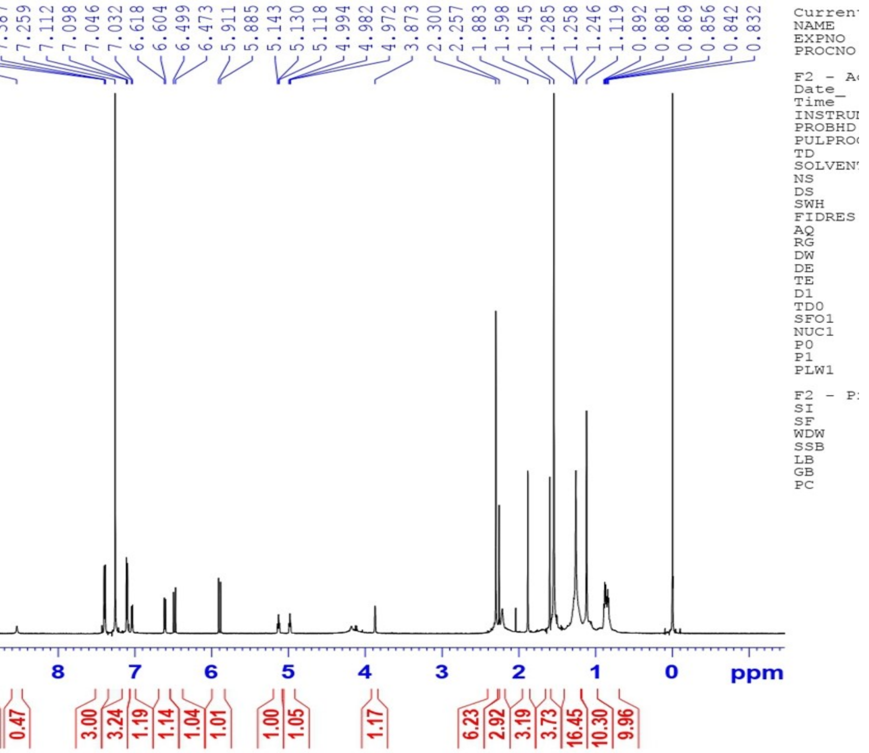

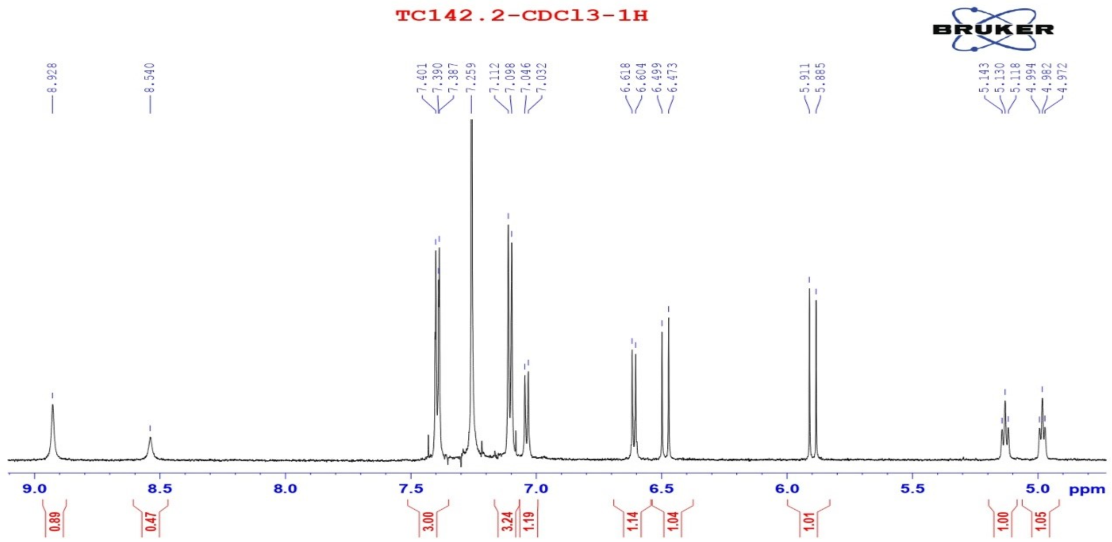


**Figure S25**. $^{1}$H NMR spectrum of compound **4d**

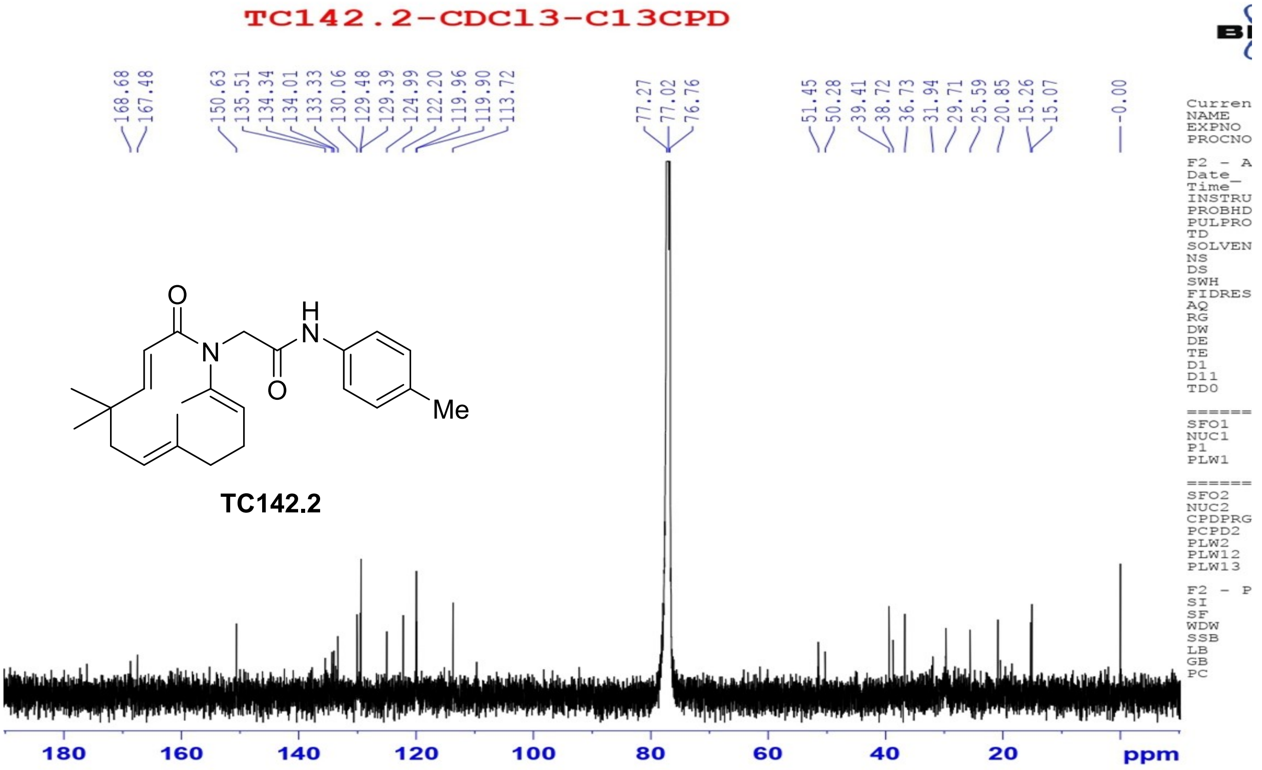


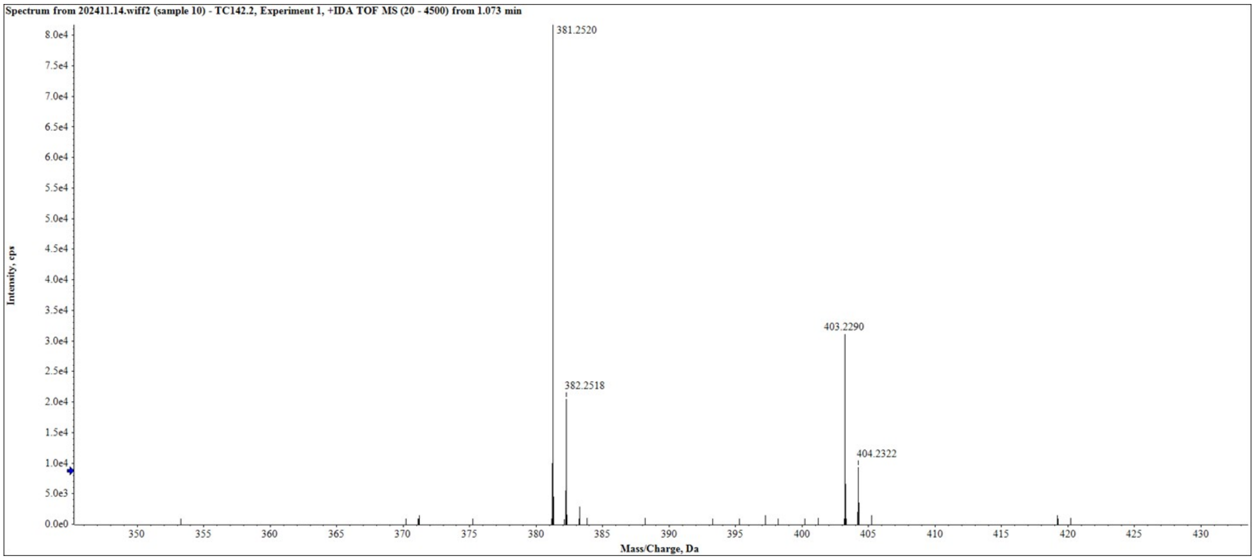


| Hit | Formula | m/z | RDB | ppm | MS Rank | MSMS ppm | MSMS Rank | Found |
|---|---|---|---|---|---|---|---|---|
| 1 | C24H32N2O2 | 381.25365 | 10.0 | -4.3 | 1 | | | NA/NA |

**Figure S26**. $^{13}$C NMR and HRMS spectra of compound **4d**

TC126-CDCl3-1H

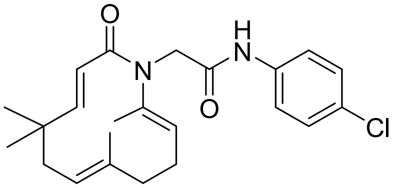


TC126

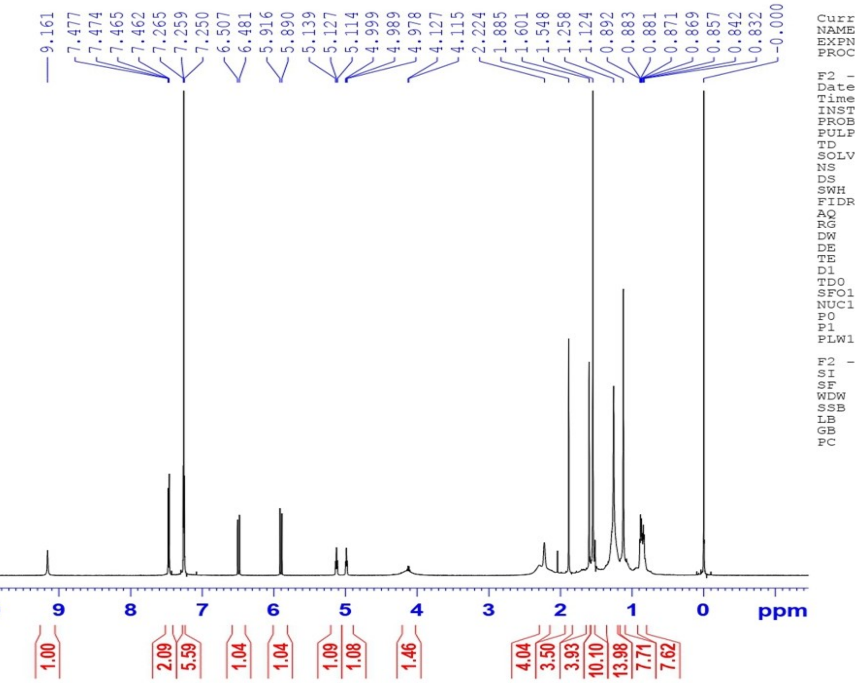

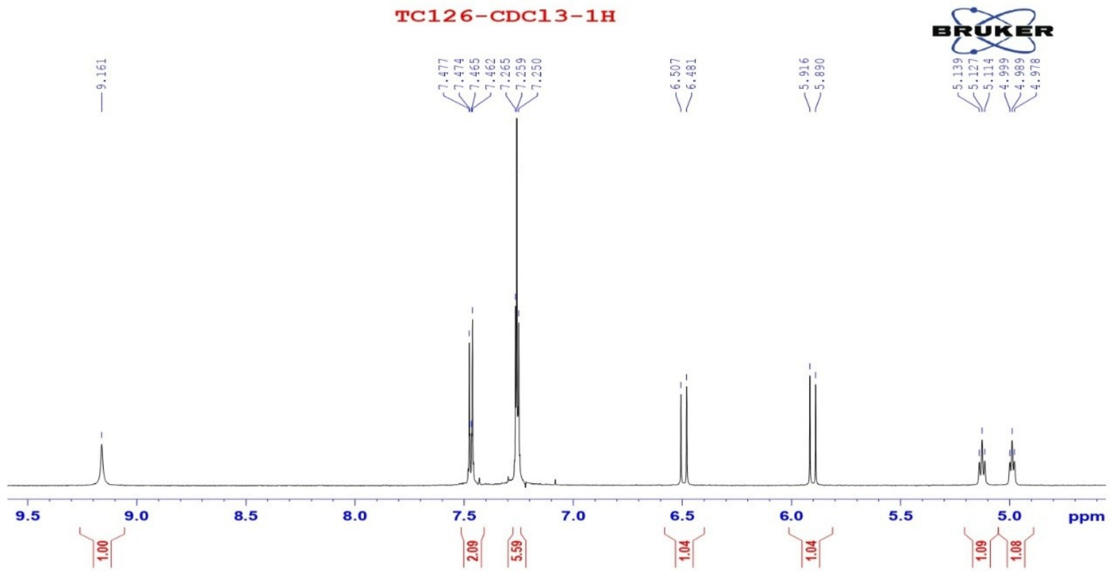


**Figure S27**. $^{1}$H NMR spectrum of compound **4e**

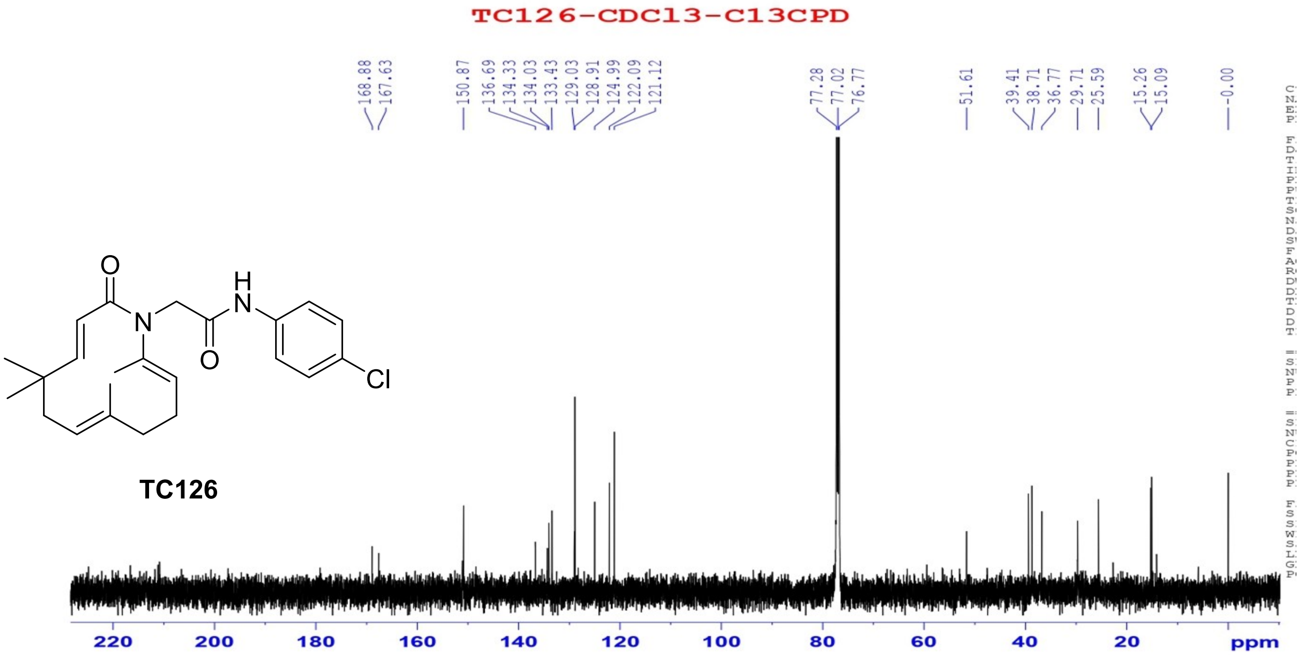


TC126

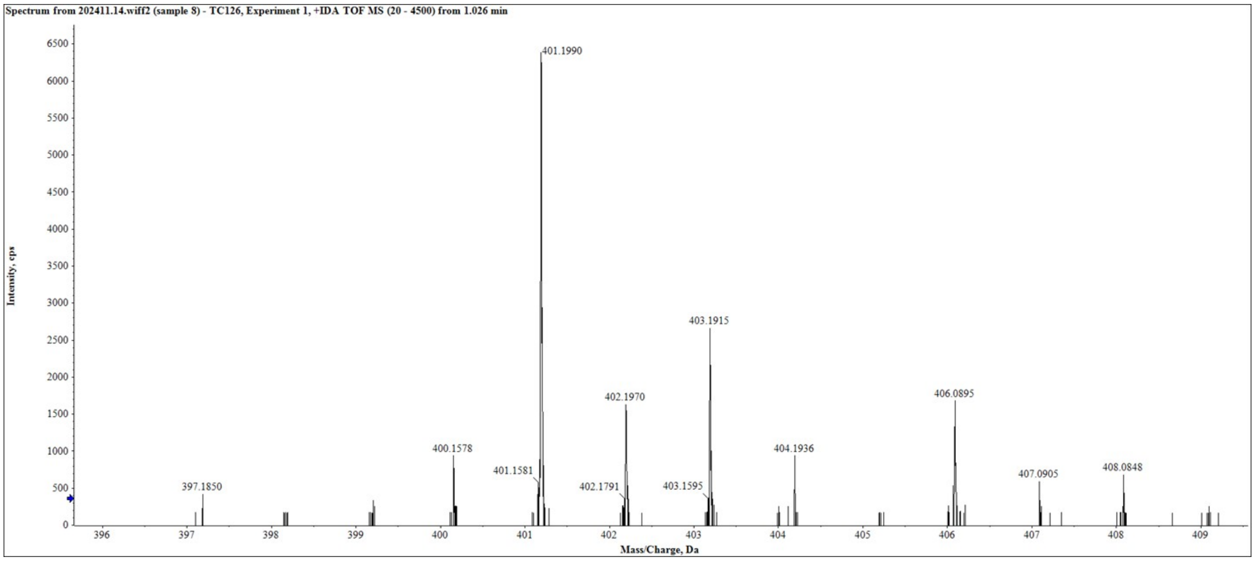


| Hit | Formula | m/z | RDB | ppm | MS Rank | MSMS ppm | MSMS Rank | Found |
|---|---|---|---|---|---|---|---|---|
| 1 | C23H29ClN2O2 | 401.19903 | 10.0 | -0.1 | 1 | | | NA/NA |

**Figure S28**. $^{13}$C NMR and HRMS spectra of compound **4e**

TC146-CDCl3-1H

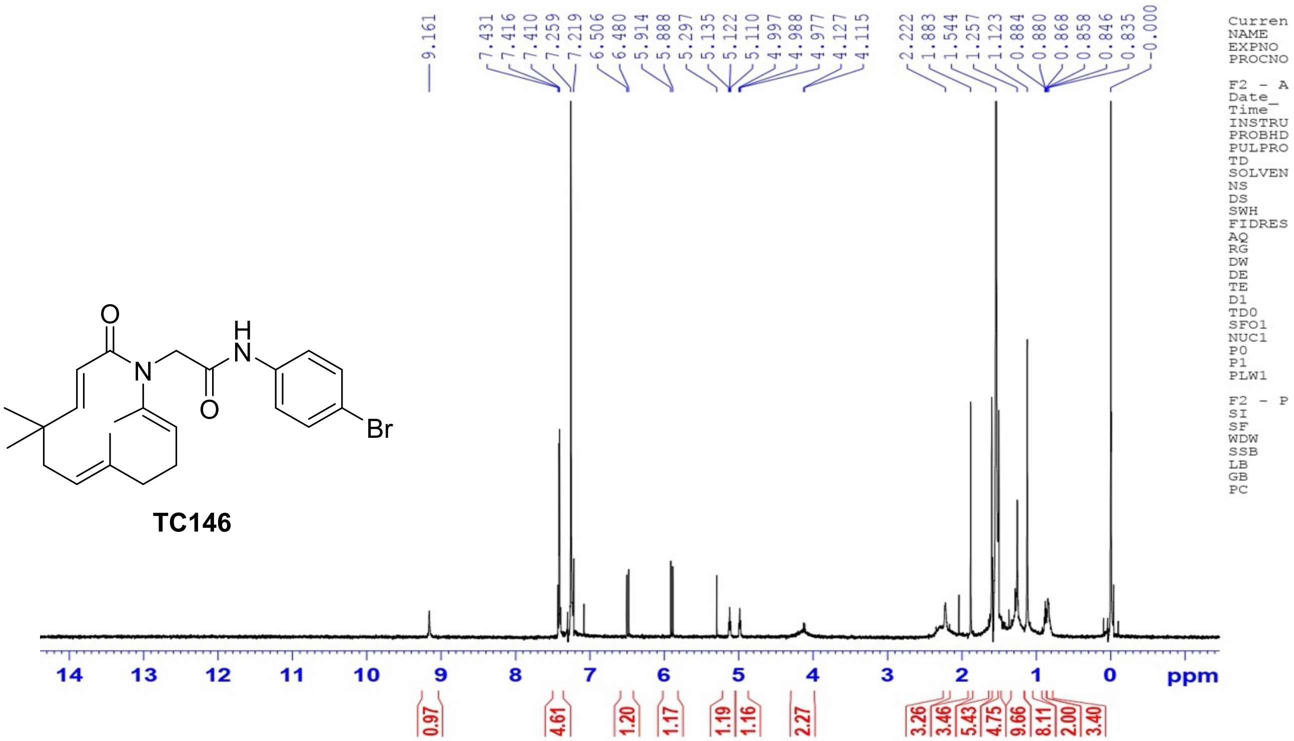

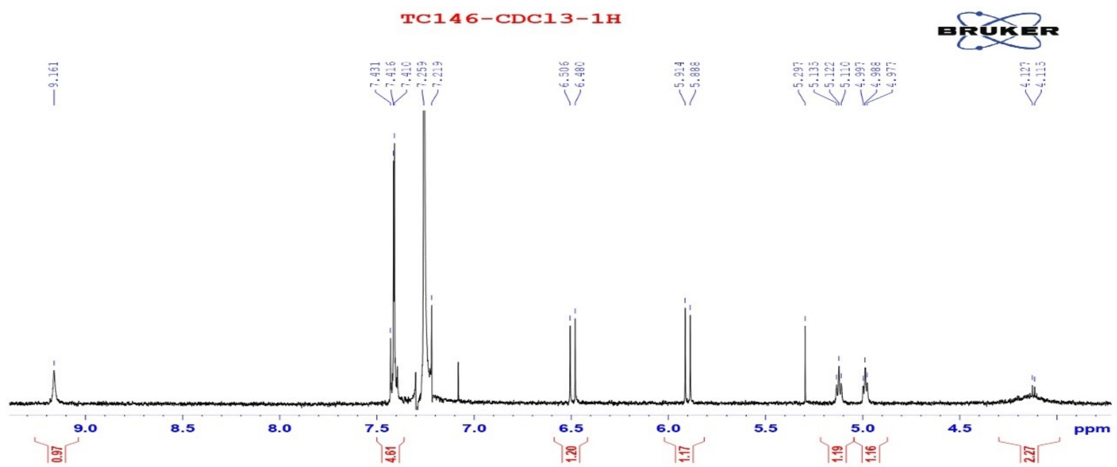


**Figure S29**. $^{1}$H NMR spectrum of compound **4f**

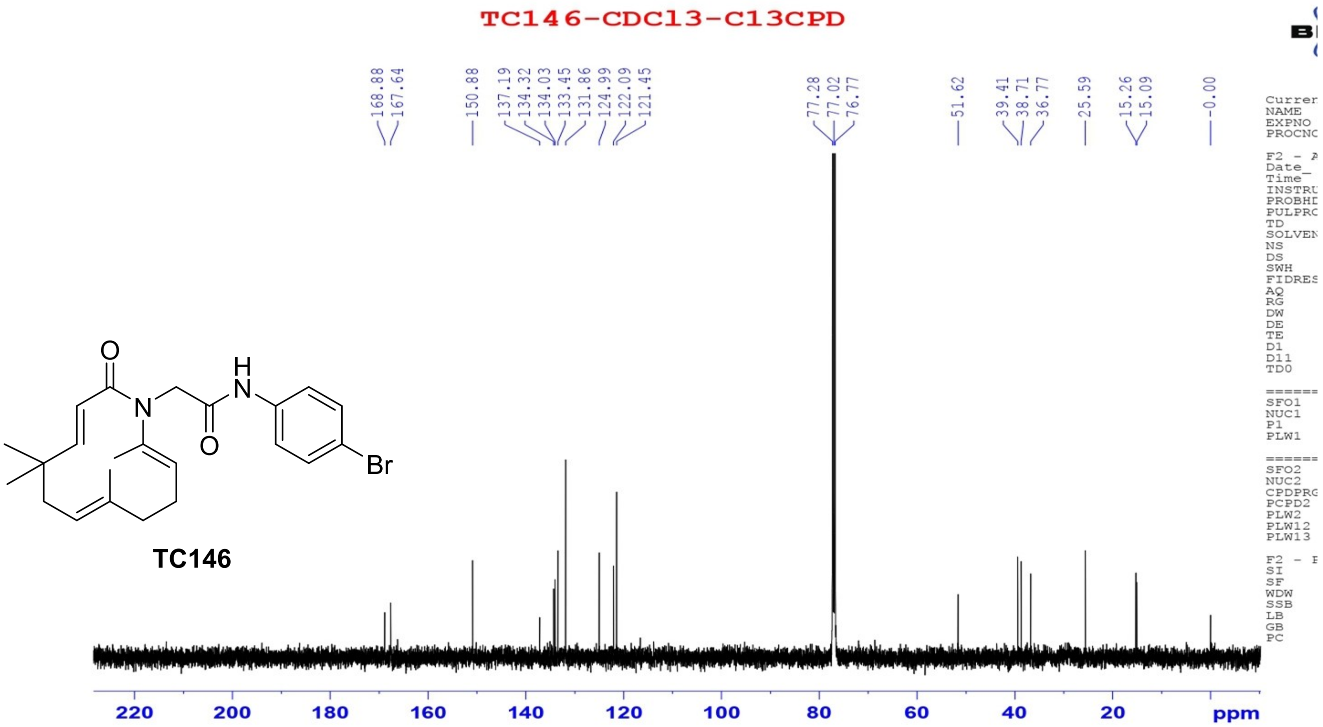


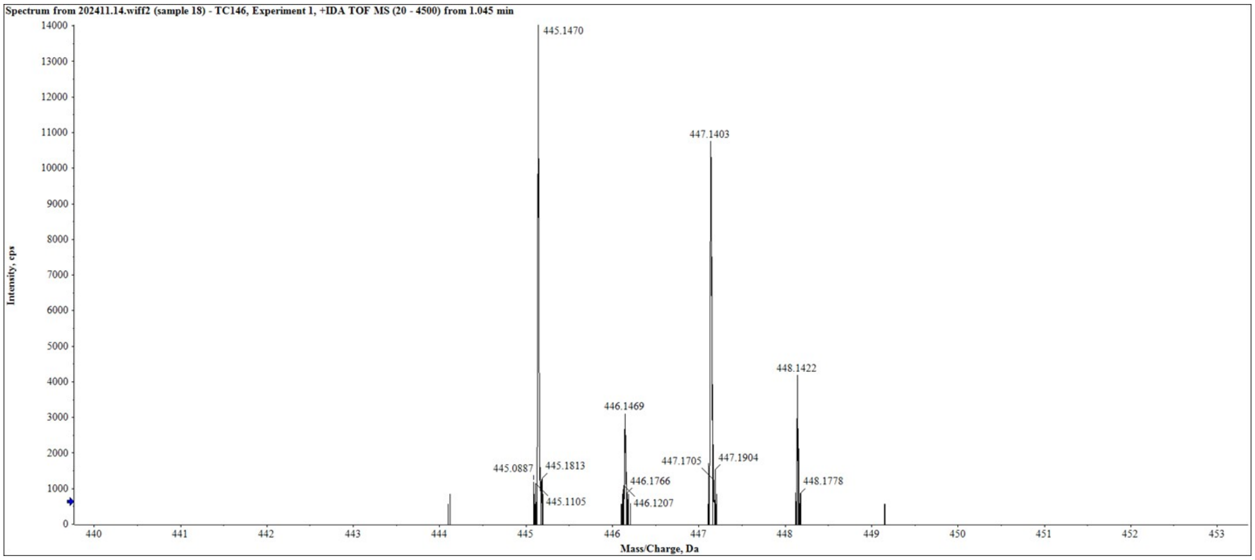


| Hit | Formula | m/z | RDB | ppm | MS Rank | MSMS ppm | MSMS Rank | Found |
|---|---|---|---|---|---|---|---|---|
| 1 | C23H29BrN2O2 | 445.14852 | 10.0 | -3.4 | 1 | | | NA/NA |

**Figure S30**. $^{13}C$ NMR and HRMS spectra of compound **4f**

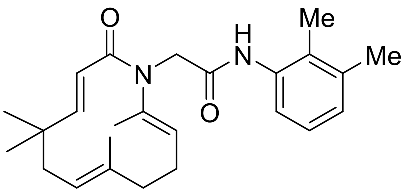
Me
Me
O
H
N
N
O

**TC120**

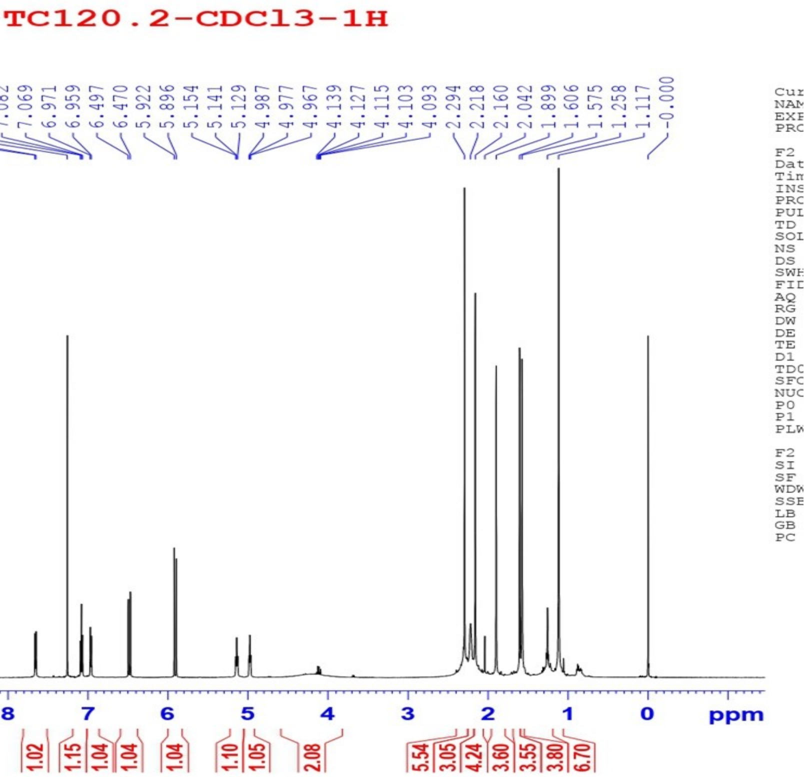
TC120.2-CDCl3-1H
8.774
7.663
7.649
7.259
7.095
7.082
7.069
6.971
6.959
6.497
6.470
5.922
5.896
5.154
5.141
5.129
4.987
4.977
4.967
4.139
4.127
4.115
4.103
4.093
2.294
2.218
2.160
2.042
1.899
1.606
1.575
1.258
1.117
-0.000
13 12 11 10 9 8 7 6 5 4 3 2 1 0 ppm
1.00
1.02
1.15
1.04
1.04
1.04
1.10
1.05
2.08
5.54
3.05
4.24
3.60
3.55
3.80
6.70

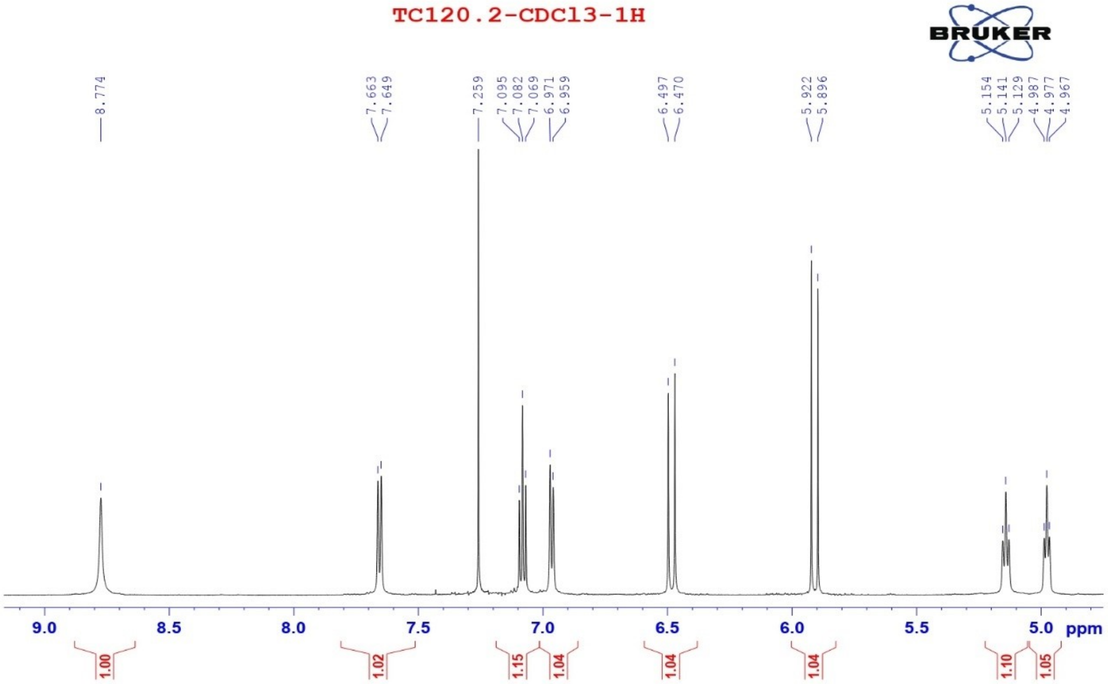


**Figure S31**. $^{1}$H NMR spectrum of compound **4g**

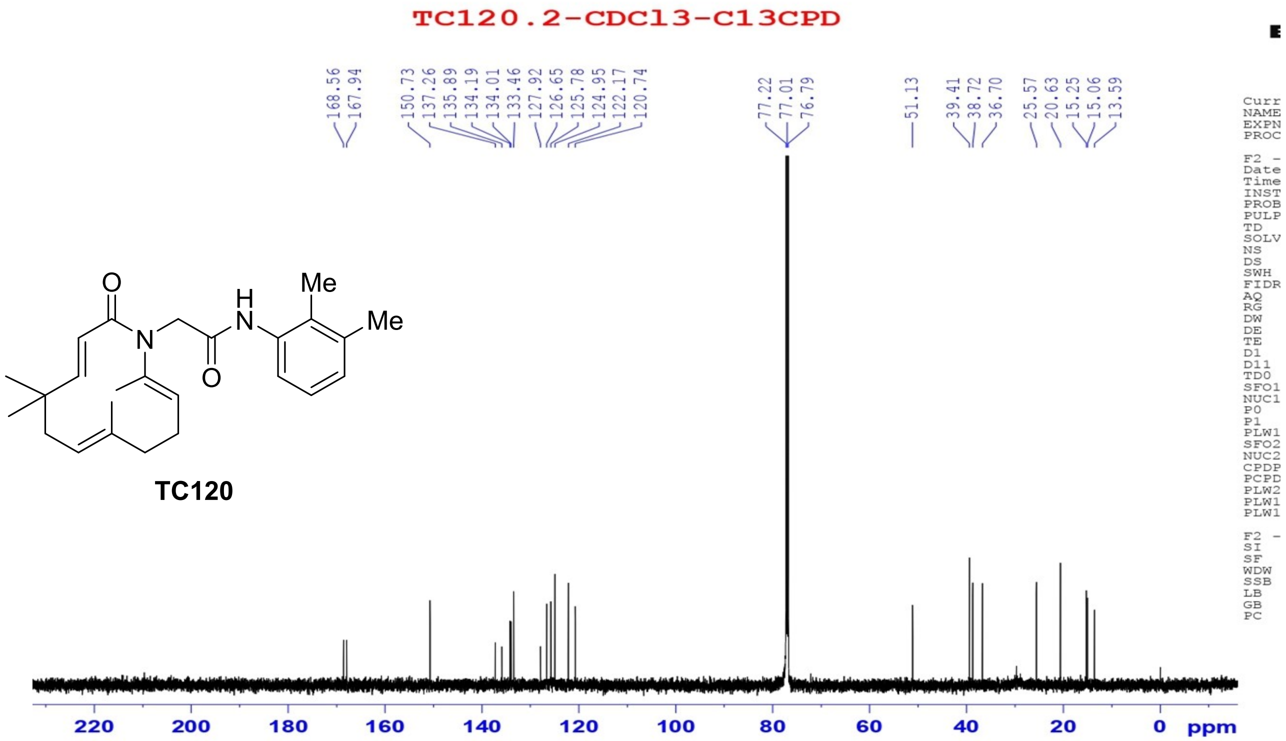


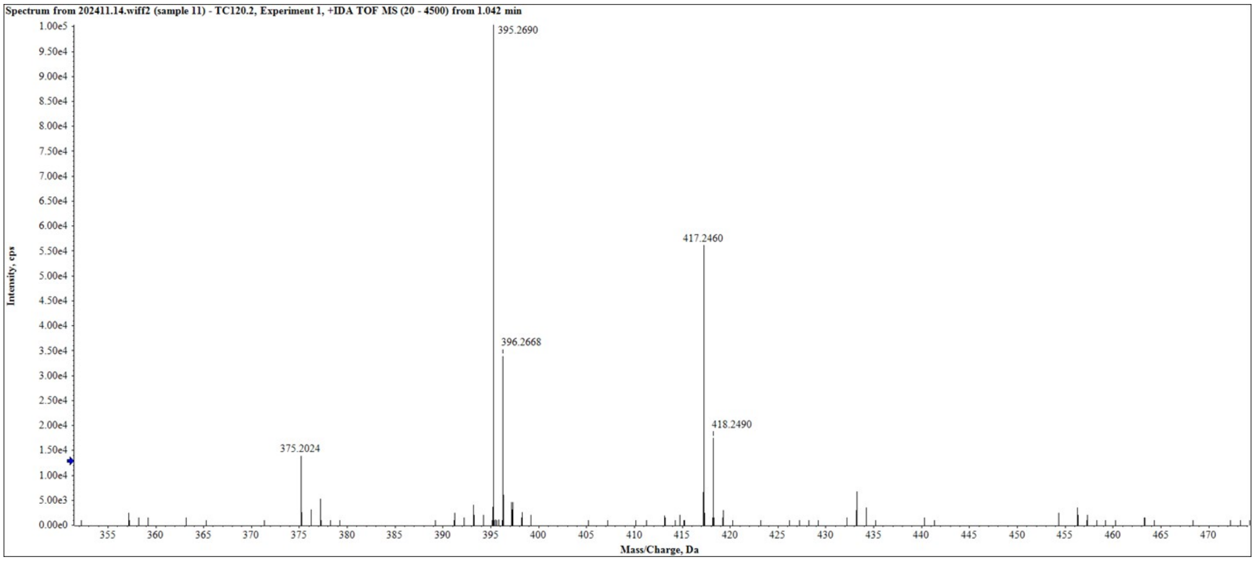


| Hit | Formula | m/z | RDB | ppm | MS Rank | MSMS ppm | MSMS Rank | Found |
|---|---|---|---|---|---|---|---|---|
| 1 | C25H34N2O2 | 395.26930 | 10.0 | -0.8 | 1 | | | NA/NA |

**Figure S32**. $^{13}$C NMR and HRMS spectra of compound **4g**

TC119.2-CDCl3-1H

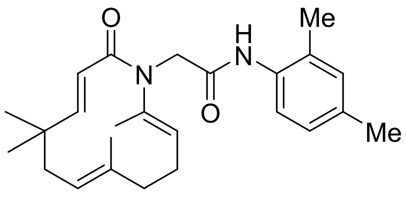

**TC119**

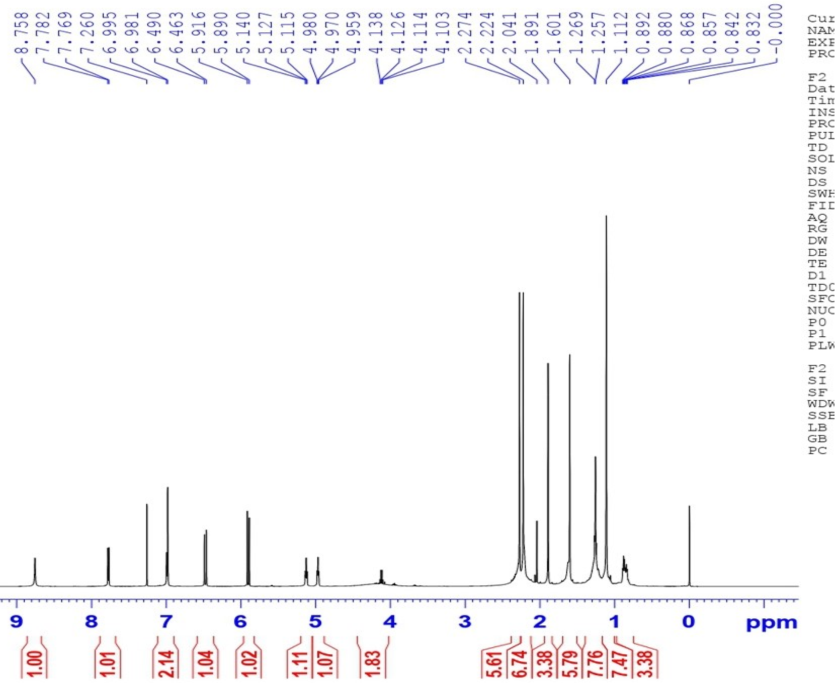

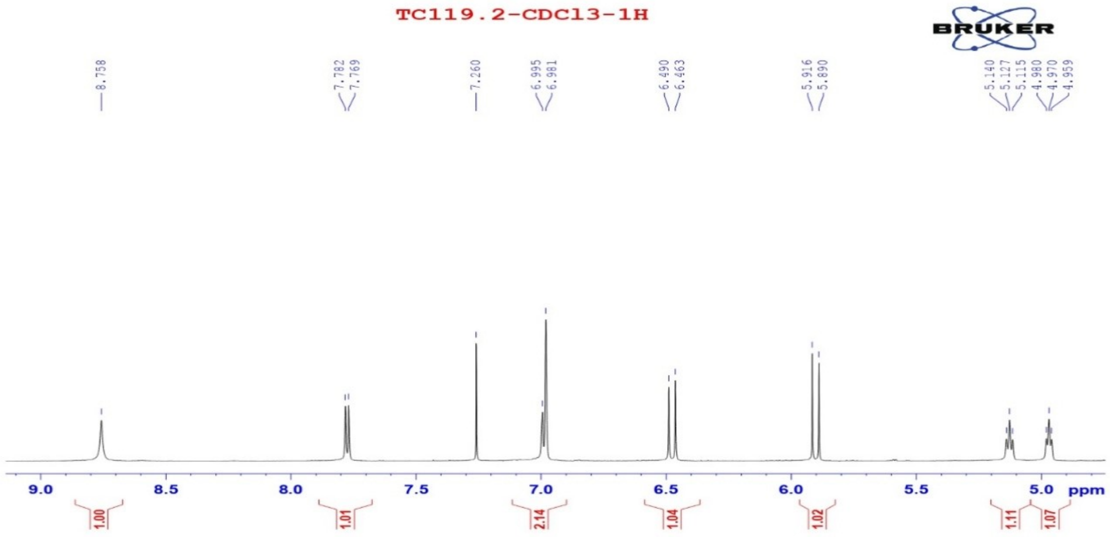


**Figure S33**. $^1$H NMR spectrum of compound **4h**

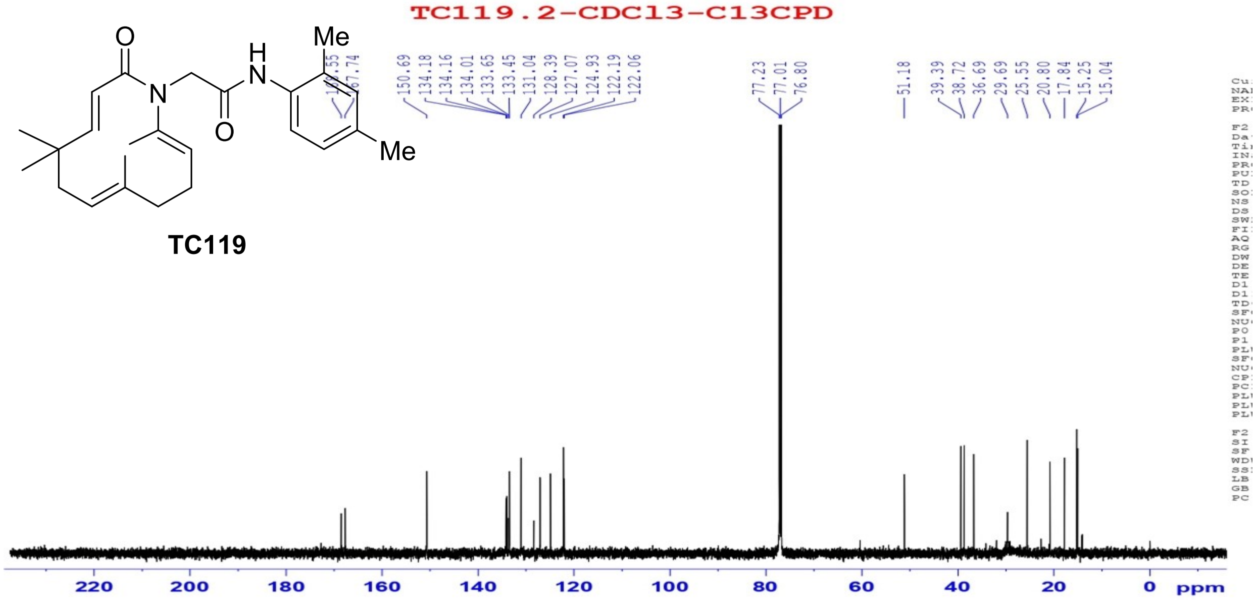
TC119.2-CDCl3-C13CPD
Me
NH
O
N
Me
TC119
150.69
134.18
134.16
134.01
133.65
133.45
131.04
128.39
127.07
124.93
122.19
122.06
77.23
77.01
76.80
51.18
39.39
38.72
36.69
29.69
25.55
20.80
17.84
15.25
15.04
220
200
180
160
140
120
100
80
60
40
20
0
ppm

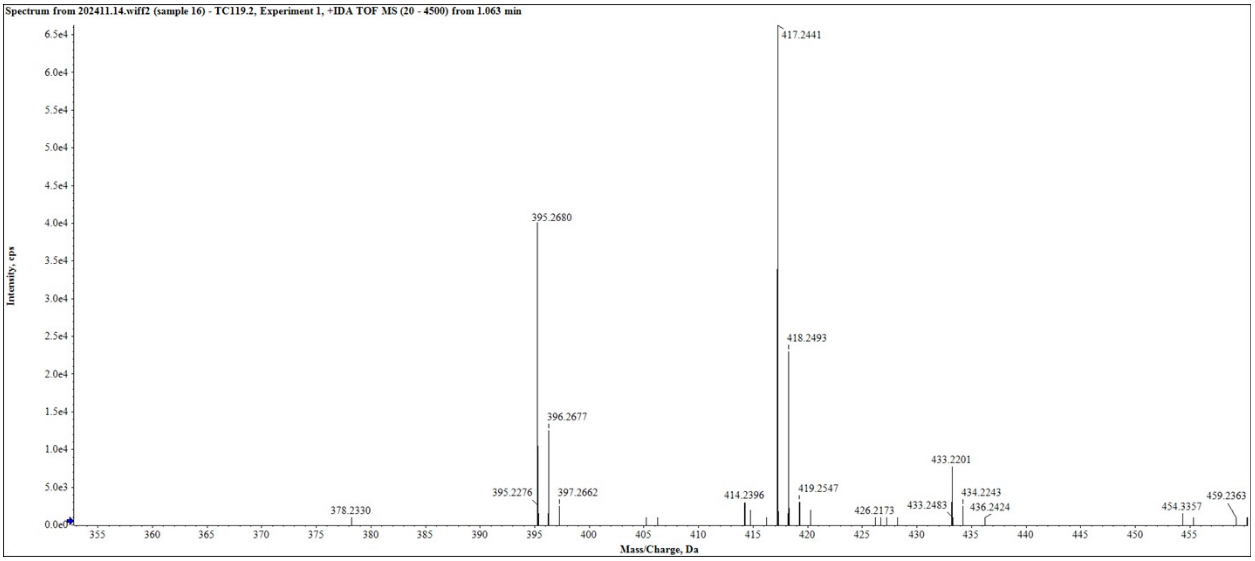


| Hit | Formula | m/z | RDB | ppm | MS Rank | MSMS ppm | MSMS Rank | Found |
|---|---|---|---|---|---|---|---|---|
| 1 | C25H34N2O2 | 395.26930 | 10.0 | -3.3 | 1 | | | NA/NA |

**Figure S34**. $^{13}$C NMR and HRMS spectra of compound **4h**

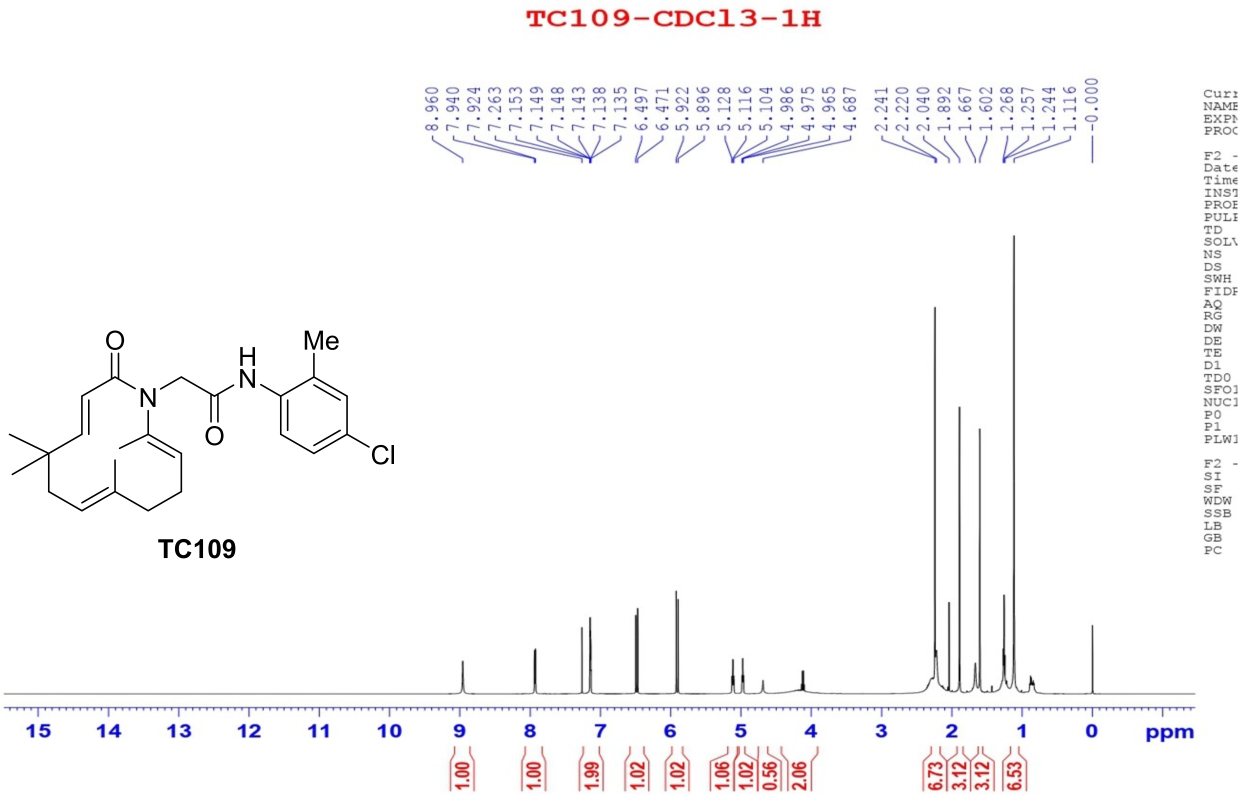

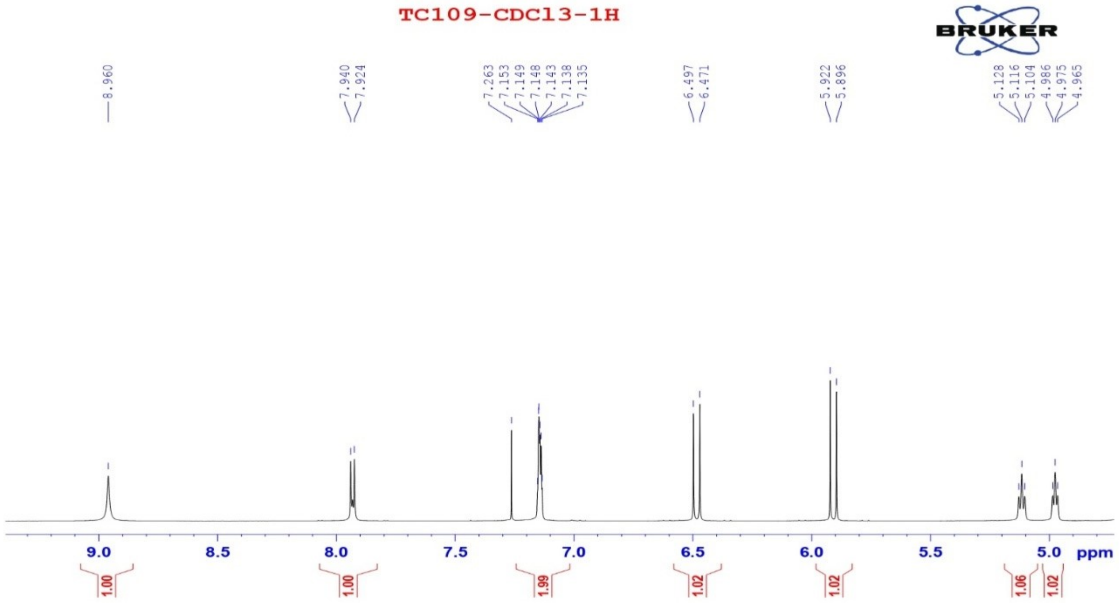


**Figure S35**. $^{1}H$ NMR spectrum of compound **4i**

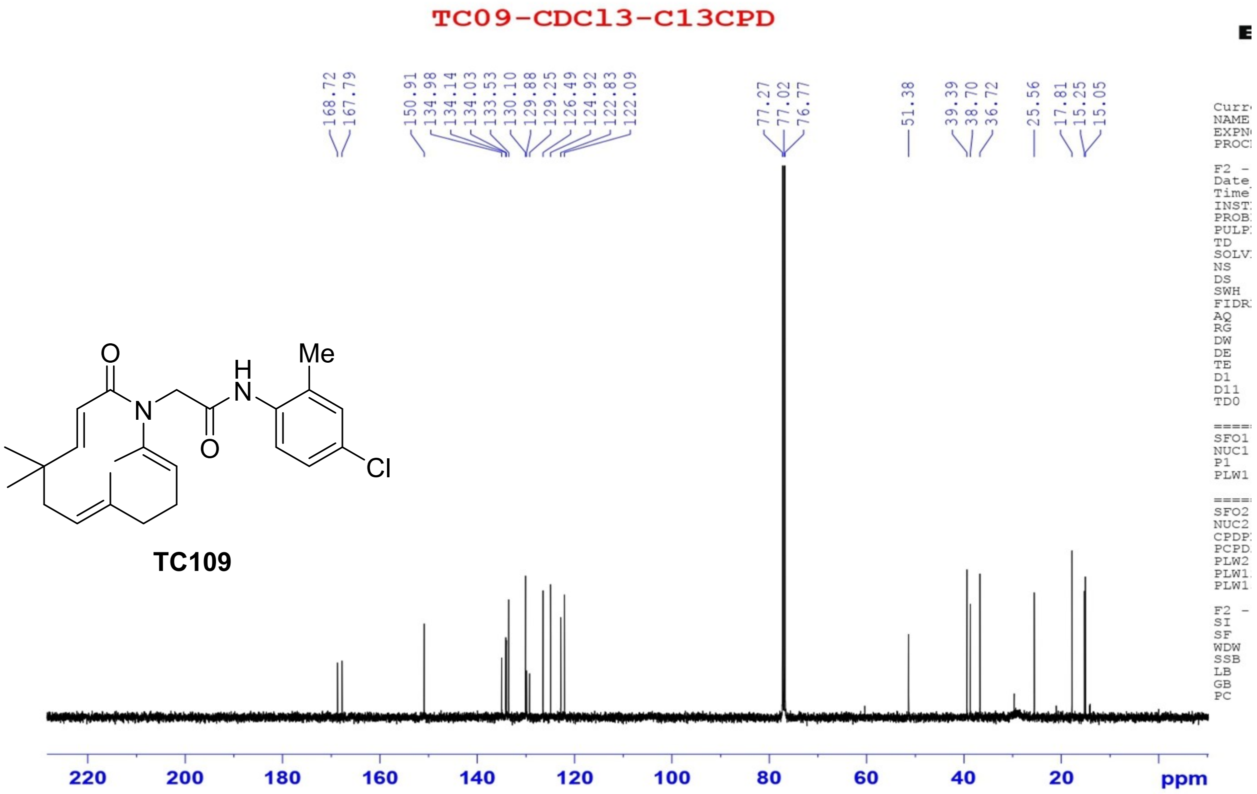


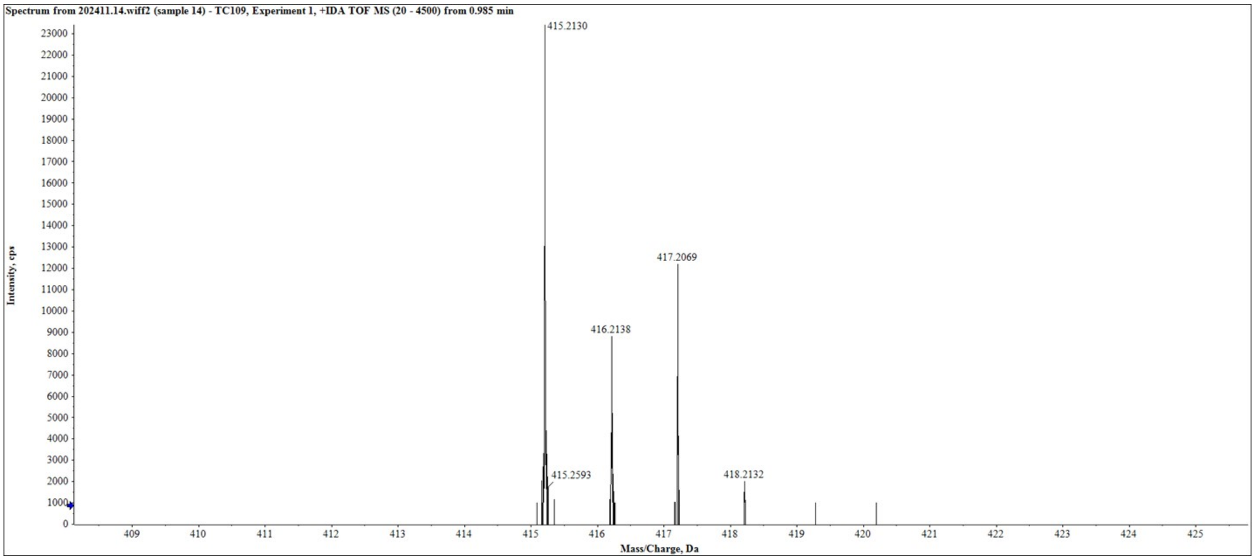


| Hit | Formula | m/z | RDB | ppm | MS Rank | MSMS ppm | MSMS Rank | Found |
|---|---|---|---|---|---|---|---|---|
| 1 | C24H31ClN2O2 | 415.21468 | 10.0 | -4.0 | 1 | | | NA/NA |

**Figure S36**. $^{13}$C NMR and HRMS spectra of compound **4i**

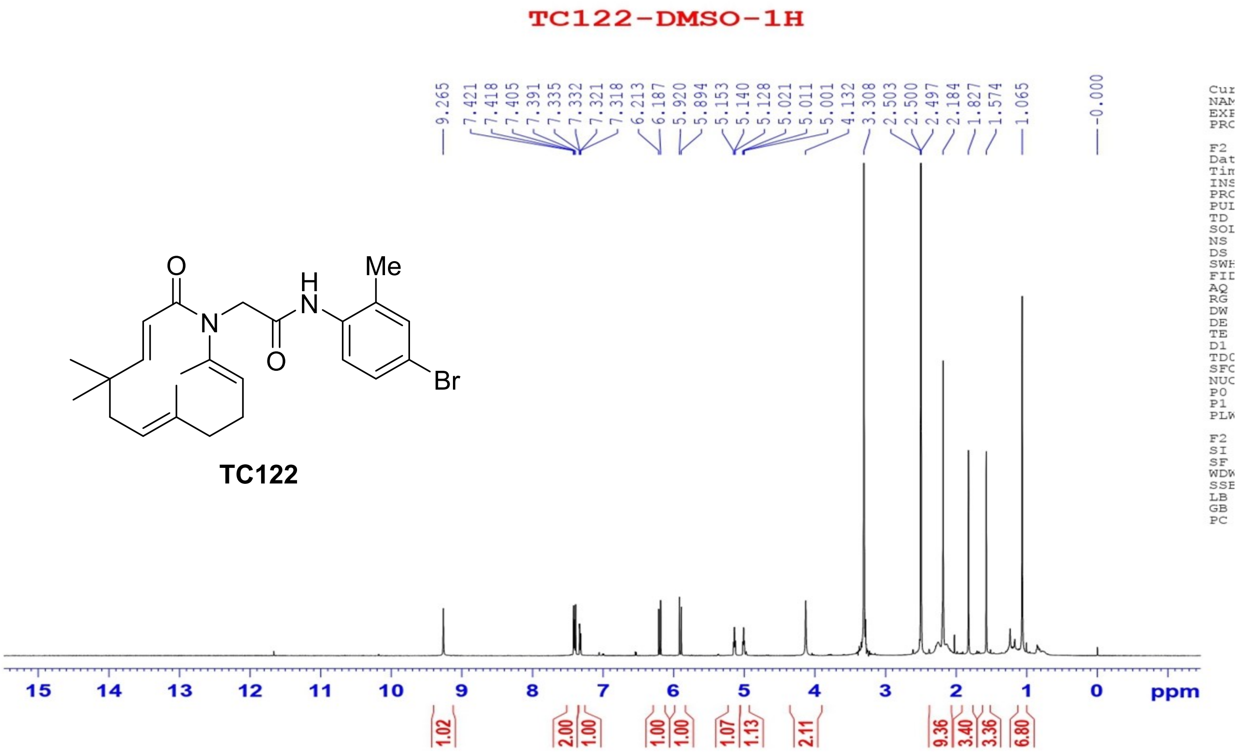
TC122-DMSO-1H
9.265
7.421
7.418
7.405
7.391
7.335
7.332
7.321
7.318
6.213
6.187
5.920
5.894
5.153
5.140
5.128
5.021
5.011
5.001
4.132
3.308
2.503
2.500
2.497
2.184
1.827
1.574
1.065
-0.000
O
N
H
N
Me
O
Br
TC122
15 14 13 12 11 10 9 8 7 6 5 4 3 2 1 0 ppm
1.02
2.00
1.00
1.00
1.00
1.07
1.13
2.11
9.36
3.40
3.36
6.80

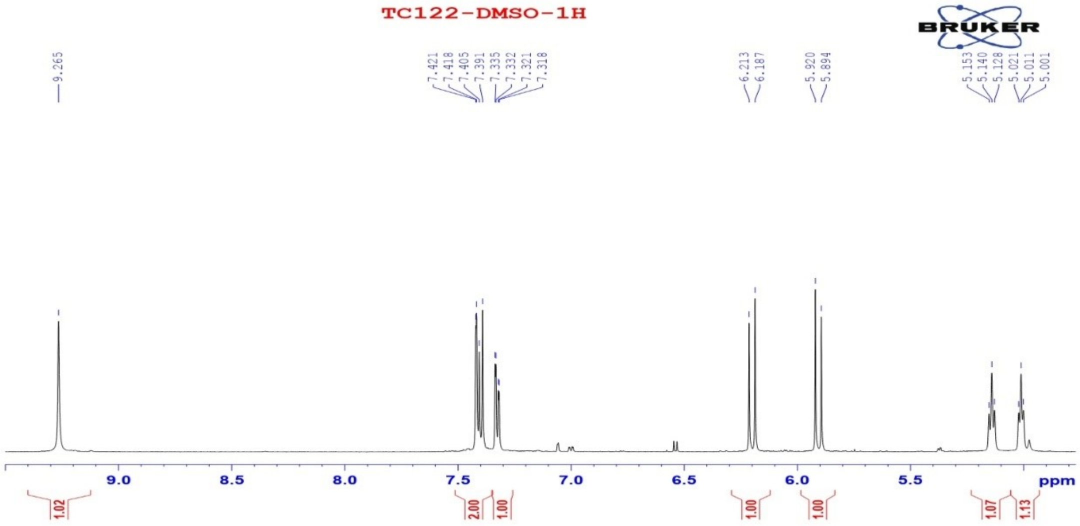


**Figure S37**. $^1$H NMR spectrum of compound **4j**

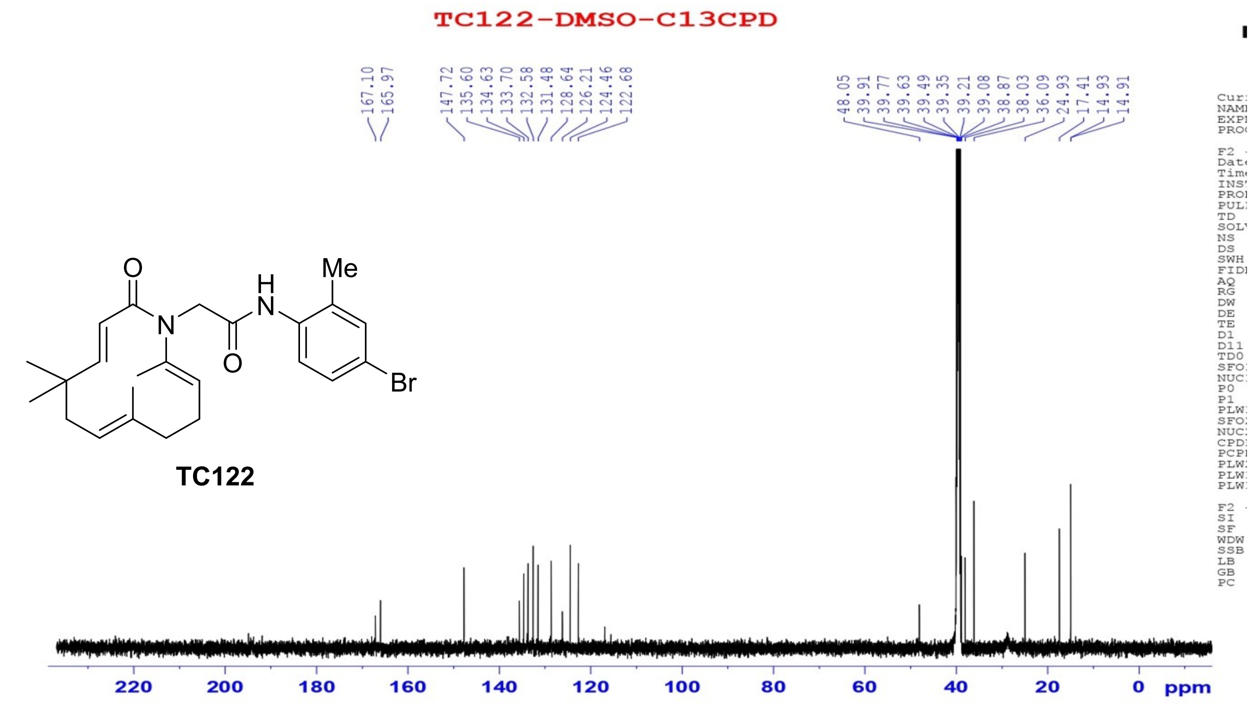


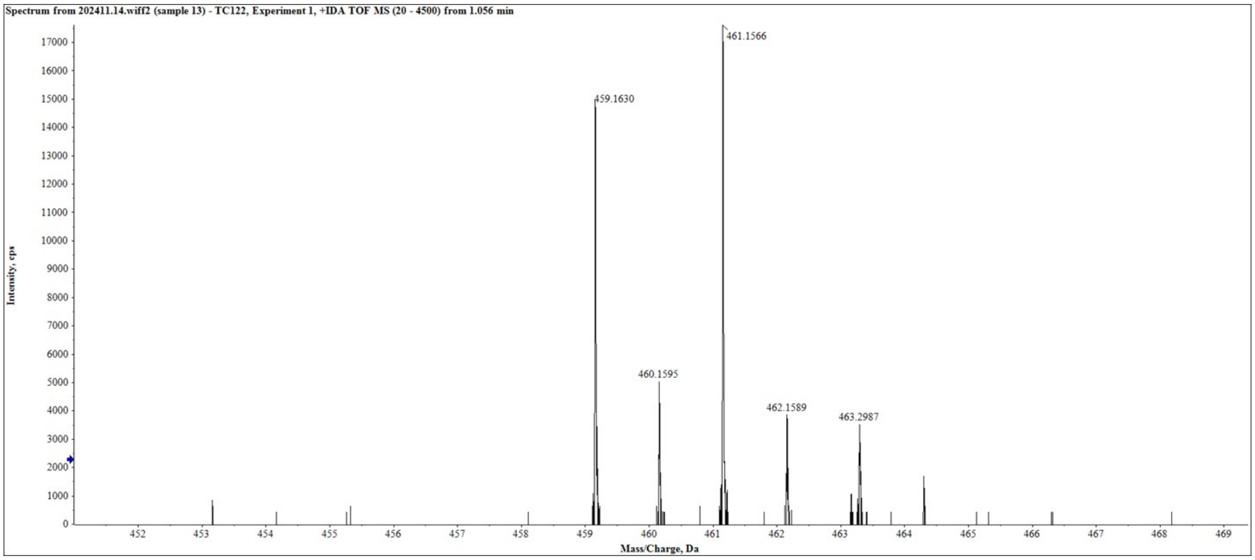


| Hit | Formula | m/z | RDB | ppm | MS Rank | MSMS ppm | MSMS Rank | Found |
|---|---|---|---|---|---|---|---|---|
| 1 | C24H31BrN2O2 | 459.16417 | 10.0 | -2.5 | 1 | | | NA/NA |

**Figure S38**. $^{13}$C NMR and HRMS spectra of compound **4j**

TC132-DMSO-1H

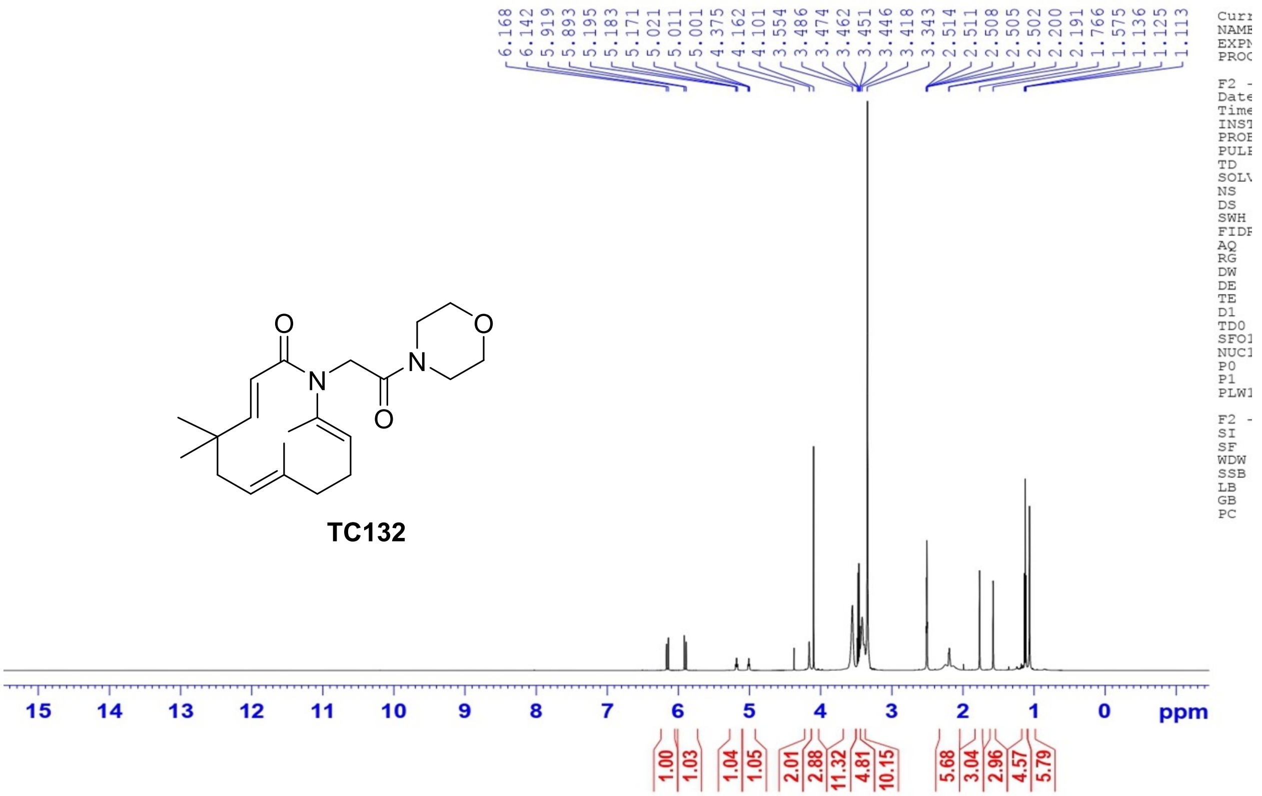

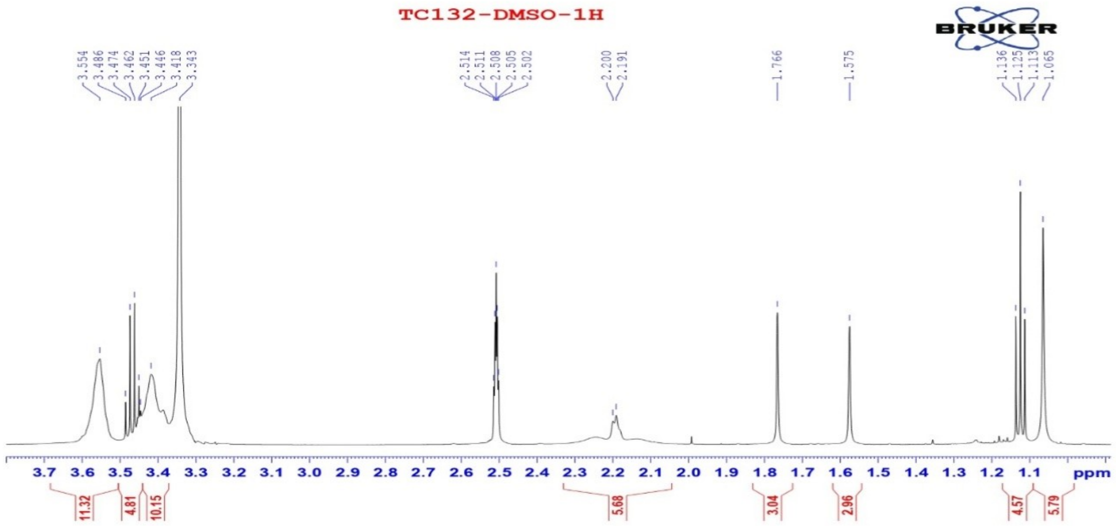


**Figure S39**. $^{1}$H NMR spectrum of compound **4k**

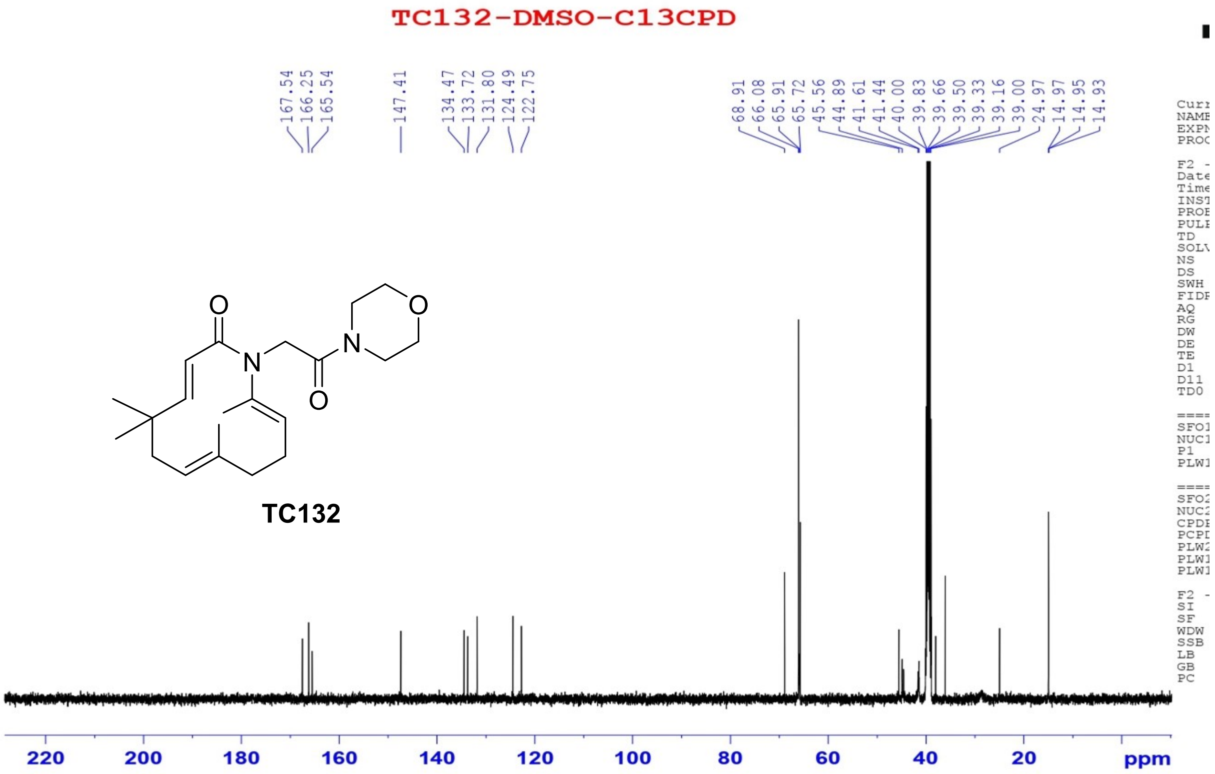


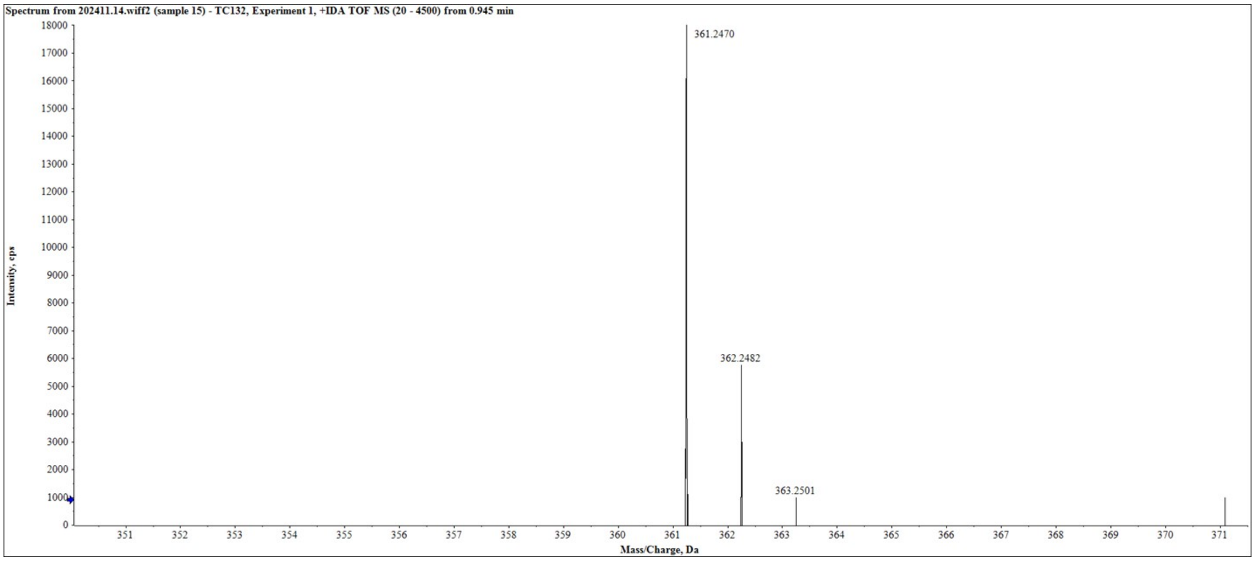


| Hit | Formula | m/z | RDB | ppm | MS Rank | MSMS ppm | MSMS Rank | Found |
|---|---|---|---|---|---|---|---|---|
| 1 | C21H32N2O3 | 361.24857 | 7.0 | -4.3 | 1 | | | NA/NA |

**Figure S40**. $^{13}$C NMR and HRMS spectra of compound **4k**

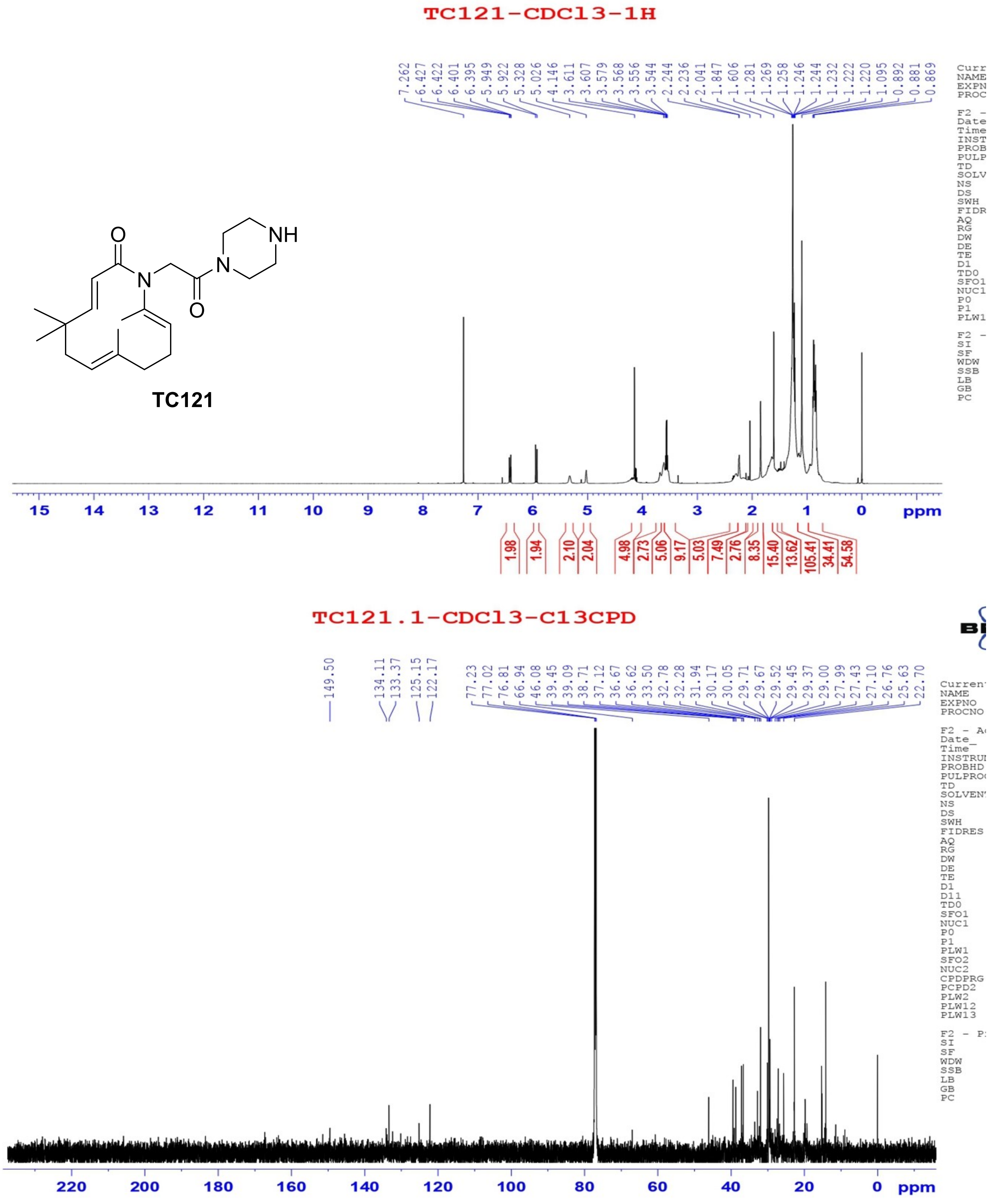


**Figure S41**. $^{1}H$ NMR and $^{13}C$ NMR spectra of compound **4l**

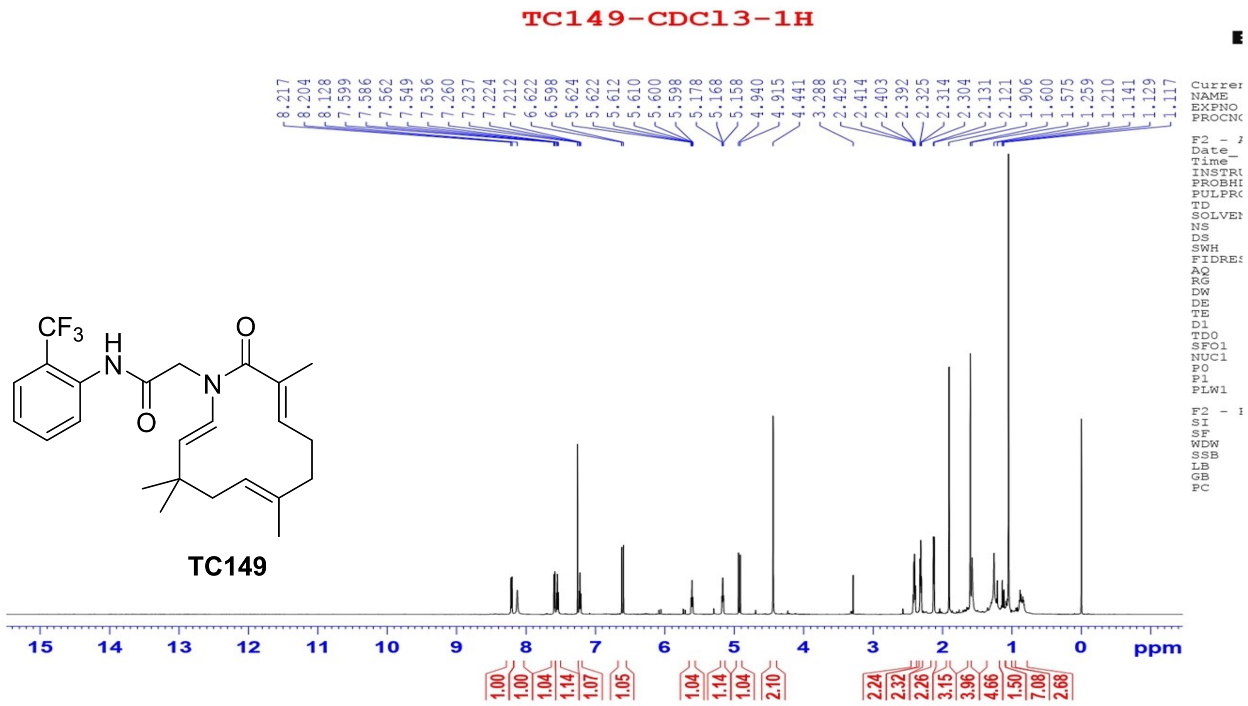
TC149-CDCl3-1H
8.217
8.204
8.128
7.599
7.586
7.562
7.549
7.536
7.260
7.237
7.224
7.212
6.622
6.598
5.624
5.622
5.612
5.610
5.600
5.598
5.178
5.168
5.158
4.940
4.915
4.441
3.288
2.425
2.414
2.403
2.392
2.325
2.314
2.304
2.131
2.121
1.906
1.600
1.575
1.259
1.210
1.141
1.129
1.117
CF3
H
N
O
N
O
TC149
15 14 13 12 11 10 9 8 7 6 5 4 3 2 1 0 ppm
1.00
1.00
1.04
1.14
1.07
1.05
1.04
1.14
1.04
2.10
2.24
2.32
2.26
3.15
3.96
4.66
1.50
7.08
2.68

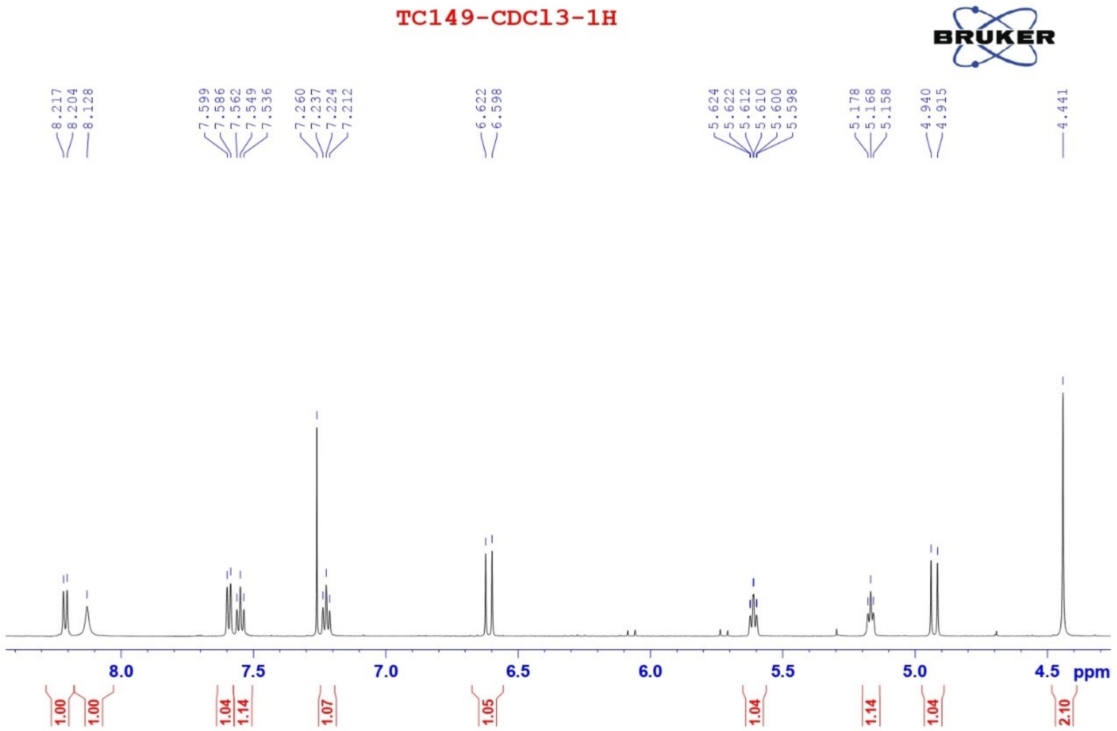


**Figure S42**. $^{1}$H NMR spectrum of compound **6a**

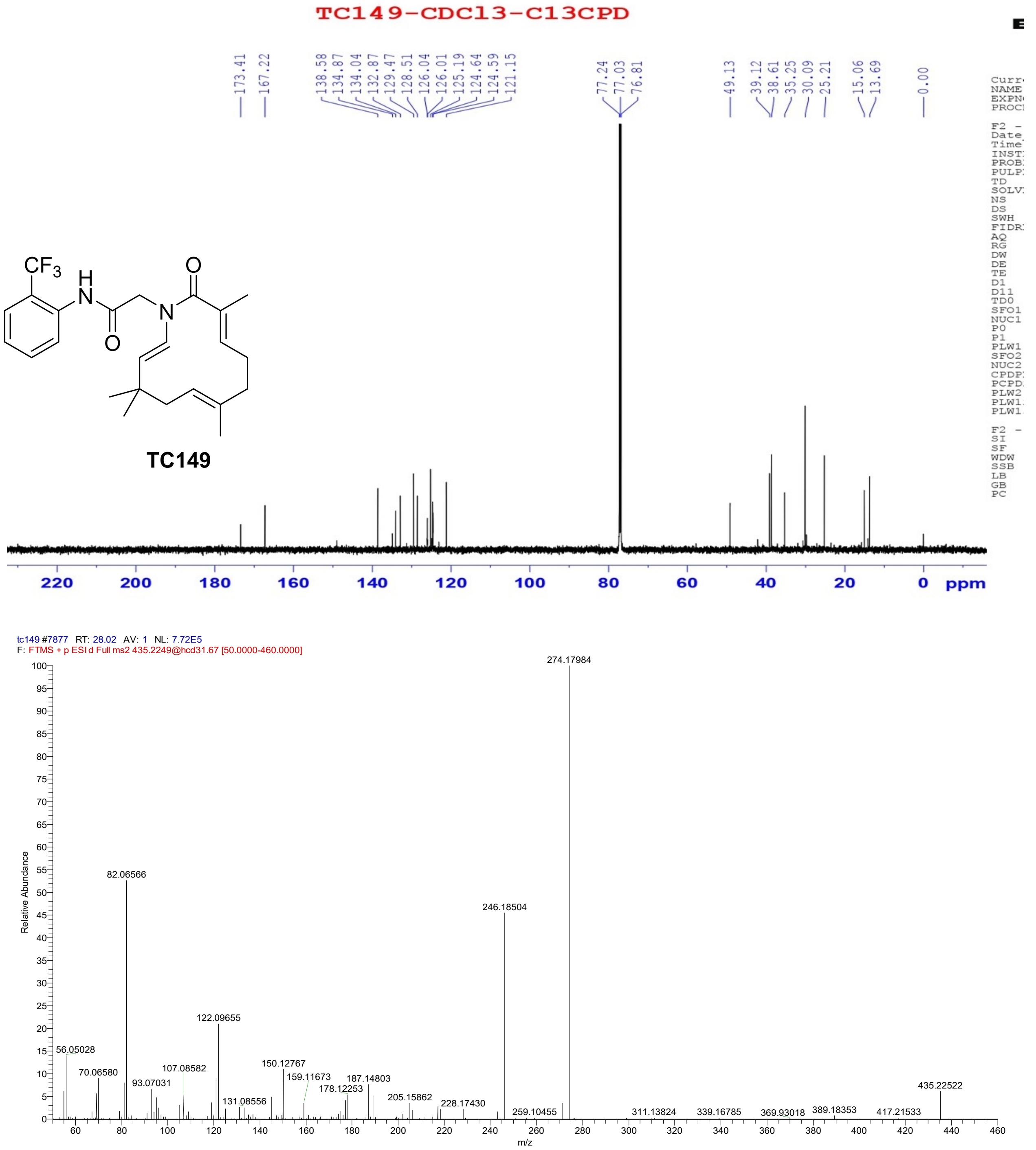


**Figure S43**. $^{13}C$ NMR and HRMS spectra of compound **6a**

TC152.1-CDCl3-1H

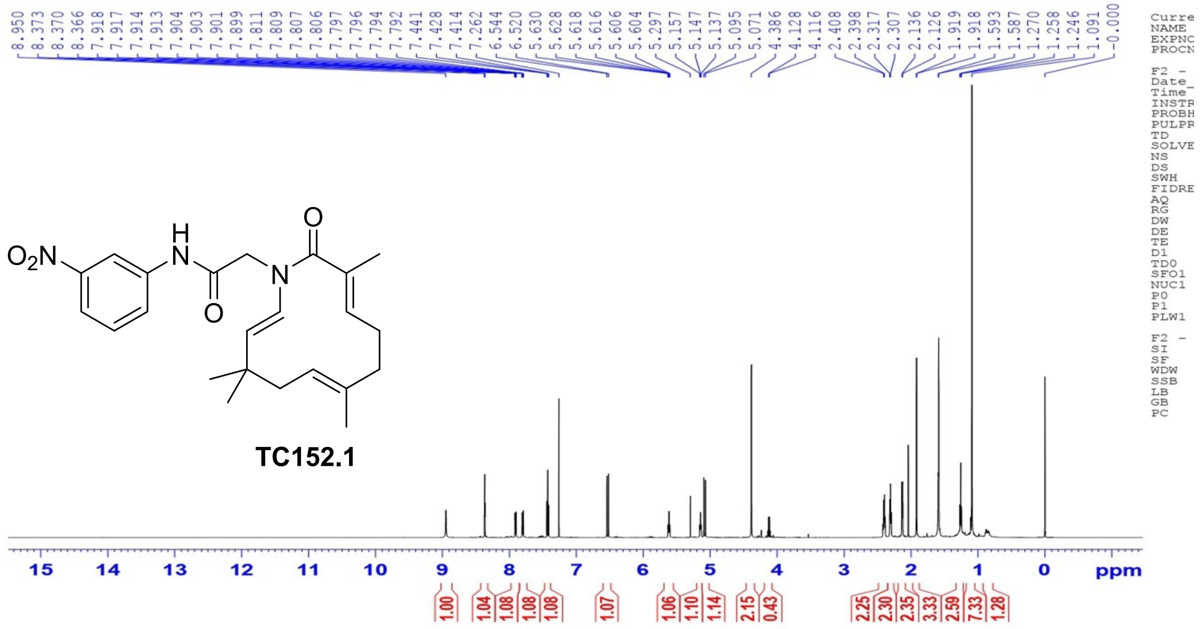

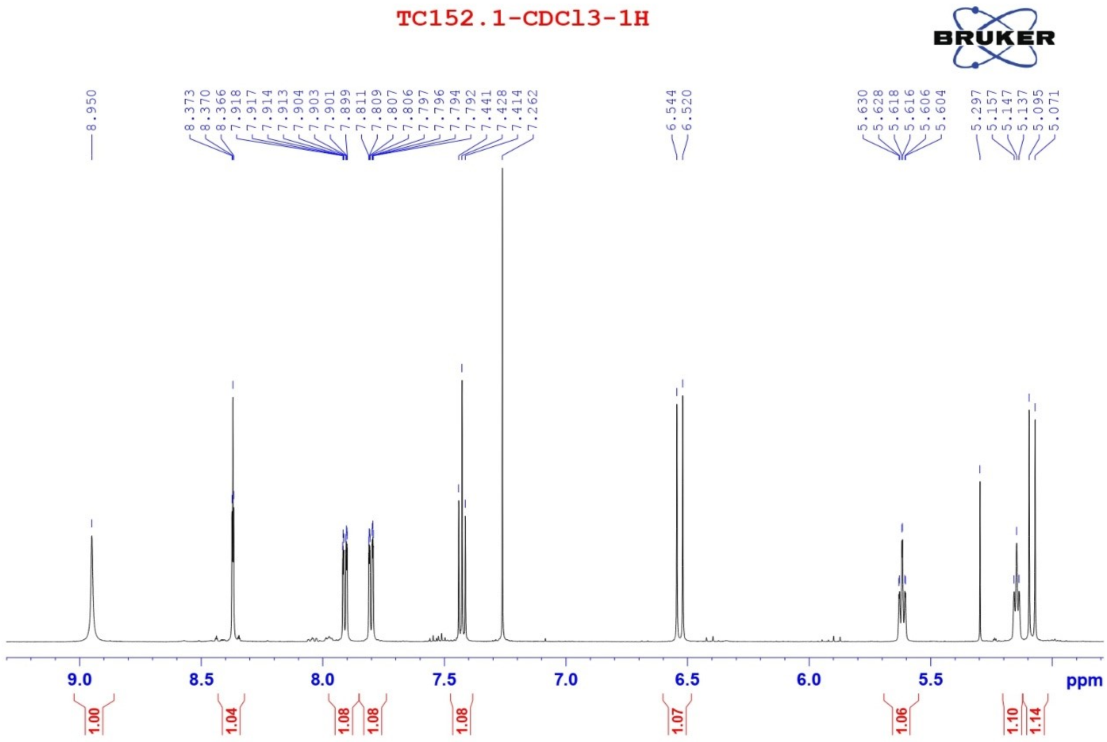


**Figure S44**. $^{1}$H NMR spectrum of compound **6c**

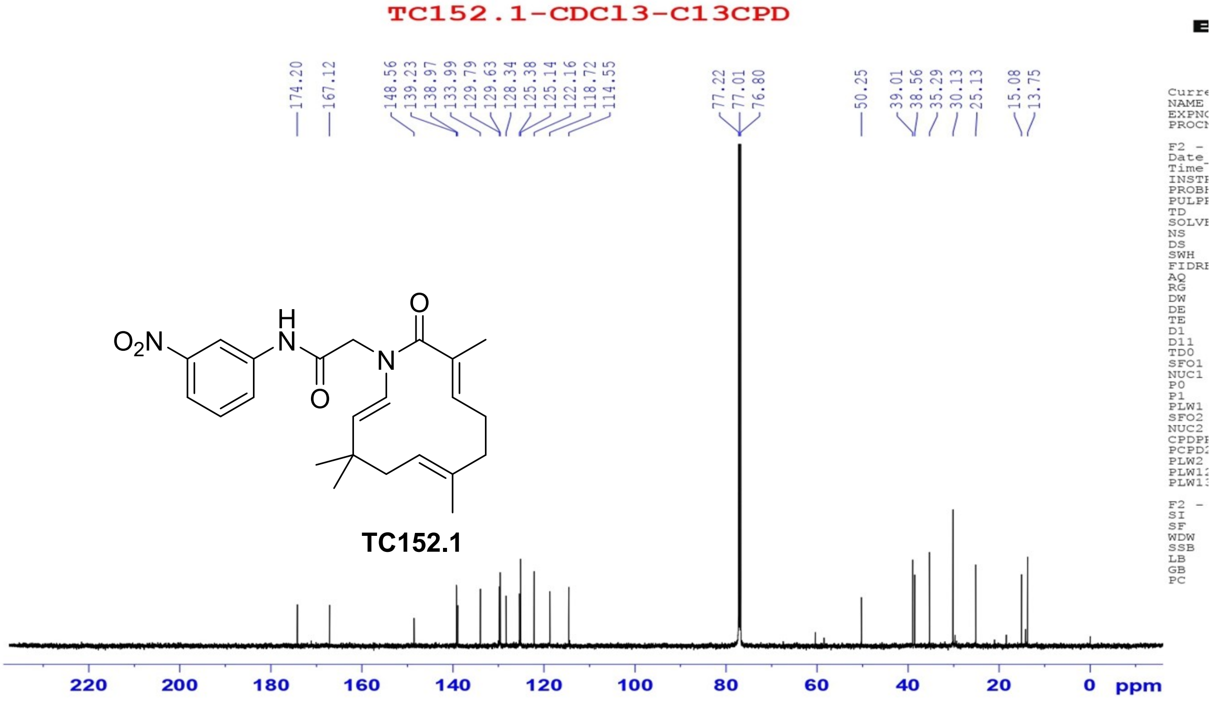


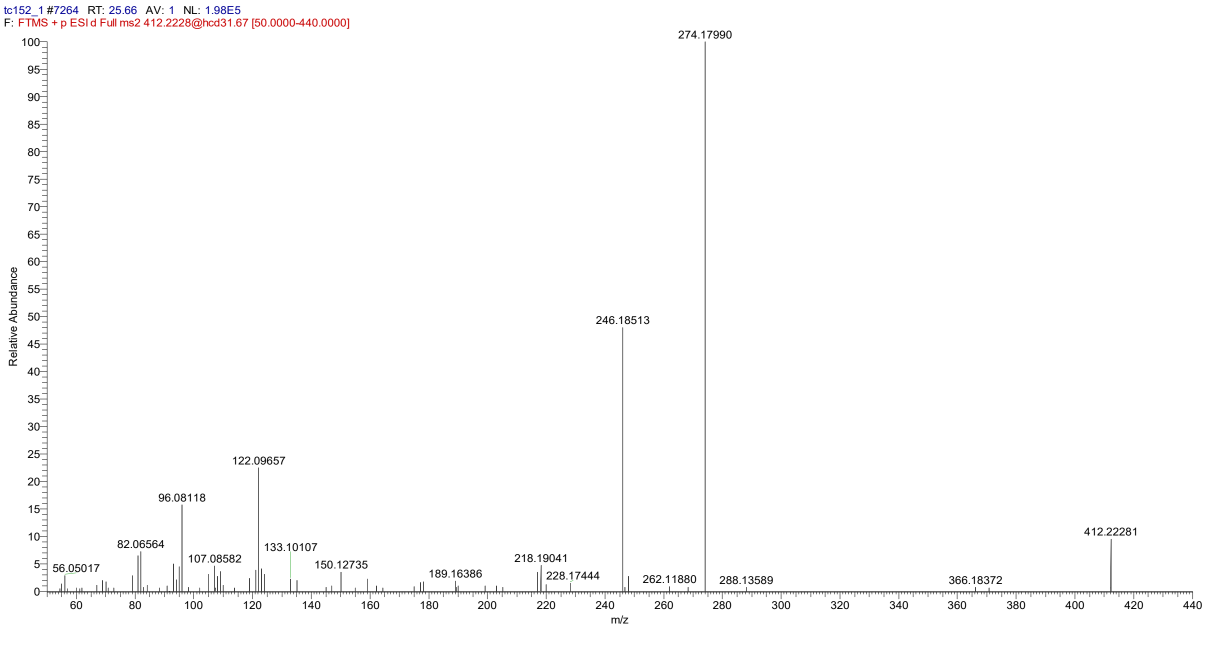


**Figure S45**. $^{13}$C NMR and HRMS spectra of compound **6c**

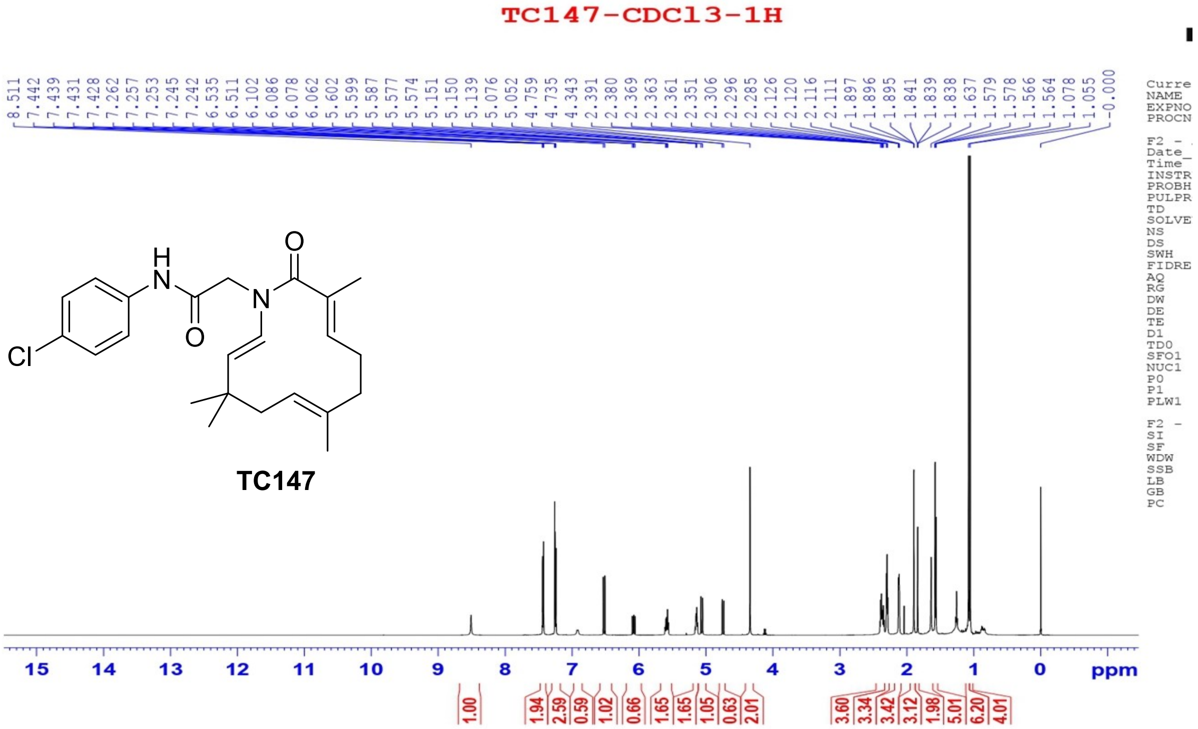

TC147-CDCl3-1H
8.511
7.442
7.439
7.431
7.428
7.262
7.257
7.253
7.245
7.242
6.535
6.511
6.102
6.086
6.078
6.062
5.602
5.599
5.587
5.577
5.574
5.151
5.150
5.139
5.076
5.052
4.759
4.735
4.343
2.391
2.380
2.369
2.363
2.361
2.351
2.306
2.296
2.285
2.126
2.120
2.116
2.111
1.897
1.896
1.895
1.841
1.839
1.838
1.637
1.579
1.578
1.566
1.564
1.078
1.055
-0.000
TC147
ppm

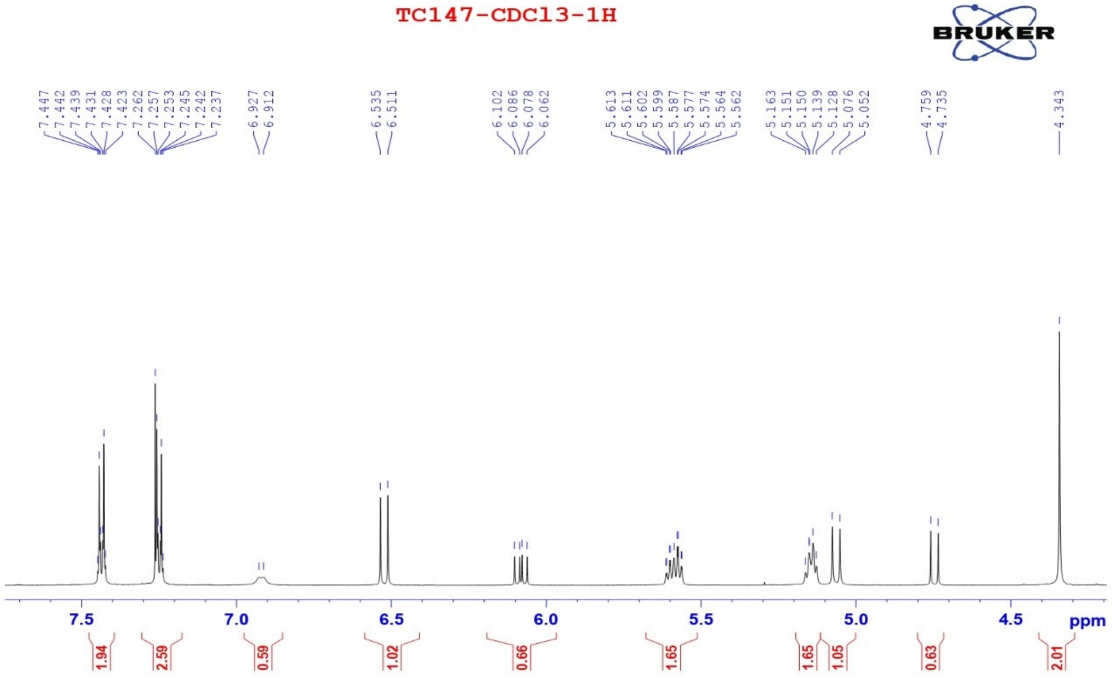


**Figure S46**. $^{1}$H NMR spectrum of compound **6e**

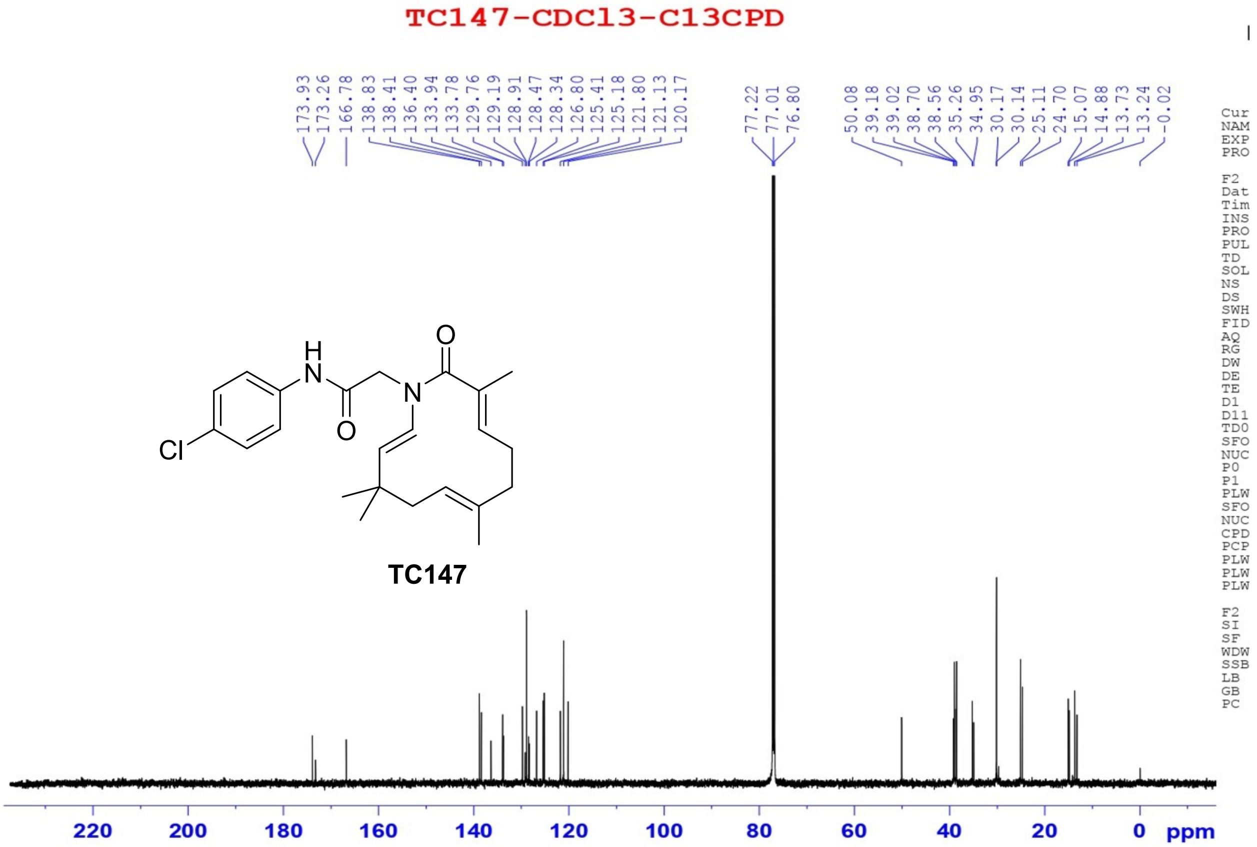


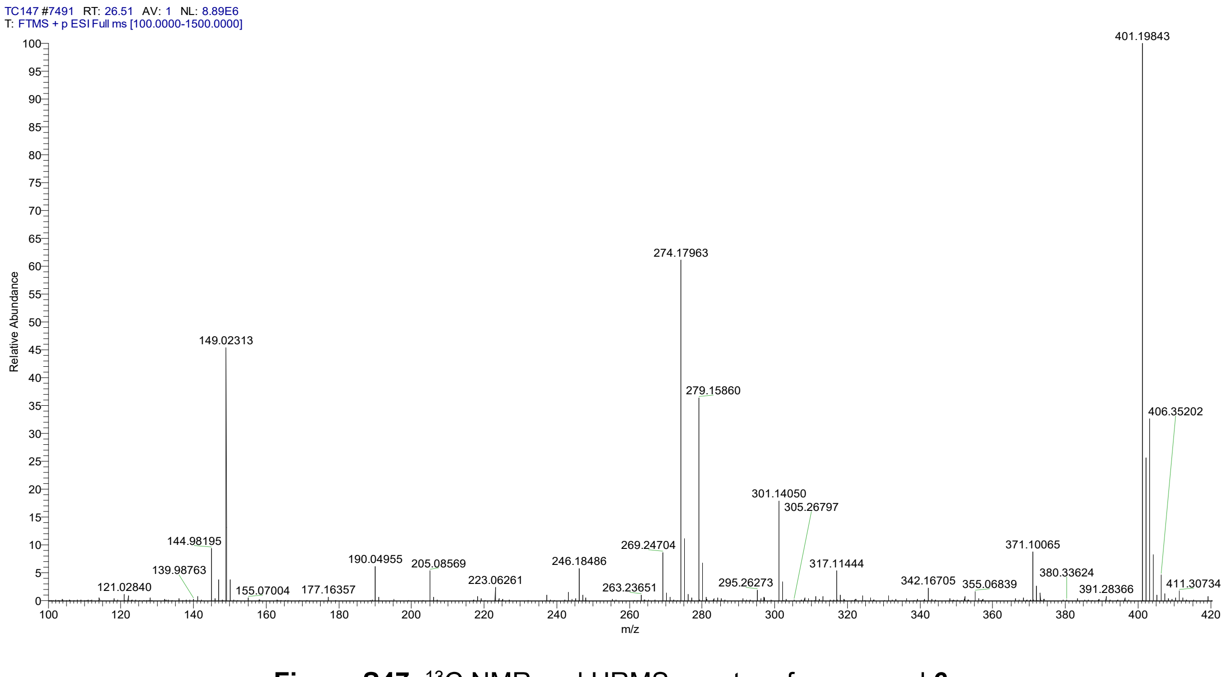


**Figure S47**. $^{13}$C NMR and HRMS spectra of compound **6e**

TC148-CDCl3-1H

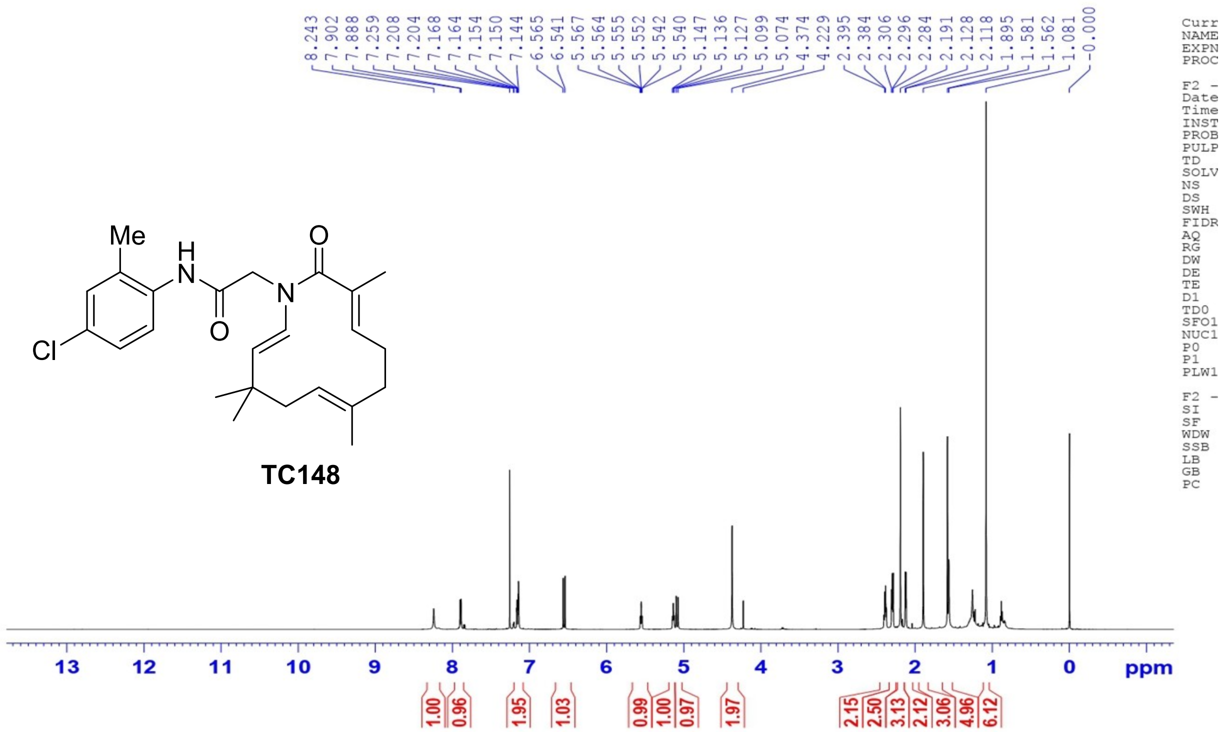

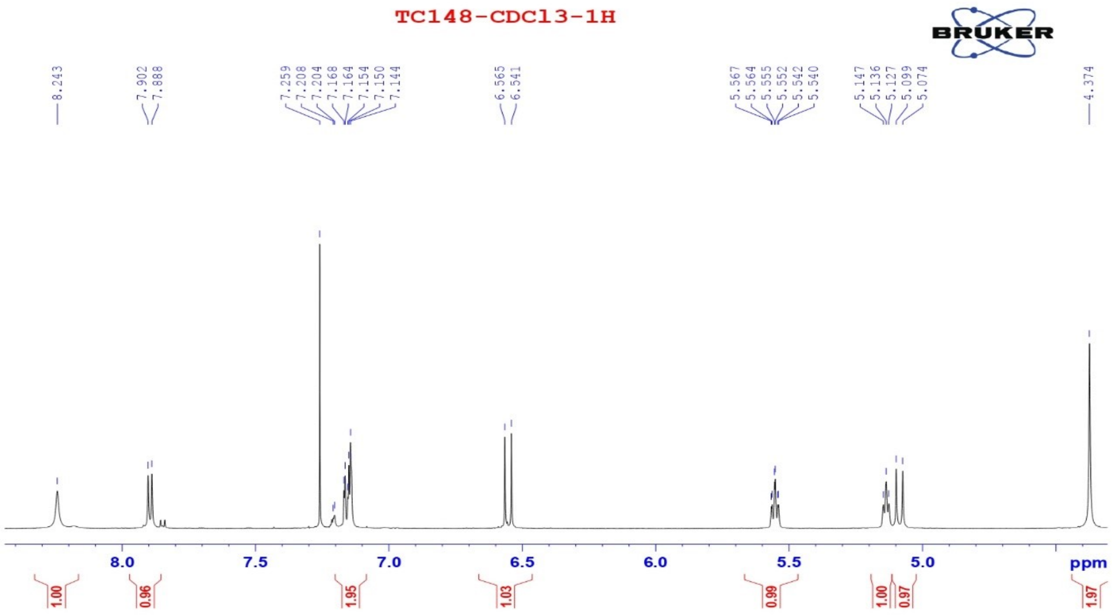


**Figure S48**. $^1$H NMR spectrum of compound **6i**

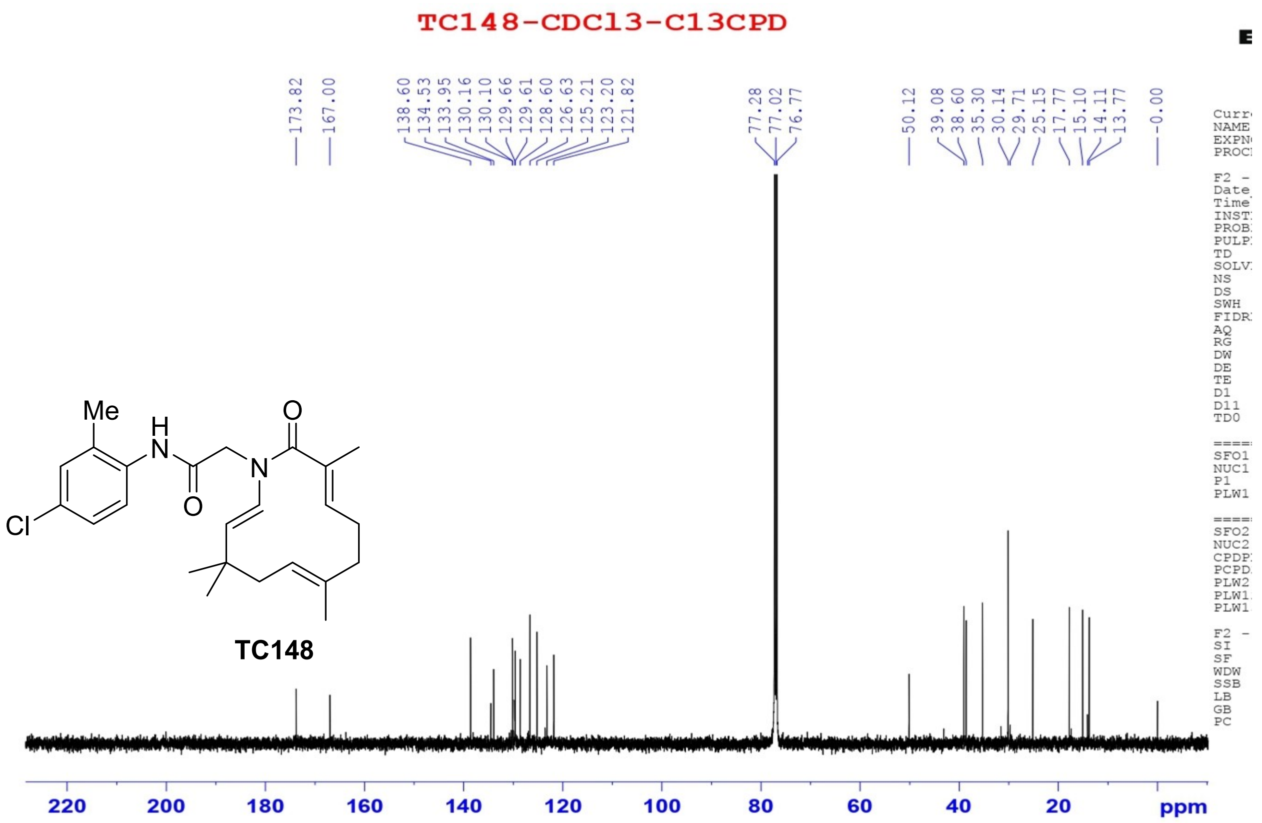


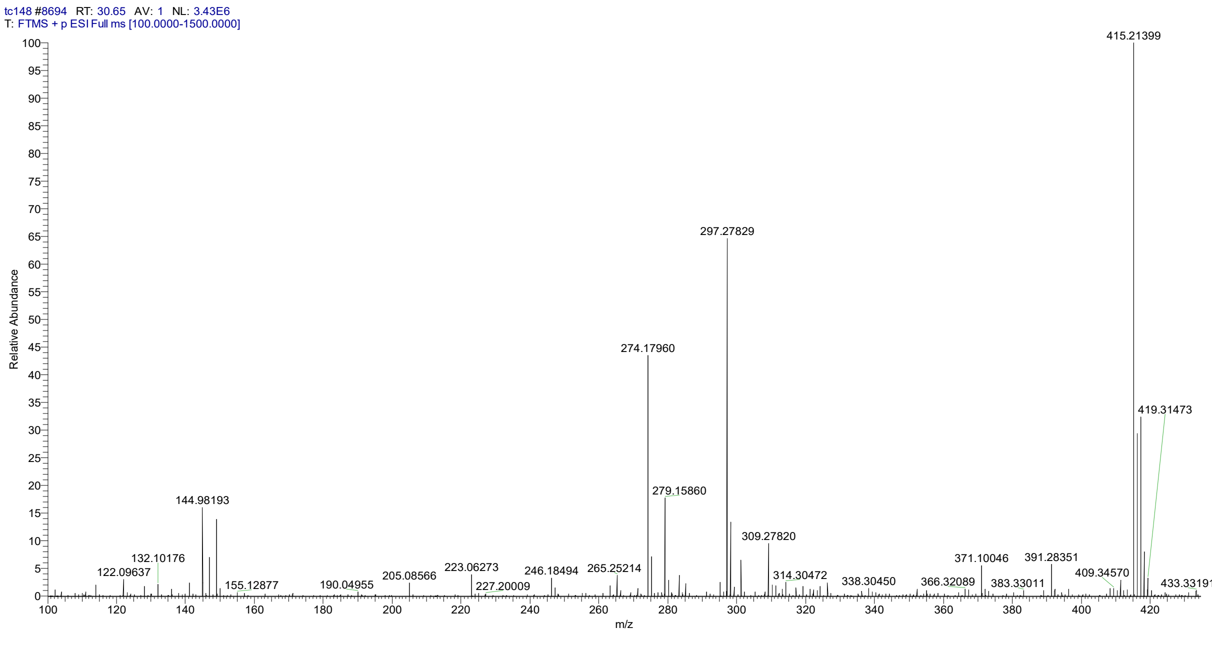


**Figure S49**. $^{13}$C NMR and HRMS spectra of compound **6i**

TC157-CDCl3-1H

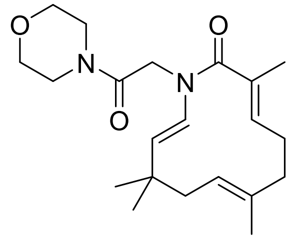

**TC157**

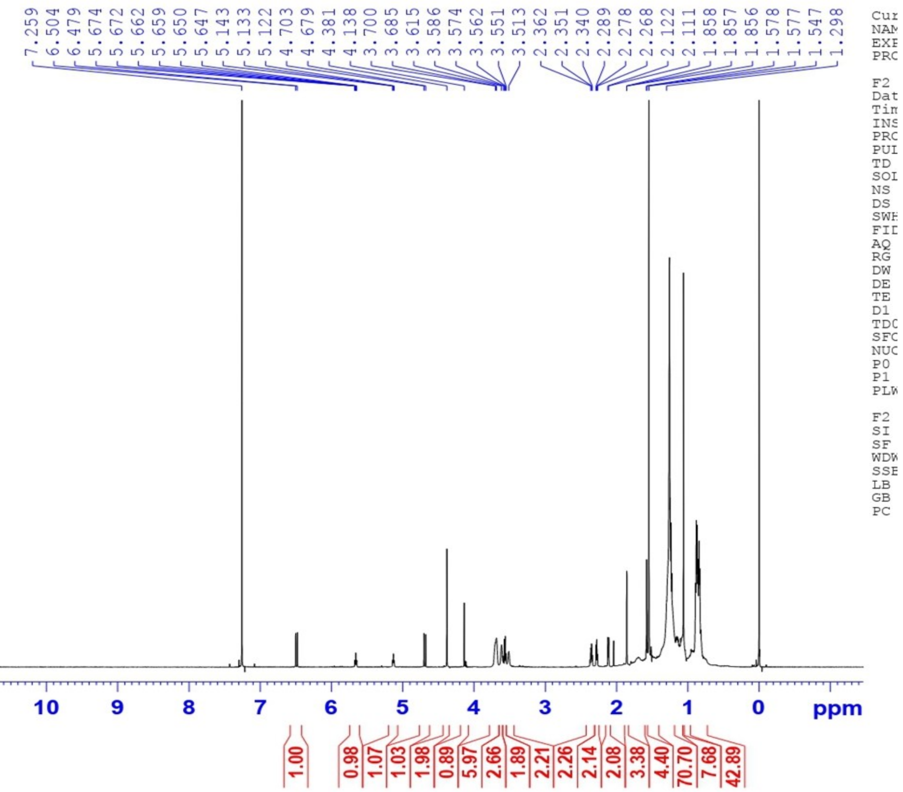

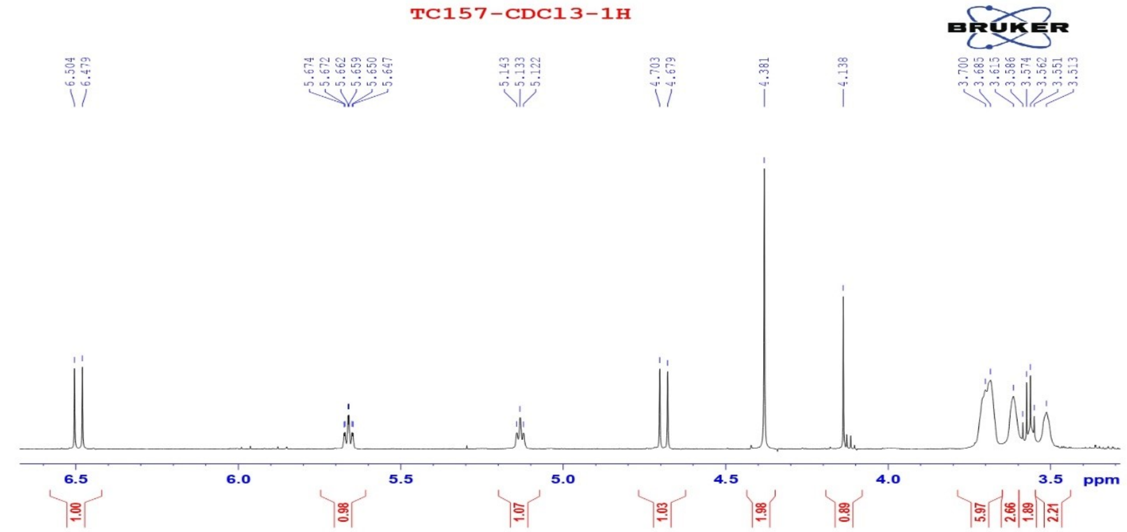


**Figure S50**. $^{1}$H NMR spectrum of compound **6k**

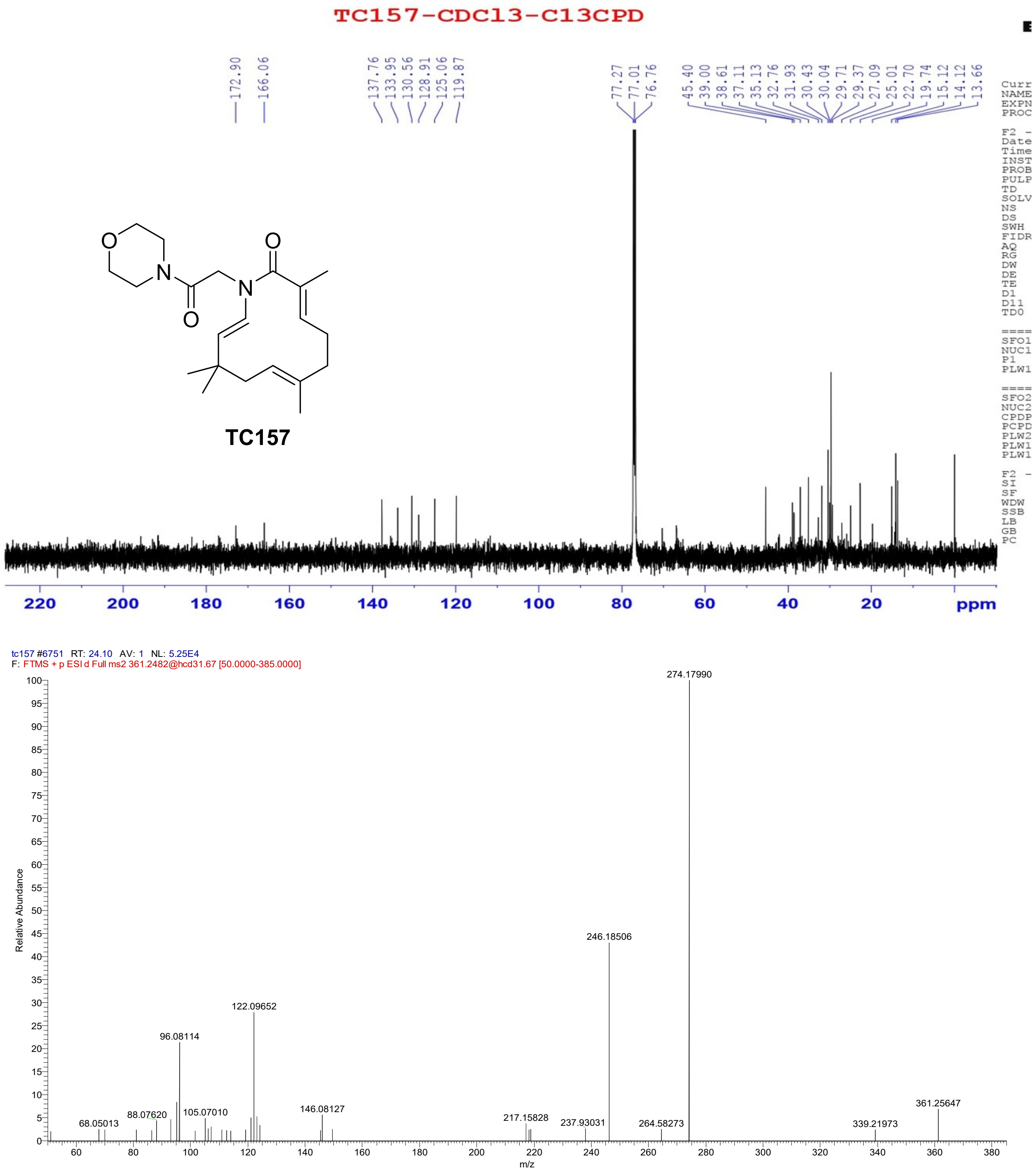


**Figure S51**. $^{13}C$ NMR and HRMS spectra of compound **6k**

**Table 1** The comparison between two methods to synthesize

| No | Compounds | Non-ultrasonic conditions | | Ultrasonic conditions | |
|---|---|---|---|---|---|
| | | **Time** | **Yield (%)[i]** | **Time** | **Yield (%)[i]** |
| 1 | **4a** | 36h | 81 | 2h | 76 |
| 2 | **4b** | 36h | 79 | 2h | 83 |
| 3 | **4c** | 36h | 80 | 2h | 82 |
| 4 | **4d** | 36h | 84 | 2h | 85 |
| 5 | **4e** | 36h | 85 | 2h | 83 |
| 6 | **4f** | 36h | 87 | 2h | 85 |
| 7 | **4g** | 36h | 80 | 2h | 83 |
| 8 | **4h** | 36h | 83 | 2h | 84 |
| 9 | **4i** | 36h | 80 | 2h | 79 |
| 10 | **4j** | 36h | 82 | 2h | 87 |
| 11 | **4k** | 36h | 69 | 2h | 71 |
| 12 | **4l** | 36h | 75 | 2h | 85 |
| 13 | **6a** | 36h | 87 | 2h | 89 |
| 14 | **6c** | 36h | 75 | 2h | 68 |
| 15 | **6e** | 36h | 89 | 2h | 88 |
| 16 | **6i** | 36h | 92 | 2h | 89 |
| 17 | **6k** | 36h | 77 | 2h | 69 |

[i]*following purification by column chromatography*

**Table 2** The cytotoxicity evaluation ($IC_{50}$) of novel zerumbone-amide derivatives.

| Compounds | $IC_{50}$ (μg/mL) | | | |
|---|---|---|---|---|
| | **HepG2** | **A549** | **HL-60** | **AGS** |
| **4a** | 7.81±0.25 | 7.49±0.24 | 7.91±0.47 | 8.28±0.43 |
| **4b** | **3.24±0.23** | **5.46±0.34** | 6.27±0.29 | 7.58±0.17 |
| **4c** | **1.81±0.17** | **3.22±0.27** | **3.98±0.32** | **4.14±0.44** |
| **4d** | 6.64±0.26 | **5.61±0.18** | 7.04±0.33 | 7.06±0.45 |
| **4e** | >100 | >100 | >100 | >100 |
| **4f** | >100 | >100 | >100 | >100 |
| **4g** | **2.16±0.16** | **1.88±0.12** | **2.24±0.13** | **2.12±0.15** |
| **4h** | 7.09±0.22 | 6.96±0.20 | 7.51±0.33 | 7.59±0.34 |
| **4i** | **0.81±0.04** | **1.21±0.05** | **0.96±0.10** | **1.10±0.06** |
| **4j** | **4.79±0.20** | **3.98±0.18** | **5.56±0.45** | **5.77±0.37** |
| **4k** | >100 | >100 | >100 | >100 |
| **4l** | **5.99±0.23** | **5.41±0.22** | **5.08±0.26** | **5.76±0.25** |
| **6a** | 46.83±3.26 | 39.84±2.49 | 49.56±2.75 | 64.92±2.51 |
| **6c** | 45.37±2.16 | 61.97±2.88 | 40.17±1.99 | 30.05±2.87 |
| **6e** | 54.90±3.13 | 58.95±3.90 | 43.31±1.93 | 56.05±4.02 |
| **6i** | 48.75±2.27 | 60.14±4.76 | 51.82±2.78 | 60.92±4.50 |
| **6k** | >100 | >100 | >100 | >100 |
| Azazerumbone 1 (**5**) | >100 | >100 | 63.43±2.30 | >100 |
| Azazerumbone 2 (**3**) | 27.40±2.43 | 56.90±3.04 | 11.84±1.20 | 39.64±2.21 |
| Zerumbone | **0.93±0.08** | **1.03±0.11** | **0.86±0.04** | **1.11±0.12** |
| Ellipticine | **0.33±0.02** | **0.39±0.03** | **0.35±0.03** | **0.40±0.04** |

**Co-ligand**

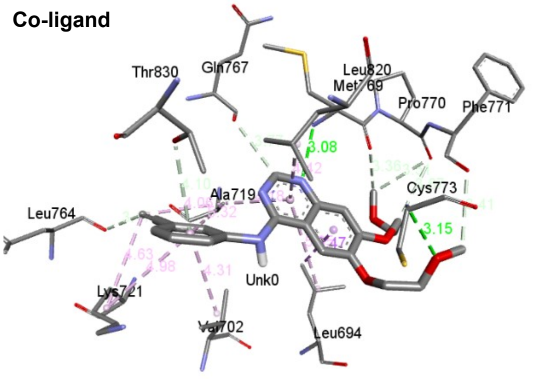


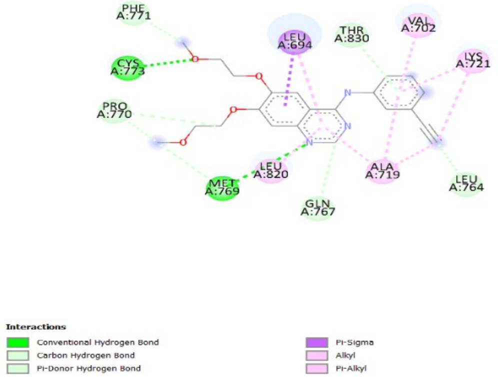


**Zerumbone**

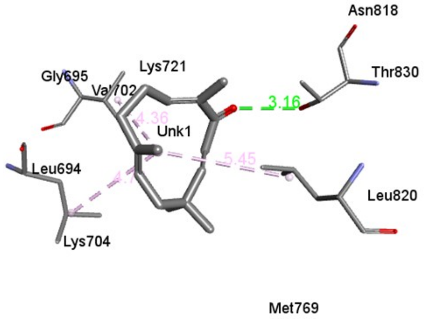


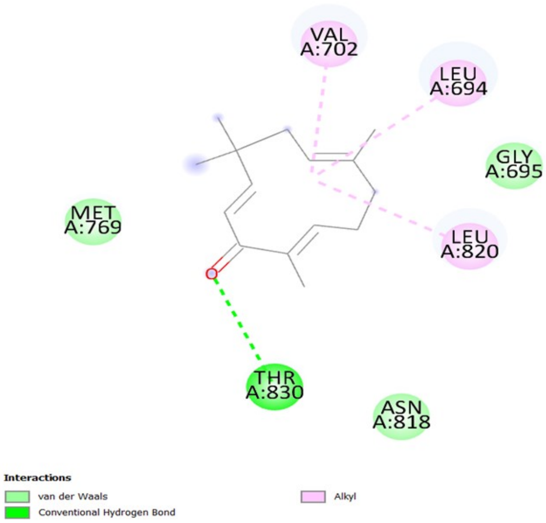

**4a**

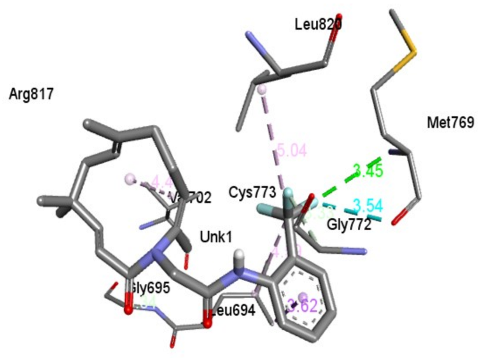
Leu820
Arg817
Met769
5.04
3.45
3.54
Cys773
Gly772
Unk1
Leu694
2.62

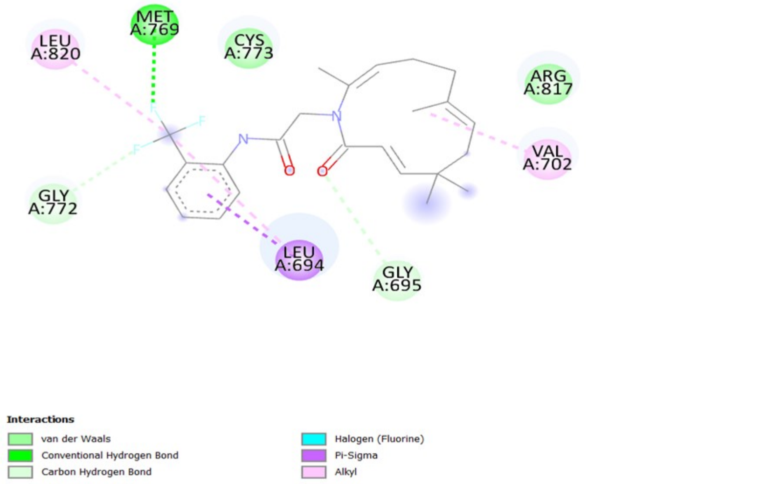
MET A:769
LEU A:820
CYS A:773
ARG A:817
VAL A:702
GLY A:772
LEU A:694
GLY A:695
Interactions
van der Waals
Conventional Hydrogen Bond
Carbon Hydrogen Bond
Halogen (Fluorine)
Pi-Sigma
Alkyl

**4b**

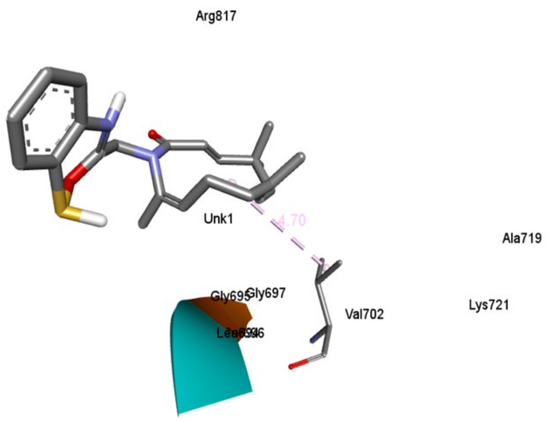
Arg817
Unk1
4.70
Ala719
Gly697
Val702
Lys721

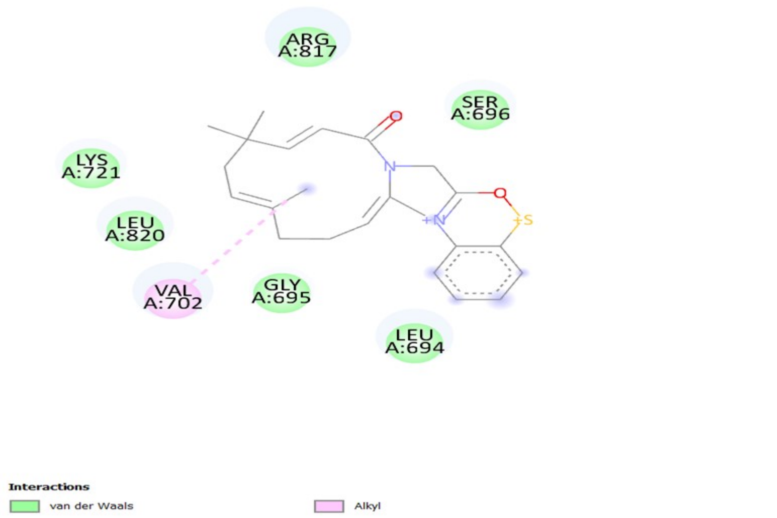
ARG A:817
SER A:696
LYS A:721
LEU A:820
VAL A:702
GLY A:695
LEU A:694
Interactions
van der Waals
Alkyl

**4c**

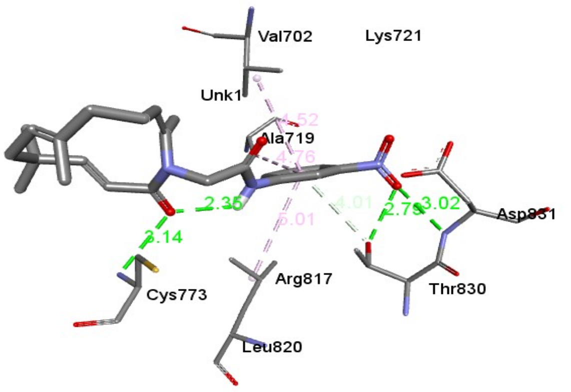
Val702
Lys721
Unk1
Ala719
Asp831
2.35
3.14
3.02
4.01
4.52
4.76
5.01
Arg817
Cys773
Thr830
Leu820

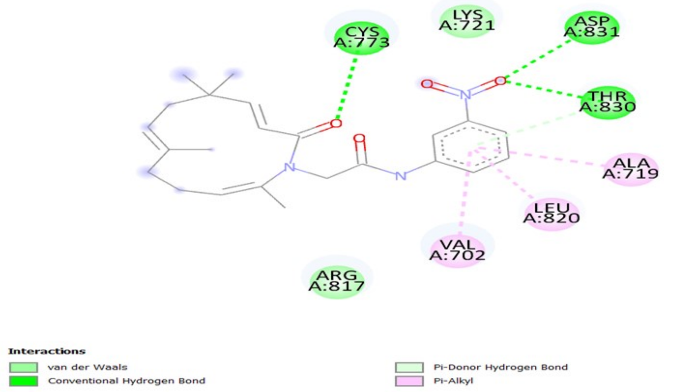
CYS A:773
LYS A:721
ASP A:831
THR A:830
ALA A:719
LEU A:820
VAL A:702
ARG A:817
Interactions
van der Waals
Conventional Hydrogen Bond
Pi-Donor Hydrogen Bond
Pi-Alkyl

**4d**

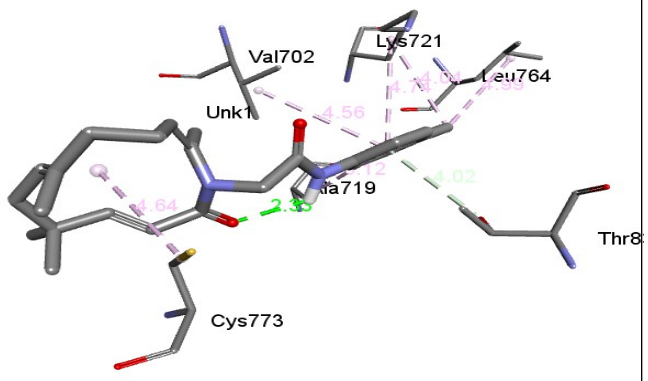
Val702
Lys721
Leu764
Unk1
4.56
4.64
4.02
Cys773

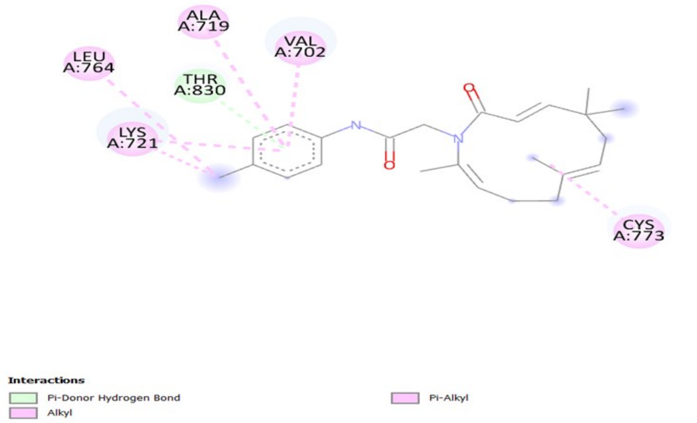
ALA A:719
VAL A:702
LEU A:764
THR A:830
LYS A:721
CYS A:773
Interactions
Pi-Donor Hydrogen Bond
Alkyl
Pi-Alkyl

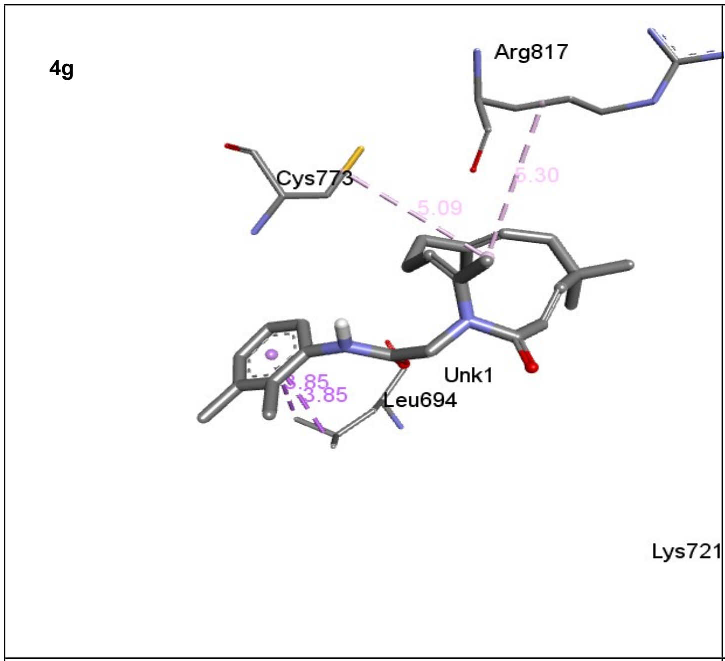
4g
Arg817
Cys773
5.09
5.30
Unk1
Leu694
3.85
3.85
Lys721

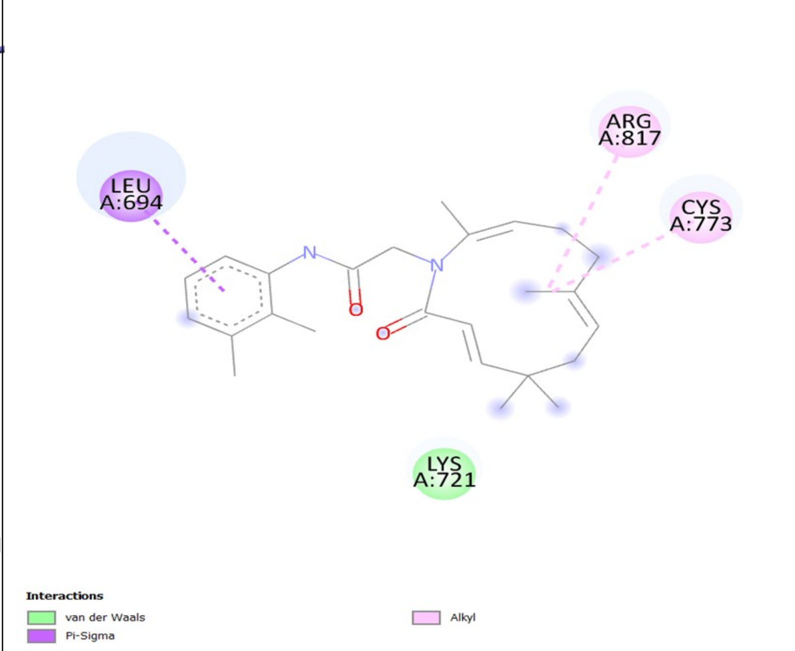
LEU A:694
ARG A:817
CYS A:773
LYS A:721
Interactions
van der Waals
Pi-Sigma
Alkyl

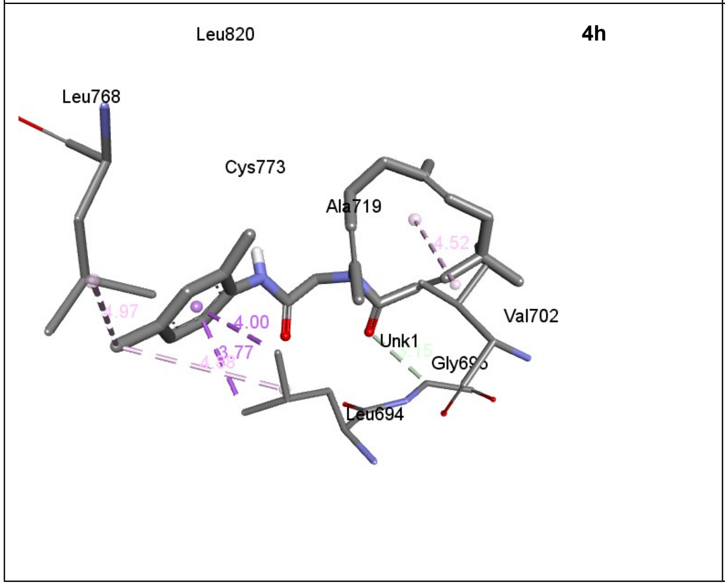
Leu820
4h
Leu768
Cys773
Ala719
4.52
Val702
4.00
3.77
Unk1
Leu694

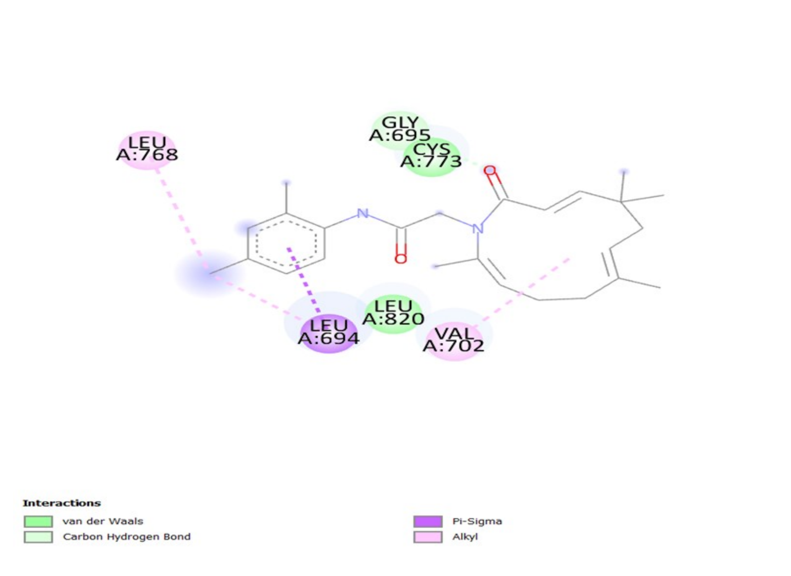
LEU A:768
GLY A:695
CYS A:773
LEU A:694
LEU A:820
VAL A:702
Interactions
van der Waals
Carbon Hydrogen Bond
Pi-Sigma
Alkyl

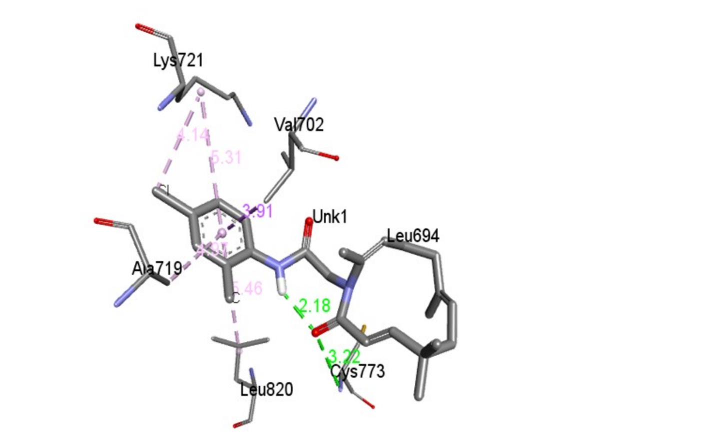
Lys721
Val702
Unk1
Leu694
Ala719
2.18
Cys773
Leu820

4i

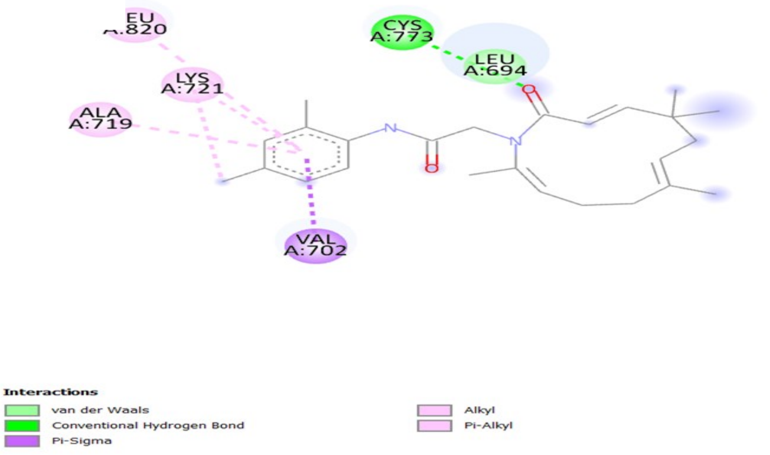
CYS A:773
LEU A:694
LYS A:721
ALA A:719
VAL A:702
Interactions
van der Waals
Conventional Hydrogen Bond
Pi-Sigma
Alkyl
Pi-Alkyl

4j

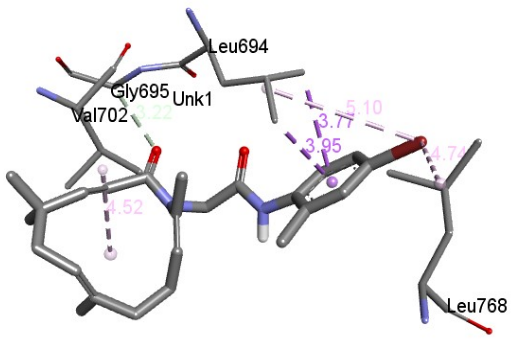
Leu694
Gly695
Unk1
Val702
5.10
3.77
3.95
4.52
Leu768

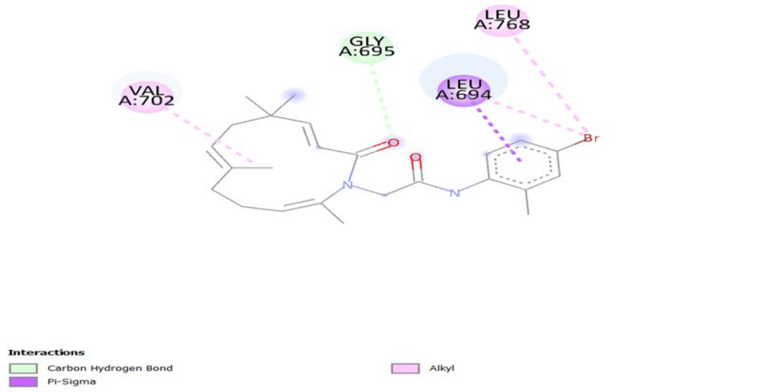
GLY A:695
LEU A:768
VAL A:702
LEU A:694
Br
Interactions
Carbon Hydrogen Bond
Pi-Sigma
Alkyl

4l

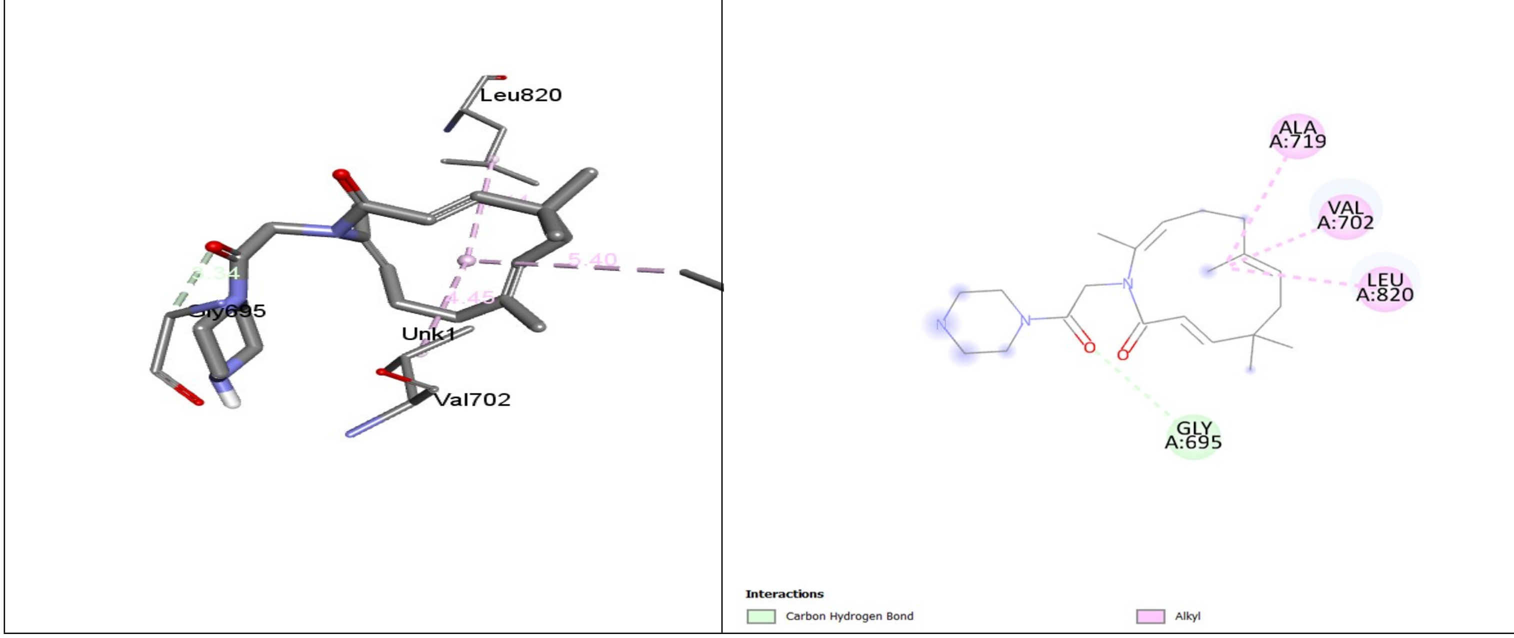


**Fig 1** Binding interactions of ligands into the active site of receptor: 3D (left) and 2D (right)

**Table 3** Binding energy and bond interactions

| **Entry** | **Binding energy ($kcal.mol^{-1}$)** | Interacting Amino Acid | | **Total interactions** |
|---|---|---|---|---|
| | | Zerumbone part | **Attached group** | |
| Zerumbone | -6.4 | Val702, Thr830, Leu694, Leu820 | | 4 |
| 4a | -6.9 | Val702, Gly695 | Met769, Leu694, Leu820, Gly772 | 7 |
| 4b | -7.1 | Val702 | | 1 |
| 4c | **-8.0** | Cys773 | Asp831, Thr830, Ala719, Leu820, Val702 | 7 |
| 4d | -7.3 | Cys773 | Val702, Thr830, Lys721, Leu764, Ala719 | 7 |
| 4g | **-7.8** | Arg817, Cys773 | Leu694 | 3 |
| 4h | -6.9 | Val702 | Leu694, Leu768 | 4 |
| 4i | **-8.1** | Cys773 | Val702, Lys721, Leu820, Ala719 | 6 |
| 4j | -7.3 | Val702, Gly695 | Leu694, Leu768 | 5 |
| 4l | -5.8 | Val702, Leu820, Ala719 | Gly695 | 4 |
| Coligand (erlotinib) | -7.8 | Met769, Cys773, Val702, Thr830, Lys721, Leu764, Ala719, Leu694, Gln767, Leu820, Pro770, Phe771. | | 18 |

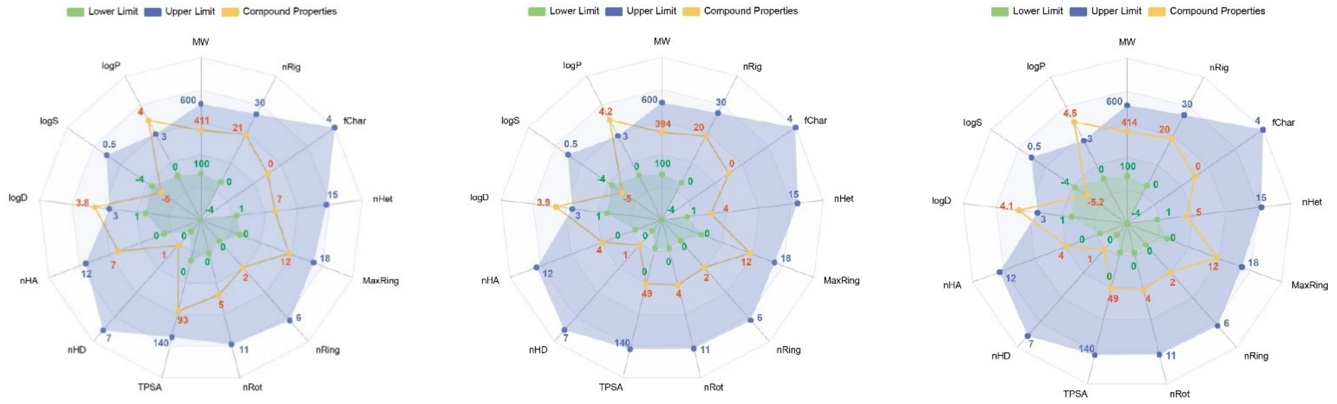


**4c** **4g** **4i**

**Figure 2. Radar diagram illustrating the physicochemical characteristics of compounds 4c, 4g, and 4i**. *MW: molecular weight; nRig: number of rigid bonds; fChar: formal charge; nHet: number of heteroatoms; MaxRing: number of atoms in the largest ring; nRing: number of rings; nRot: number of rotatable bonds; TPSA: topological polar surface area (Å²); nHD: number of hydrogen bond donors; nHA: number of hydrogen bond acceptors; logD: logarithm of the octanol-water partition coefficient at physiological pH 7.4; logS: logarithm of aqueous solubility (mol·L⁻¹); and logP: logarithm of the octanol-water partition coefficient.*

**Table 4.** Prediction of ADMET properties of the compounds using pkCSM and Swiss ADME.

| **Drug likeness** | **4c** | **4g** | **4i** |
|---|---|---|---|
| Lipinski | yes | yes | yes |
| Goshe | yes | yes | yes |
| Veber | yes | yes | yes |
| Egan | yes | yes | yes |
| Muegge | yes | No; 1 violation: XLOGP3>5 | No; 1 violation: XLOGP3>5 |
| Bioavailability score | 0.55 | 0.55 | 0.55 |
| **Absorption** | | | |
| Log S (mol/L) | -6.189 | -6.079 | -6.373 |
| (Intestinal absorption (human) (% Absorbed) | 85.96 | 91.17 | 89.08 |
| Caco-2 perm. (log Papp in $10^{-6}$ cm/ s | 0.786 | 1.26 | 1.25 |
| **Distribution** | | | |
| VDss (log L/kg) | 0.17 | 0.463 | 0.347 |
| Fract. Unb. (Fu) | 0 | 0.006 | 0.002 |
| BBB perm. (log BB) | 0.014 | 0.41 | 0.421 |
| **Metabolism** | CYP3A4 substrate<br>CYP2C19 inhibitor<br>CYP2C9 inhibitor<br>CYP3A4 inhibitor | CYP3A4 substrate | CYP3A4 substrate |
| **Excretion** | | | |
| Total clearance Log(ml/min/kg) | 1.169 | 1.191 | -0.322 |
| Half-life (T1/2) | 0.414 | 0.26 | 0.308 |
| **Toxicity** | | | |
| AMES toxicity | No | No | No |
| Rat oral acute toxicity | No (0.051) | No (0.029) | No (0.035) |
| Skin sensitization | yes | yes | yes |
| hERG I inhibitor | No | No | No |
| hERG II inhibitor | No | No | No |
| EyeCorrosion | No | No | No |

| | | | |
|---|---|---|---|
| Respiratory | yes | Moderate | Moderate |
| Eye Irritation | yes | yes | Moderate |
